# Focal Molography Allows for Affinity and Concentration Measurements of Proteins in Complex Matrices with High Accuracy

**DOI:** 10.3390/bios15020066

**Published:** 2025-01-22

**Authors:** Lorin Dirscherl, Laura S. Merz, Ronya Kobras, Peter Spies, Andreas Frutiger, Volker Gatterdam, Dominik M. Meinel

**Affiliations:** 1 Institute for Chemistry and Bioanalytics, School of Life Sciences, University of Applied Sciences and Arts Northwestern Switzerland, FHNW, Hofackerstrasse 30, 4132 Muttenz, Basel-Landschaft, Switzerland; 2 Lino Biotech AG, Soodstrasse 52, 8134 Adliswil, Zurich, Switzerlandvolkerga@miltenyi.com (V.G.)

**Keywords:** focal molography, surface plasmon resonance, bio-layer interferometry, biomolecular affinity, kinetic affinity measurement, antibody–antigen interaction, receptor–ligand interaction, complex matrices, quantification of biomarkers, non-specific binding

## Abstract

Characterizing biomolecular receptor–ligand interactions is critical for research and development. However, performing analyses in complex, biologically relevant matrices, such as serum, remains challenging due to non-specific binding that often impairs measurements. Here, we evaluated Focal Molography (FM) for determining *K*_D_ and kinetic constants in comparison to gold-standard methods using single-domain heavy-chain antibodies in various systems. FM provided kinetic constants highly comparable to SPR and BLI in standard buffers containing blocking proteins, with *K*_D_s of soluble CD4 (sCD4) interactions within a 2.4-fold range across technologies. In buffers lacking blocking proteins, FM demonstrated greater robustness against non-specific binding and rebinding effects. In serum, FM exhibited stable baseline signals, unlike SPR and BLI, and yielded *K*_D_s of sCD4 interaction in 50% Bovine Serum within a 1.8-fold range of those obtained in standard buffers. For challenging molecules prone to non-specific binding (Granzyme B), FM successfully determined kinetic constants without external referencing. Finally, FM enabled direct analyte quantification in complex matrices. sCD4 quantification in cell culture media and 50% FBS showed recovery rates of 97.8–100.3% with an inter-assay CV below 1.3%. This study demonstrates the high potential of FM for kinetic affinity determination and biomarker quantification in complex matrices, enabling reliable measurements under biologically relevant conditions.

## 1. Introduction

The binding interactions of biomolecules are central to a wide range of cellular processes, serving as the basis for cellular signaling, structural organization, enzymatic activity, and immune responses. Consequently, the quantitative analysis of these interactions is essential for the understanding of molecular mechanisms and drug action [1,2,3].

The binding interaction between two biomolecules—whether they are nucleic acids, proteins, or small molecules—can be described by the dissociation equilibrium constant (*K*_D_), a measure of the affinity of non-covalent binding. *K*_D_ corresponds to the analyte concentration where 50% of the ligand is bound by the analyte, with lower *K*_D_ values indicating a higher affinity of the interaction [4]. Importantly, *K*_D_ provides insight into binding affinities only in equilibrium. Some interactions also require very long times to reach equilibrium, especially at low concentrations [5]. This can cause practical challenges due to factors such as the instabilities of proteins over extended time periods. Several methods are available to measure *K*_D_ under equilibrium conditions. These include, among others, isothermal titration calorimetry (ITC) and fluorescence anisotropy utilizing analyte-titration-based approaches [6].

While these equilibrium methods are valuable, they provide no information about the kinetics of the interaction—how the ligand (L) and analyte (A) associate to form the complex (LA) at a rate constant *k*_on_, or how the complex dissociates back into L and A at a rate constant *k*_off_. According to the law of mass action, these kinetic constants are related to the equilibrium affinity constant by *K*_D_ = *k*_off_/*k*_on_ [7,8]. Information on *k*_on_ and *k*_off_ provides deeper insights into the interaction dynamics, essential for studying enzyme–substrate interactions, receptor engagement on immune cells, and drug–target interactions, which are critical for drug design and discovery [1,9]. For example, increasing *k*_off_ has been shown to enhance the selectivity of drugs for therapeutic targets, such as HER2, by promoting selective binding to cells that upregulate the target [10,11].

Several biophysical methods enable the simultaneous characterization of kinetic and thermodynamic parameters. Gold-standard techniques such as Surface Plasmon Resonance (SPR) and Bio-Layer Interferometry (BLI) are widely used [12,13] and typically involve the immobilization of one binding partner, the ligand, on a sensor surface (BLI [13], SPR [14]) or the labeling of a molecule (FIDA—flow-induced dispersion analysis) [15].

These technologies differ significantly in their underlying physical principles. SPR detects molecular binding events by monitoring changes in reflected light, caused by variations in the refractive index near a thin metal sensor surface, typically gold. In the resonance condition, incident light excites surface plasmons—coherent electron oscillations at the metal–dielectric interface—resulting in energy absorption and a measurable decrease in reflected light intensity. These changes are directly proportional to the binding interactions occurring at the sensor surface [14,16]. In contrast, BLI uses white light interference. It measures the spectral shift in the interference pattern of light waves reflected from a layer of biomolecules immobilized on a biosensor tip and a reference layer inside the sensor. As molecules bind, the optical path length between the bio-layer boundary and the reference layer increases, producing a spectral shift that is directly proportional to the binding event [13].

Typically, these technologies are employed to assess *K*_D_, *k*_on_, and *k*_off_ in *in vitro* buffer systems. However, in a cellular context, biomolecules operate in a highly complex environment, interacting with numerous other biomolecules in intricate biological matrices. A subset of these technologies can be adapted to measure *K*_D_ in biologically derived complex matrices, such as the binding of antibodies to their antigens in human serum (e.g., by FIDA [15]). However, optimizing these systems for reliable measurements in complex matrices is often challenging due to non-specific binding to sensor surfaces (e.g., for BLI or SPR measurements [17]). Additionally, when using classical methods in complex matrices, it is often necessary to precisely match the running buffer with analyte-depleted samples for referencing [18]. In practice, such samples are either not sufficiently available, costly to produce, or not matched well enough to carry out a meaningful measurement.

**Figure 1:**
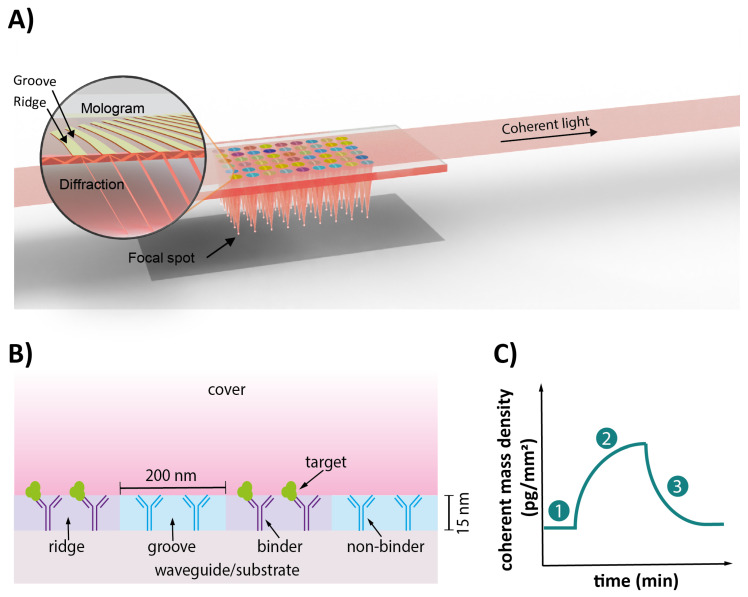
The principle of Focal Molography. (**A**) Schematic of an FM sensor chip. The core sensing element in FM is the mologram, a sub-micron diffraction grating designed with a pattern of alternating regions called ridges and grooves. Coherent light propagates through the waveguide of the sensor chip and is diffracted by the molecules bound in the pattern. This diffraction creates a focal spot, whose intensity is quadratically proportional to the mass difference between ridges and grooves. The mologram is fabricated on a non-fouling polymer layer using reactive immersion lithography. Each chip contains multiple molograms, enabling multiplexing, as each mologram can measure a different target molecule. (**B**) Schematic of the diffraction pattern. At the structural level, the ridges are functionalized with target-specific binders to capture target molecules, while the grooves are backfilled with non-specific binders. This configuration ensures that non-specific binding to both ridges and grooves does not contribute to coherent diffraction, enabling robust signal discrimination. The binding of target molecules to the target-specific binders on the ridges assembles them into the defined diffraction pattern of the mologram, thus generating a coherent signal. The sensing volume in the z-direction corresponds to the depth of the evanescent field, which is approximately 100 nm. (**C**) Schematic of a binding sensorgram. The induced signal is displayed as a change in coherent mass density over time. (1) A stable baseline indicates no significant coherent binding. (2) Upon the addition of the target, signal increases reflect specific binding to the ridges, while (3) decreases correspond to dissociation from the ridges or potential binding to the grooves.

Focal Molography (FM) is an innovative biophysical method offering a novel approach to measuring *K*_D_, *k*_on_, and *k*_off_ in buffer systems but also in meaningful biological matrices, such as blood serum, and even living systems (e.g., cells) [19,20,21,22,23]. The FM sensor chip features a precisely engineered sub-micron pattern of binding sites, known as a mologram. This pattern is designed as a focusing molecular diffraction grating comprising approximately a thousand individual line pairs—referred to as ridges and grooves. The ridges are used to measure interactions of interest, such as those involving a target-specific antibody (Ab), while the grooves serve as internal reference areas, enabling *in situ* subtraction of non-specific binding and other noise sources [20,24]. The principle of FM is illustrated in Figure 1.

In FM, a beam of coherent laser light illuminates the biomolecules on the mologram. This beam is evanescent, meaning it only illuminates the first 100 nanometers above the sensor surface. The core feature of FM is its pre-defined nanopattern, where coherent diffraction occurs from the ridges and grooves of the mologram. This leads to a focused beam that forms a focal spot of constructive interference, whose light intensity is quadratically proportional to the mass difference between ridges and grooves [20,24,25]. The resulting signal, termed “coherent mass density”, increases with specific binding to the ridges [25]. Only the mass difference is detected, as the masses on ridges and grooves are subtracted from each other intrinsically by the wave nature of light.

If molecules adsorb randomly onto the sensor surface rather than binding in an ordered manner on the molographic grating, they scatter light in random directions. This non-directed light does not contribute significantly to the measured signal intensities because it is not focused. This implements an internal referencing mechanism: random non-specific binding to the sensor surface does not produce focused diffraction and, therefore, does not significantly affect the signal [24]. Hence, unlike SPR or BLI, FM leverages diffraction at the molographic pattern, making it particularly effective in complex biological matrices by minimizing interference from non-specific binding events [26,27,28]. To further reduce coherent non-specific binding that might still occur due to directed interactions with the patterned ligand, molecules with biochemical properties similar to the binders on the ridges (e.g., a control Ab that is unspecific to the molecules present in the matrix) are immobilized on the grooves of a mologram. This process, known as “backfilling” [24,25,26], further improves correction for non-specific binding by matching the biochemical properties of the ridges and grooves. As a result, non-specific binding events targeting the ligand are more likely to occur randomly and not coherently [29]. Additionally, each FM sensor chip contains an array of 54 individual molograms, enabling up to 54 parallel measurements on a single chip, significantly enhancing throughput and efficiency.

In this study, we evaluated the performance of FM in characterizing binding interactions and compared it to state-of-the-art technologies such as SPR and BLI. Specifically, we compared the dissociation equilibrium constant (*K*_D_) and kinetic constants (*k*_on_ and *k*_off_) for interactions between single-domain heavy-chain antibodies (V_H_Hs) and their targets, GFP and soluble CD4 (sCD4). To assess its robustness across various matrices, we tested FM in standard buffers as well as complex biological matrices, including serum. Additionally, we investigated FM’s ability to handle problematic analytes prone to non-specific binding, such as Granzyme B. Finally, we demonstrated FM’s utility in analyte quantification by determining the concentration of spiked sCD4 in complex matrices. This study highlights FM’s effectiveness in diverse biosensing applications and its potential to address challenging experimental conditions, including those involving complex biological samples and analytes with high non-specific binding tendencies.

## 2. Materials and Methods

### 2.1. Materials

Buffers and chemicals were purchased from the following sources: phosphate-buffered saline (PBS, pH 7.4) (Cytiva, Marlborough, MA, USA, #BR100672, 10× stock), PBS containing 0.05% Tween 20 (PBST, pH 7.4) (Cytiva, #28-9950-84, 10× stock), Dulbeccos PBS (Merck, Darmstadt, Germany, #D8537), TexMACS^TM^ Medium (T.MACS^TM^) (Miltenyi Biotec B.V. & Co. KG, Bergisch Gladbach, Germany, #130-097-196), Bovine Serum (BS) (Gibco, Thermo Fisher Scientific, Waltham, MA, USA, #16170-078), Fetal Bovine Serum (FBS) (Merck, #F7524), EDTA (Merck, #E7889), protease inhibitor (PI) cOmplete^TM^ Tablets (Merck, #04693116001), NaN_3_ (G-Biosciences, Saint Louis, MO, USA, #786-299), Casein (Merck, #C8654), penicillin–streptomycin (PS) (Gibco, #15140122), EDC (Thermo Fisher Scientific, #22981), sulfo-NHS (Thermo Fisher Scientific, #24525), Ethanolamine (C_2_H_7_NO) (Sartorius AG, Göttingen, Germany, #18-1071), sodium acetate buffer pH 5 (Sartorius, #18-1069), pH 5.5 (Cytiva, #BR100352), pH 6 (Sartorius, #18-1070 )_,_Imidazole (Roth AG, Arlesheim, BL, Switzerland, #3899.3), TCEP (Merck, #646547), N-Ethylmaleimide (Merck, #04259), and Precision Plus Protein Dual Color Standards (Bio-Rad Laboratories Inc., Hercules, CA, USA, #1610374EDU)

PBST+C: PBST with the addition of 0.1% casein. Serum (50% BS and 50% FBS) was diluted in Dulbecco’s PBS and supplemented with 20 mM EDTA, 1× PI for the inhibition of nucleases or proteases, and 0.02% NaN_3_or 100 U/mL PS for the inhibition of bacterial growth. Unless otherwise specified, BS and FBS were supplemented with the first three components. To remove potential aggregates, serum dilutions were centrifuged at 50,000× *g* for 20 min.

Single-domain heavy-chain antibodies (V_H_Hs) with C-terminal Cystein and a His-tag specific for the targets GFP, soluble CD4 (sCD4), CD45, and Granzyme B (GrzB) and a non-target-specific negative control (NC-V_H_H) were provided by Miltenyi Biotec B.V. & Co. KG.

Recombinant proteins were purchased as follows: GFP (Millipore, Burlington, MA, USA #14-392), sCD4 (ACROBiosystems, Beijing, China #LE3-H5228), and GrzB (Sino Biological Inc., Beijing China, #10345-H08H). Maleimide-functionalized ssDNA was purchased from biomers.net GmbH (Ulm, Germany).

### 2.2. Kinetic Comparison Study

Unless otherwise specified, all kinetic experiments were performed at 30 °C using five analyte concentrations. The running buffer for binder immobilization, quality control, and regeneration scouting was either PBS or PBST, which was replaced with the corresponding kinetic buffer prior to kinetic cycles. Bar charts were generated using GraphPad Prism (Version 10.3.1).

### 2.3. Bio-Layer Interferometry (BLI)

BLI experiments were carried out using AR2G sensors (Sartorius, #18-5093) on Octet Red (Sartorius) with Octet Data Acquisition Software (Version 8.2.0.9). Data analysis was performed in Octet Data Analysis Software (Version 8.2.0.7) using a 1:1 binding model. For detailed information, see Supplementary Method S1.

### 2.4. Surface Plasmon Resonance (SPR)

SPR experiments were carried out using CM5 Chips (Cytiva, #29149604) on a Biacore X100 using Biacore X100 Control Software (Version 2.0.1). Data analysis was performed with Biacore X100 Evaluation Software (Version 2.0.1) using a 1:1 interaction model. For detailed information, see Supplementary Method S2.

### 2.5. Focal Molography (FM)

Except where otherwise stated, FM experiments were performed using a single flow cell covering a full chip with 54 individual molograms. The flow cell dimensions were 15 mm × 5.4 mm × 0.1 mm (length × width × height). The dimensions of the flow-cell seal of the single flow cell are shown in Supplementary Figure S24. The size of the molograms was 400 μm × 400 μm (square shape), and the spacing was 690 μm in the flow direction and 750 μm perpendicular to it. The array of molograms was situated in the middle of the flow chamber.

ssDNA-functionalized chips were manufactured by Lino Biotech; the chips are coated with a 2D PEG brushed-copolymer coating published earlier, and the patterning process followed the reactive immersion lithography [20]. On this coating, ssDNA of 20 base pairs in length was covalently attached to the pattern of ridges and grooves, targeting a surface density of around 7 pg/mm^2^ for both ridges and grooves, which should correspond to around 5.6 × 10^8^ binding sites/mm^2^ (assuming a molecular weight of 7.5 kDa for a 20-base-pair ssDNA). This corresponds to roughly 1/35 of the molecule density of a protein monolayer made up of V_H_Hs [30]. The sensor chips were functionalized with different DNA sequences. This approach allows for easy manipulation of the functionalization of either ridges or grooves by DNA-directed immobilization [31] with binders conjugated to the corresponding complementary DNA sequence—indicated by cs01 or cs02. The chip architecture is abbreviated as follows: ridges|grooves.

Except where otherwise stated, regeneration of DNA-directed immobilization was carried out with the following procedure: 30 s of 50 mM NaOH followed by a rinsing phase in running buffer for at least 150 s, both at a flow rate of 400 μL/min.

Binder conjugates for DNA-directed immobilization were prepared by the conjugation of maleimide-functionalized ssDNA strands to V_H_Hs with C-terminal Cystein and a His-tag, and the yield was analyzed via SDS-Page. For detailed information on conjugate preparation and SDS-Page, see Supplementary Methods S3 and S4.

All measurements were carried out on the “Callisto” Series Prototype Reader (Lino Biotech, Adliswil, ZH, Switzerland) in combination with an Alias Autosampler (Spark Holland, Emmen, The Netherlands) and the FM experiment manager software (Developer Version). The molography channel readout wavelength was 785 nm. Additionally, the reader is equipped with a fluorescence readout channel featuring a pixel size of 3.45 μm and an excitation wavelength of 660 nm (not utilized in this work). The instrument setup is shown in Supplementary Figures S22 and S23.

Data analysis was carried out using a proprietary code of Lino Biotech (Developer Version).

#### 2.5.1. Focal Molography: GFP and sCD4 Kinetics

For kinetic experiments involving the analytes GFP and sCD4, replicates were conducted on both s01|s02 and s02|s01 chips to minimize potential artifacts arising from specific DNA sequences. Prior to binder immobilization, quality control (QC) of chips was performed to check for the correct chip architecture and ssDNA surface density. Serial injections of unconjugated ssDNA cs01 and cs02 (usually 400 nM) were conducted under conditions that ensured signal saturation on both ridges and grooves. QC was followed by a regeneration step. Target-specific V_H_H conjugates were immobilized in ridges, followed by the backfilling of grooves with NC-V_H_H conjugates under conditions that ensured signal saturation on ridges and grooves. The immobilization architecture for GFP interactions was αGFP-V_H_H|NC-V_H_H, and for sCD4 interactions, αCD4-V_H_H|NC-V_H_H. Chips were primed in the corresponding kinetic buffer at 1000 μL/min for 1 min. Except for experiments involving serum, chips were primed without data acquisition. Unless otherwise stated, chips were rinsed in the corresponding kinetic buffers for at least 10 min at a flow rate of 300 μL/min to establish a baseline prior to kinetic cycles. For experiments involving 50% BS or 50% FBS, the baseline duration was extended to at least 1 h. For single-cycle kinetics, we used two-minute association at 100 μL/min and two-minute dissociation phases at 300 μL/min, with the final dissociation extended to 10 min, followed by regeneration. Regenerated chips were reused after performing fresh QCs and V_H_H immobilizations for kinetic experiments in different buffers. For the complete workflow, see Supplementary Figure S3. When not used immediately on subsequent days, chips were stored in PBS at 4 °C. Fitting was performed using a 1:1 interaction model. The reported *K*_D_ and kinetic constants represent the median values derived from the analysis of all 54 molograms. It is important to note that these values do not necessarily correspond to those of the mologram with the median signal intensity, which was selected as the representative signal in the figures.

#### 2.5.2. Focal Molography: GrzB Kinetics and GrzB Non-Specific Binding

Analogous to the process described above, the kinetic characterization of the αGrzB/GrzB interaction in T.MACS^TM^ was performed using a single flow cell. The functionalization was αGrzB-V_H_H|αGFP-V_H_H. For single-cycle kinetic experiments, a two-minute association phase at 50 μL/min was followed by three-minute dissociation phases at 250 μL/min. The final dissociation step also lasted three minutes, followed by the regeneration of DNA-directed immobilization.

The non-specific binding of GrzB in FM was qualitatively assessed in an additional experiment using a double flow cell, which divided the chip into two flow cells covering 18 molograms each. The width of each flow cell was 1.55 mm, with the flow-cell seal dimensions provided in Supplementary Figure S24. One area was functionalized with αGrzB-V_H_H|αGFP-V_H_H, while the other area was functionalized with αCD45-V_H_H|αGFP-V_H_H. Subsequently, the functionalized chip was inserted into a single flow chamber, and GrzB binding in T.MACS^TM^ was measured in a single cycle with 10 min association at 15 μL/min and 10 min dissociation at 100 μL/min, with the final dissociation step extended to 40 min, followed by regeneration. Representative molograms correspond to the median coherent mass density signal obtained for each functionalization.

#### 2.5.3. Focal Molography: sCD4 Quantification

The running buffer for sCD4 quantification was PBST, with sCD4 diluted in either T.MACS^TM^ or 50% FBS. Chip QC and functionalization were performed as described above using a single flow cell covering 54 molograms. Regeneration was carried out using 150 mM NaOH. The typical chip architecture was αCD4-V_H_H|NC-V_H_H, except for one replicate in which αCD4-V_H_H was immobilized in the grooves (see Supplementary Figure S20). After chip functionalization, sCD4 standard curves were generated by performing serial injections of five blanks, followed by increasing concentrations of an sCD4 dilution series ranging from 1.56 nM to 200 nM. Each sCD4 concentration was incubated for 10 min at 15 μL/min. Prior to each sCD4 concentration, a baseline step in PBST was conducted for 10 min at 100 μL/min. After generating the standard curve, the chips were regenerated, followed by a second chip QC and a second functionalization. Subsequently, an sCD4 sample with a spiked concentration of 50 nM was injected under the same conditions as described above. Data analysis was carried out as follows. For both the sCD4 standard curve and spiked sCD4 samples, coherent mass signals were normalized to the mean coherent mass signal obtained during the final 50–80% of the preceding baseline. After normalization, the maximum signal obtained during the final 30–70% of sCD4 incubation was calculated. Using these normalized values, four-parameter logistic (4PL) regression standard curves were generated for all 54 molograms. The concentrations of the corresponding spiked samples were then calculated based on the standard curves from the same molograms. For evaluating precision and accuracy, we calculated the coefficients of variation (CVs) and recovery rates. CV, a measure of precision, is defined as the ratio of the standard deviation (SD) to the mean [32]. Recovery, a measure of accuracy, is defined as the ratio of the analyte concentration obtained by the assay to the theoretically spiked concentration [33]. For intra-assay analysis, we calculated the mean value of the 54 obtained sCD4 concentrations from each mologram during a single assay, along with the corresponding SD. For inter-assay analysis, the output concentration of each individual experiment was defined as the mean value of its 54 concentrations. The mean output concentration across different experiments was then calculated, along with the corresponding SD. The analysis included three experiments: two quantifications in T.MACS^TM^ and one quantification in 50% FBS. To evaluate how many molograms are necessary for reliable concentration determination and to explore the potential of multiplexing, we defined sub-groups of the molograms (compare Supplementary Figures S19–S21). An inter-assay analysis was then performed for each sub-group. For the estimation of the Limit of Detection (LoD), the mean concentration of blanks from all experiments and the corresponding SDs were calculated. The LoD was determined using a conventional approach by determining the mean + 3 SD [34]. The LoD was calculated for each matrix individually.

## 3. Results and Discussion

### 3.1. Kinetic Comparison Study

In the first step, we aimed to compare the capability of Focal Molography (FM) to assess *K*_D_, *k*_on_, and *k*_off_. To this end, we validated the values of *K*_D_, *k*_on_, and *k*_off_ obtained using FM against gold-standard methods, specifically two surface-immobilization-based methods: Surface Plasmon Resonance (SPR, Biacore X100) and Bio-Layer Interferometry (BLI, Octet Red Sartorius).

All three techniques allow label-free biomolecular analysis of peptides, proteins, DNA, and small molecules.

BLI is a fluidic-free approach that allows for the parallel measurement of multiple sensors at the same time. Different immobilization strategies based on covalent and affinity-based immobilization are possible, as sensors can be purchased with different pre-functionalizations. Usually, the sensors are single-use.

SPR is a fluidic-based method that is dependent on an external running buffer. As in BLI, multiple sensor chips with different functionalizations for the immobilization of ligands are commercially available. After immobilization, the sensor chip can be regenerated and reused for other measurements with the same ligand.

FM is a fluidic-based method, like SPR. The most common immobilization strategy for FM is via DNA-directed immobilization. For this immobilization strategy, the regeneration is on the DNA level; this means the ligand is regenerated as well. On the one hand, this means that immobilization has to be repeated each time, but on the other hand, the chip can be reused for measurements with different ligands as well, which is a major advantage.

As previously described, FM utilizes an internal reference mechanism based on the nanopattern of ridges and grooves, which makes it less susceptible to non-specific binding. This design provides the practical advantage of eliminating the need for additional reference runs. The best practice for optimizing non-specific binding is to affinity-match ridges and grooves. This is accomplished by backfilling the grooves with a binder that is not specific to the target of interest or any other molecule present in the matrix. It is particularly important to match the isoelectric point (pI) between the binders used on the ridges and grooves, as the pI is the most critical biophysical property influencing the weak, non-specific absorption of molecules [17].

This matching is especially beneficial for measuring challenging proteins, such as highly positively charged proteins. By ensuring that non-specific binding to the ridges and grooves—such as interactions with the DNA used during immobilization—is balanced, any mass difference between the ridges and grooves is minimized, enabling accurate measurements. Conventionally, the functionalization of ridges and grooves is represented using a vertical bar notation (e.g., Target-binder|Control-binder).

#### 3.1.1. Workflow

*K*_D_ and kinetic constants were determined for two model systems using single-domain heavy-chain antibodies (V_H_Hs), specifically αGFP and αCD4 (both with a C-terminal His-tag followed by a Cys residue), and their respective target antigens, GFP and soluble CD4 (sCD4, extracellular domain: K26-W390).

**Figure 2:**
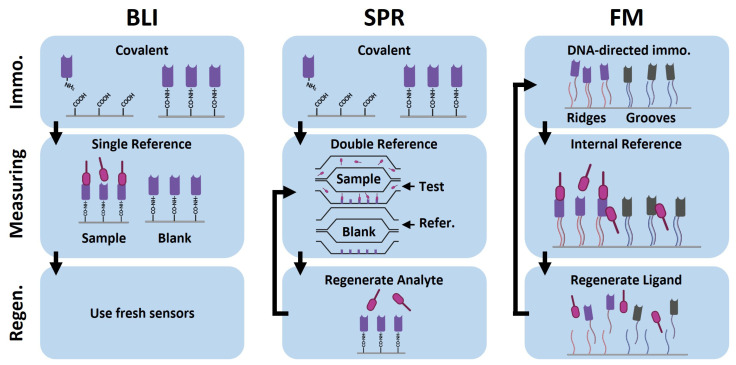
A schematic representation of the workflow for kinetic measurements on the surface-based technologies Surface Plasmon Resonance (SPR), Bio-Layer Interferometry (BLI), and Focal Molography (FM). (1) Immobilization: In BLI and SPR, immobilization involves covalently binding the target-specific single-domain heavy-chain antibody (V_H_H) ligands onto EDC/NHS-activated carboxyl groups on sensor surfaces via amine coupling. In contrast, FM employs DNA-directed immobilization, where the surface’s ridges and grooves are functionalized with specific ssDNA strands. V_H_Hs conjugated to complementary DNA sequences hybridize with these strands, providing a flexible immobilization method for ridge and groove functionalization. In our workflow, ridges were functionalized with target-specific V_H_Hs, and grooves were backfilled with non-target-specific control V_H_Hs. (2) Measuring and referencing: SPR uses a double flow-cell design for measurement and referencing. One flow cell is functionalized with target-specific V_H_Hs, while the other remains non-functionalized (reference). For SPR, single-cycle kinetics were measured, and referencing was carried out following a double-referencing approach. In this approach, signals are referenced to both the reference cell—correcting for non-specific binding events—and a blank reference run—correcting for measurement drift. Therefore, the reference approach in SPR should correct for both non-specific binding events and measurement drift. The drawback is the requirement of an additional buffer run for referencing. BLI performs parallel measurements across multiple sensor tips. Kinetic runs and reference runs are measured simultaneously, with single referencing achieved by subtracting signals from a functionalized sensor tip measuring a blank sample. This approach allows for a higher throughput, but referencing is limited to correcting for measurement drift. In FM, single-cycle kinetics were measured using a single flow cell covering all 54 molograms. The unique architecture of ridges and backfilled grooves provides an internal reference, therefore simplifying measurements by eliminating additional referencing runs. This simplified referencing approach, combined with the array of 54 molograms on a single chip, holds great potential for the high-throughput screening of binding interactions. In this study, all 54 molograms were used to measure the same interaction, and the median *K*_D_ and kinetic constants were calculated for comparison to SPR and BLI. (3) Regeneration: SPR chips are regenerated after each run to remove analytes and allow chip reuse. However, regeneration conditions must be developed and tailored to each ligand–analyte pair, which limits throughput. BLI uses cheaper disposable sensors, making it more practical for rapid testing. FM enables regeneration at the DNA level, allowing the re-immobilization of fresh V_H_H conjugates without additional regeneration scouting. Illustrations were created with BioRender.com.

The workflow of our kinetic comparison study is summarized in Figure 2. It comprises three stages: (1) immobilization, (2) measurement and referencing, and (3) regeneration. Each stage is implemented differently across the three technologies, SPR, BLI, and FM, due to a distinct device design, each with its specific advantages and practical limitations. For this study, we adopted the most common approaches for each technology; however, alternative experimental strategies are also feasible [35,36]. For SPR and BLI, non-oriented immobilization was performed via carboxyl-modified surfaces [14,37], while DNA-directed immobilization was applied for FM [31]. Double referencing was used for SPR [14,37], while single referencing was employed for BLI [37].

#### 3.1.2. Kinetic Constants Across Different Matrices Demonstrate High Cross-Method Comparability

First, we validated the ability of FM to assess *K*_D_ and the kinetic constants *k*_on_ and *k*_off_ in comparison to the gold-standard methods BLI and SPR. Additionally, we investigated the impact of buffer changes on the measurement performance. All reported *K*_D_s and kinetic constants represent the means of replicates ± one standard deviation (SD) (for more details, see Supplementary Figures S6–S11).

**Figure 3:**
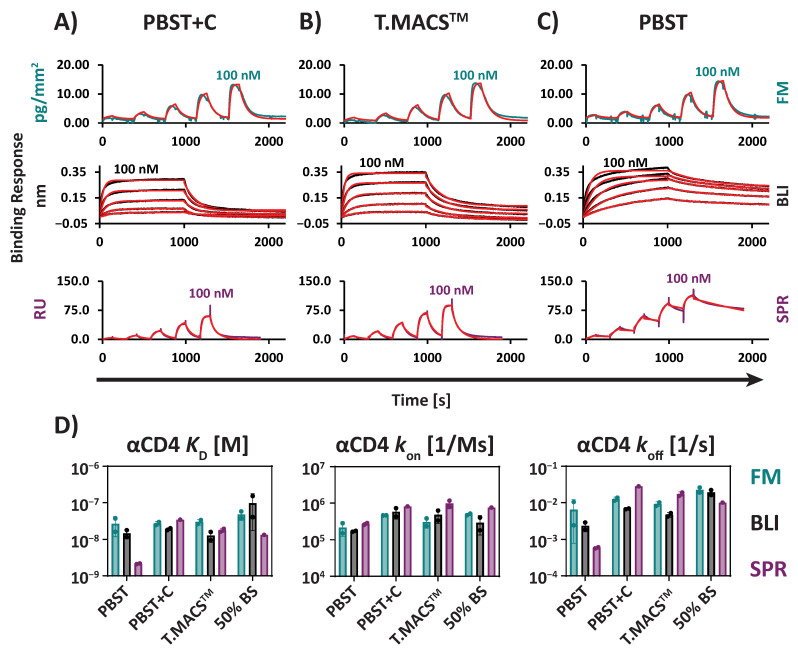
αCD4/sCD4 interaction measured by FM, BLI, and SPR. Representative sensorgrams and kinetic fits (red) are shown for (**A**) a buffer with a blocking protein (PBST+C), (**B**) T.MACS^TM^ cell culture medium, and (**C**) a buffer without blocking proteins (PBST). (**D**) Comparison of *K*_D_ and the kinetic constants *k*_on_ and *k*_off_ across the different technologies in different matrices (50% BS = 50% Bovine Serum). Columns represent the means of replicates with error bars of one SD. Dots represent individual replicates. All fitting models were based on a 1:1 Langmuir fit. Partial fitting was applied for BLI due to the observation of biphasic dissociation. A decrease in the dissociation rate *k*_off_ was observed for all three technologies in the absence of blocking proteins, with the most pronounced effect seen in SPR. All sCD4 dilution series ranged from 2.6 to 100 nM.

Kinetic characterization of the αCD4/sCD4 interaction revealed that SPR, BLI, and FM produced very similar *K*_D_ and kinetic constants across typical matrices used for interaction analysis. These included surfactants and blocking proteins such as casein in PBST+C (Figure 3A,D, Table 1), with the observed *K*_D_s within 1.9-fold of each other [FM: 27 nM ± 3.7 nM; BLI: 19 nM ± 1.7 nM; SPR: 35 nM (n = 1)]. Similarly, in TexMACS^TM^Medium (T.MACS^TM^), which contains HSA as a blocking protein (Figure 3B,D, Table 1), the observed *K*_D_s were within the 2.4-fold range [FM: 30 nM ± 5.4 nM; BLI: 13 nM ± 4.5 nM; SPR: 18 nM ± 2.4 nM]. These results demonstrate excellent cross-comparability between the three technologies.

To assess the stability of the *K*_D_ measurement in buffer systems, which are more challenging due to missing blocking proteins, we determined *K*_D_ and kinetic constants in PBST (Figure 3C,D, Table 1). In this more challenging matrix, a higher variation between *K*_D_ and kinetic constants obtained with the different technologies was determined, with *K*_D_s within 12.5-fold of each other [FM: 27 nM ± 15 nM; BLI: 15 nM ± 4.5 nM; SPR: 2.2 nM ± 0.070 nM].

These differences are mainly attributable to variations in the observed *k*_off_. In buffers lacking blocking proteins, *k*_off_ was slower compared to buffers containing blocking proteins. This observation suggests the presence of either rebinding effects or non-specific binding of sCD4 to the biophysical sensors in buffers without blocking agents [17].

This effect was most pronounced for SPR, where the *k*_off_ values in the different matrices varied by more than 47.9-fold from each other [PBST: 5.9 × 10^−4^ s^−1^± 3.2 × 10^−5^ s^−1^; PBST+C: 2.8 × 10^−2^ s^−1^ (n = 1); T.MACS^TM^: 1.7 × 10^−2^ s^−1^± 2.6 × 10^−3^ s^−1^]. For BLI, *k*_off_ values were within 2.9-fold of each other [PBST: 2.4 × 10^−3^ s^−1^± 7.5 × 10^−4^ s^−1^; PBST+C: 6.8 × 10^−3^ s^−1^± 2.6 × 10^−4^ s^−1^; T.MACS^TM^: 4.7 × 10^−3^ s^−1^± 6.9 × 10^−4^ s^−1^]. Furthermore, we suggest that the effect is underestimated for the BLI measurement, since we had to apply partial fitting models for the αCD4/sCD4 interaction due to biphasic dissociation [38] (Supplementary Figure S5). Finally, FM showed the lowest variation between matrices, with *k*_off_ values within 2.0-fold of each other [PBST: 6.5 × 10^−3^ s^−1^± 5.7 × 10^−3^ s^−1^; PBST+C: 1.3 × 10^−2^ s^−1^± 1.8 × 10^−3^ s^−1^; T.MACS^TM^: 9.2 × 10^−3^ s^−1^± 1.4 × 10^−3^ s^−1^].

**Figure 4:**
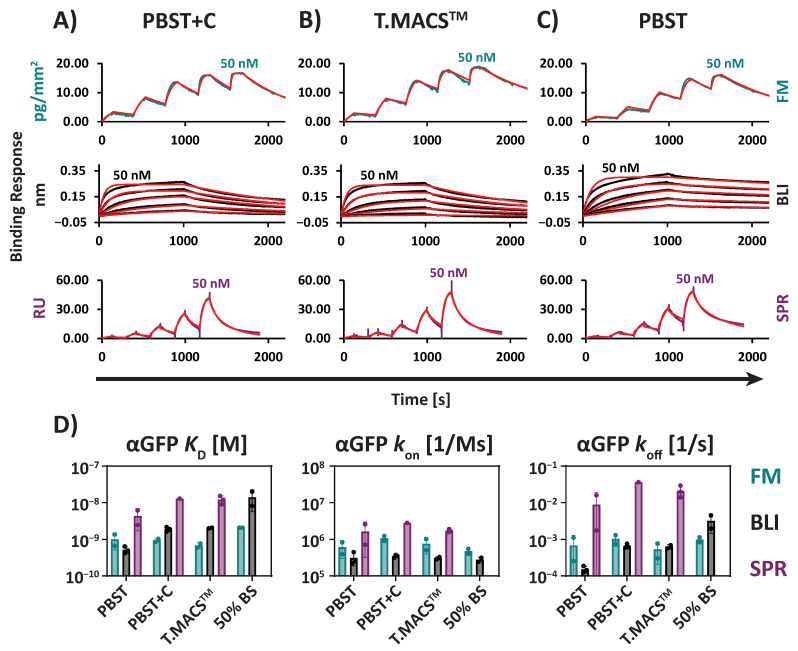
αGFP/GFP interaction measured by FM, BLI, and SPR. Representative sensorgrams and kinetic fits (red) are shown for (**A**) a buffer with a blocking protein (PBST+C), (**B**) T.MACS^TM^ cell culture medium, and (**C**) a buffer without blocking proteins (PBST). (**D**) Comparison of *K*_D_ and the kinetic constants *k*_on_ and *k*_off_ across the different technologies in different matrices (50% BS = 50% Bovine Serum). Columns represent the means of replicates with error bars of one SD. Dots represent individual replicates. All fitting models were based on a 1:1 Langmuir fit. All GFP dilution series ranged from 1.3 to 50 nM.

Compared to the αCD4/sCD4 interaction, the αGFP/GFP interaction exhibited higher variability across SPR, BLI, and FM in all tested matrices (Figure 4A–D, Table 2). In buffers containing blocking proteins, FM [e.g., T.MACS^TM^: 0.68 nM ± 0.12 nM] and BLI [e.g., T.MACS^TM^: 2.0 nM ± 0.071 nM] were again highly comparable, with *K*_D_s within a 3.1-fold range. However, when including SPR [e.g., T.MACS^TM^:12 nM ± 3.2 nM], the *K*_D_s varied within a 17.8-fold range.

This difference is primarily attributed to the generally higher *k*_off_ values observed with SPR [e.g., T.MACS^TM^: 2.1 × 10^−2^ s^−1^± 7.8 × 10^−3^ s^−1^] compared to BLI [e.g., T.MACS^TM^: 6.3 × 10^−4^ s^−1^± 8.9 × 10^−5^ s^−1^] and FM [e.g., T.MACS^TM^: 5.3 × 10^−4^ s^−1^± 3.3 × 10^−4^ s^−1^].

Although less pronounced than for αCD4/sCD4, the effect of lower observable *k*_off_ in buffers without blocking proteins was also evident for the αGFP/GFP interaction (Figure 4C,D, Table 2). This effect was again more pronounced for SPR and BLI. SPR values varied as follows [PBST: 8.8 × 10^−3^ s^−1^± 1.0 × 10^−2^ s^−1^; PBST+C: 3.6 × 10^−2^ s^−1^ (n = 1); T.MACS^TM^: 2.1 × 10^−2^ s^−1^± 7.8 × 10^−3^ s^−1^], with observed values spanning a 4.2-fold range.

For BLI, *k*_off_ values showed a similar trend [PBST: 1.5 × 10^−4^ s^−1^± 3.2 × 10^−5^ s^−1^; PBST+C: 6.6 × 10^−4^ s^−1^± 9.1 × 10^−5^ s^−1^; T.MACS^TM^: 6.3 × 10^−4^ s^−1^± 8.9 × 10^−5^ s^−1^], spanning a 4.4-fold range.

FM exhibited the lowest variation across matrices, with *k*_off_ values within a 2.0-fold range [PBST: 6.8 × 10^−4^ s^−1^± 6.0 × 10^−4^ s^−1^; PBST+C: 1.0 × 10^−3^ s^−1^± 3.9 × 10^−4^ s^−1^; T.MACS^TM^: 5.3 × 10^−4^ s^−1^± 3.3 × 10^−4^ s^−1^].

Furthermore, for the αGFP/GFP interaction, the mean *k*_off_ values for SPR and FM exhibited relatively high standard deviations between replicates in buffers without blocking proteins. This suggests that excluding blocking proteins can potentially introduce measurement artifacts and may reduce the reproducibility of surface-based protein interaction experiments.

**Figure 5:**
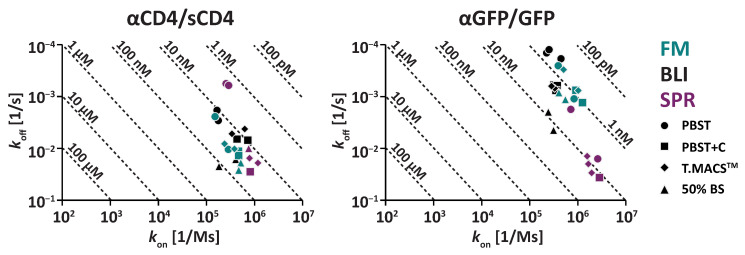
Iso-affinity plot of the αCD4/sCD4 and αGFP/GFP interactions measured by FM, BLI, and SPR in PBST, PBST+C, T.MACS^TM^, and 50% Bovine Serum (BS). The plot shows the relationship between the association rate constant (*k*_on_, x-axis) and the dissociation rate constant (*k*_off_, y-axis) for all replicates. Diagonal lines indicate lines of equal affinity (*K*_D_).

In summary, our experiments demonstrated very good comparability between the *K*_D_ and kinetic constants obtained with FM, SPR, and BLI (data summarized in Figure 5), particularly in standard matrices containing surfactants and blocking proteins (PBST+C). This comparability extended to more challenging matrices, such as cell culture media (T.MACS^TM^). The determination of binding directly in cell culture media or supernatant is particularly valuable for assessing binding directly in the media used for cellular experiments or directly measuring the binding of secreted proteins without purification. Overall, BLI and SPR showed a more pronounced impact when removing blocking proteins on *k*_off_ compared to FM, which has an integrated correction for non-specific binding. This suggests that non-specific binding of sCD4 or GFP to the sensor surface results in a decrease in apparent *k*_off_, as is particularly evident for the sCD4 interaction measured by SPR and the biphasic dissociation phase observed in BLI. In conclusion, our experiments verified that FM provides *K*_D_, *k*_on_, and *k*_off_ values comparable to gold-standard methods and is more robust to non-specific binding, making it a reliable and versatile tool for surface-based protein interaction analysis.

#### 3.1.3. Internal Referencing Enables *K*_D_, *k*_on_, and *k*_off_ Determination in Serum

In the second step, we aimed to evaluate the ability of FM to determine *K*_D_, *k*_on_, and *k*_off_ in complex biological matrices. Blood serum represents a particularly challenging matrix for affinity assessment due to its complex mixture of proteins and small molecules. Serum is highly viscous and exhibits a high refractive index, which can lead to discontinuities or drifts in the sensorgrams of kinetic measurements [4]. Additionally, the serum composition varies depending on the subject and their health status [39]. Nonetheless, biological matrices like serum are of great interest for understanding binding parameters, as they better reflect the environments in which biological processes occur [40].

To evaluate performance in serum, we used 50% Bovine Serum (BS) and 50% Fetal Bovine Serum (FBS) as matrices. For each method in this kinetic study, we included a baseline incubation step using the corresponding blanked serum prior to analyte introduction to assess the influence of non-specific binding on the signal. FM demonstrated a stable baseline signal for both BS and FBS, whereas BLI and SPR exhibited substantial baseline drift, even after 1 h of serum incubation (Supplementary Figures S12–S14). Given the high total protein concentration in serum compared to standard laboratory buffers, we attribute this baseline drift to the non-specific binding of serum proteins to the sensor surfaces in BLI and SPR [17].

**Figure 6:**
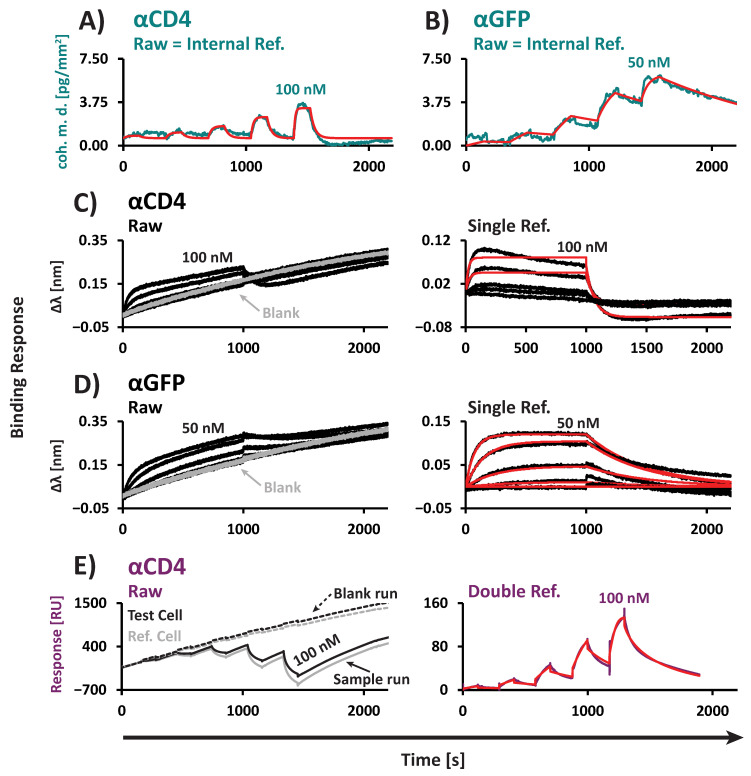
Interactions measured in 50% Bovine Serum (BS). Representative sensorgrams and kinetic fits (red) for the αGFP/GFP and αCD4/sCD4 interactions. (**A**) FM: αCD4/sCD4. (**B**) FM: αGFP/GFP. (**C**) BLI: αCD4/sCD4. (**D**) BLI: αGFP/GFP. (**E**) SPR: αCD4/sCD4. (C-E) Left: Unreferenced data. Right: Reference-subtracted data with corresponding fits. (**A**,**B**) For FM, due to its internal referencing strategy using ridges and grooves, no additional referencing of the raw data was necessary, and no baseline drift was observed for either interaction. (**C**,**D**) For BLI, substantial drift was observed for both interactions, as demonstrated by the drift in the blank. The drift could be corrected by single referencing to the blank reference for both interactions. (**C**) Due to the signal reduction in 50% BS, only the two highest sCD4 concentrations were included for fitting. (**E**) For SPR, substantial drift was observed for both test and reference cells, as indicated by the blank drift. The injection of sCD4 resulted in strong negative signals in both the test and reference cells. The drift and negative signals of the sensorgrams were corrected by double referencing to the reference cell and the blank reference run. All sCD4 dilution series ranged from 2.6 to 100 nM. All GFP dilution series ranged from 1.3 to 50 nM.

Thanks to the nanopattern of ridges and backfilled grooves, FM provided raw data suitable for direct kinetic evaluation without requiring external referencing or baseline drift correction (Figure 6A,B). While the signals for both the sCD4 (Figure 6A) and GFP (Figure 6B) interactions were dampened in 50% BS compared to standard buffers containing blocking agents (PBST+C, Figure 3A and Figure 4A), the *K*_D_ and kinetic constants remained highly comparable across the two conditions (Figure 3D and Figure 4D, Table 1 and Table 2). For the sCD4 interaction, *K*_D_ values were within a 1.8-fold range [50% BS: 48 nM ± 14 nM vs. PBST+C: 27 nM ± 3.7 nM]. Similarly, for the GFP interaction, *K*_D_ values were within a 2.3-fold range [50% BS: 2.1 nM ± 0.0061 nM vs. PBST+C: 0.94 nM ± 0.14 nM].

Although substantial baseline drift was observed with BLI, reference subtraction using blanked serum enabled the kinetic evaluation of both interactions (Figure 6C,D). Similar to FM, signal reduction was observed in 50% BS compared to PBST+C (Figure 3A and Figure 4A). *K*_D_s and kinetic constants varied slightly between 50% BS and PBST+C (Figure 3D and Figure 4D, Table 1 and Table 2). For the sCD4 interaction, *K*_D_ values were within a 5.1-fold range [50% BS: 97 nM ± 79 nM vs. PBST+C: 19 nM ± 1.7 nM]. Similarly, for the GFP interaction, *K*_D_ values were within a 7.4-fold range [50% BS: 14 nM ± 8.5 nM vs. PBST+C: 1.9 nM ± 0.27 nM].

Notably, SPR exhibited strong negative signals during the injection of sCD4 (Figure 6E). This effect was observed in both the test and reference channels, while positive signals occurred during sCD4 dissociation. Interpreting this behavior is challenging, but a potential explanation could be the displacement of serum proteins during sCD4 injection. Despite this, double referencing—by subtracting the reference cell signal and then the blanked serum signal—produced data suitable for kinetic evaluation. The referenced SPR data showed a comparable signal intensity in 50% BS to that in PBST+C (Figure 3A). *K*_D_ and kinetic constants were in a similar range, with *K*_D_s differing by a 2.7-fold factor [50% BS: 13 nM (n = 1) vs. PBST+C: 35 nM (n = 1)]. However, as the negative signal upon sCD4 injection is not fully understood, the SPR results in 50% BS should be interpreted with caution. Additionally, despite optimization efforts, we were unable to perform a kinetic evaluation of the GFP interaction in 50% BS using SPR due to highly unstable baselines (Supplementary Figures S10D and S13).

In summary, FM enables the assessment of *K*_D_, *k*_on_, and *k*_off_ in highly complex biological matrices such as serum. For the tested interactions (GFP and sCD4 with the corresponding V_H_H binders), the binding parameters determined in serum were found to be highly comparable to those obtained in standard buffers. However, it is important to note that this observation may not be generalizable to all ligand–analyte interactions, particularly due to the high affinity of the V_H_Hs used in this study. Differences in binding constants between matrices may arise for other molecules due to matrix effects, such as particular serum proteins binding to the analyte or ligand, which could directly influence the interaction or effective concentration of the analyte. Importantly, we were able to verify the FM results using alternative approaches. However, SPR and BLI highlighted the need for extensive normalization and optimization, particularly in complex matrices like serum. These methods are heavily reliant on the availability of serum that is precisely depleted of the analyte for normalization—a requirement that is difficult, if not impossible, to meet in practice. This underscores the practical advantages of FM for binding studies in complex biological environments.

### 3.2. Characterization of Challenging Molecules Exhibiting Strong Non-Specific Binding

After validating FM for measuring protein–protein interactions in standard laboratory buffers and serum, we further investigated its ability to assess kinetic binding parameters for challenging molecules prone to non-specific binding to surfaces, such as Granzyme B (GrzB).

**Figure 7:**
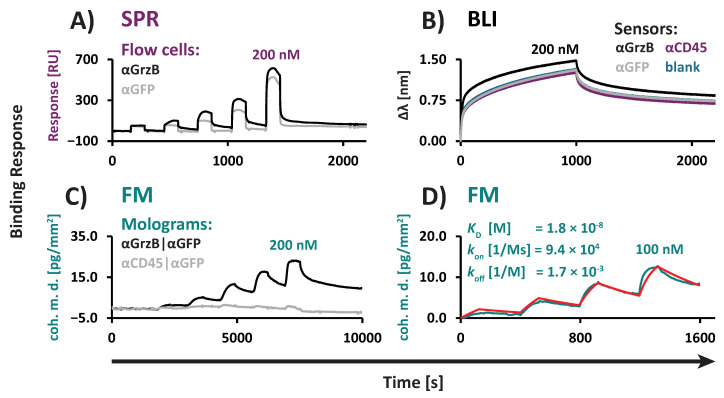
Characterization of binding interactions in T.MACS^TM^ cell medium involving the challenging target Granzyme B, prone to non-specific binding to surfaces, using SPR, BLI, and FM. (**A**) Non-specific binding in SPR: The control flow cell was biochemically matched to the αGrzB test cell by the immobilization of a control V_H_H (αGFP). The non-specific binding of GrzB to the control cell is evident, as demonstrated by the signal curvature during the injection of GrzB and the increase in baseline. Instant signal spikes are induced by SPR bulk effects and do not represent non-specific binding. The GrzB dilution series ranged from 12.5 to 200 nM. (**B**) Non-specific binding in BLI: Control sensors were biochemically matched to the αGrzB sensor by immobilizing control V_H_Hs (αGFP, αCD45). A non-functionalized blank control sensor was also included. The non-specific binding of GrzB was observed for all control sensors, including both blank and biochemically matched sensors, as demonstrated by the Δλ signal increase during the injection of 200 nM GrzB. (**C**) No non-specific binding in FM: Test and control molograms were individually functionalized using a double flow cell. Test mologram ridges were functionalized with αGrzB-V_H_H, and grooves were biochemically matched by backfilling with αGFP-V_H_H (αGrzB|αGFP). Negative control molograms were functionalized with αCD45-V_H_H and backfilled with αGFP-V_H_H (αCD45|αGFP). GrzB was injected in parallel in test and control molograms using a single flow cell. Specific signals were observed in test molograms, while no non-specific binding signals were detected in control molograms. This confirmed that the signal originates from the specific αGrzB/GrzB interaction, rather than the non-specific binding of GrzB to the chip surface. The GrzB dilution series ranged from 12.5 to 200 nM. (**D**) A representative sensorgram and kinetic fit (red) of FM kinetic evaluation: Kinetic evaluation of the αGrzB/GrzB interaction was conducted in a single flow cell containing 54 αGrzB|αGFP molograms in T.MACS^TM^. Kinetic parameters represent median values obtained from the array of 54 molograms. The GrzB dilution series ranged from 12.5 to 100 nM.

GrzB is a potent serine protease predominantly secreted by cytotoxic T cells and natural killer (NK) cells, sufficient for inducing apoptosis in target cells [41,42]. Structurally, GrzB bears a high number of basic amino acids, enabling it to bind to cell surfaces primarily through charge–charge interactions [43,44]. This property complicates biophysical characterization, as positively charged molecules like GrzB have a tendency for non-specific binding to negatively charged surfaces such as carboxymethyl dextran-based sensors [45]. The GrzB interaction was characterized using three technologies: SPR, BLI, and FM (Figure 7).

To reduce non-specific binding, we used a buffer containing high concentrations of blocking proteins (T.MACS^TM^) and biochemically matched our control sensors (BLI), reference flow cells (SPR), or grooves (FM) by immobilizing GrzB non-specific V_H_Hs to achieve a similar density to the αGrzB-V_H_Hs on our test sensors (BLI), test flow cells (SPR), or ridges (FM). For FM, we further included control molograms where both ridges and grooves were immobilized with GrzB non-specific V_H_Hs (Supplementary Figure S15).

Despite the optimization and referencing strategies applied, we observed strong non-specific binding of GrzB in both SPR (Figure 7A) and BLI (Figure 7B), making reliable kinetic analysis with these systems unfeasible (Supplementary Figures S16 and S17). For SPR, even after double referencing to correct for drift and non-specific binding signals, kinetic analysis remained infeasible, with kinetic constants falling outside of the instrument’s specifications (Supplementary Figure S16). For BLI, after single referencing to the blank, the measured kinetic constants from test and control sensors were almost identical, confirming that the observed kinetics arose from the non-specific binding of GrzB to the BLI sensor surface rather than specific binding to αGrzB-V_H_H (Supplementary Figure S17).

Using FM, no non-specific binding was observed on the control molograms (Figure 7C). This allowed us to quantify the kinetic parameters of the αGrzB/GrzB interaction in T.MACS^TM^ without the need for external referencing (Figure 7D). The *K*_D_ obtained in T.MACS^TM^ was 18.3 nM.

In summary, our results exemplify the advantages of FM over SPR and BLI in assessing affinities of biomolecules with high intrinsic non-specific binding to surfaces. The internal referencing approach integrated into the mologram enables the effective correction of even strong non-specific binding to surfaces or proteins, providing robust and reliable kinetic analysis.

### 3.3. sCD4 Quantification and Multiplexing Capabilities

Finally, in the fourth step, we investigated the utility of FM for the quantification of analytes in complex matrices. As a model system for this assay, we used the αCD4/sCD4 interaction.

**Figure 8:**
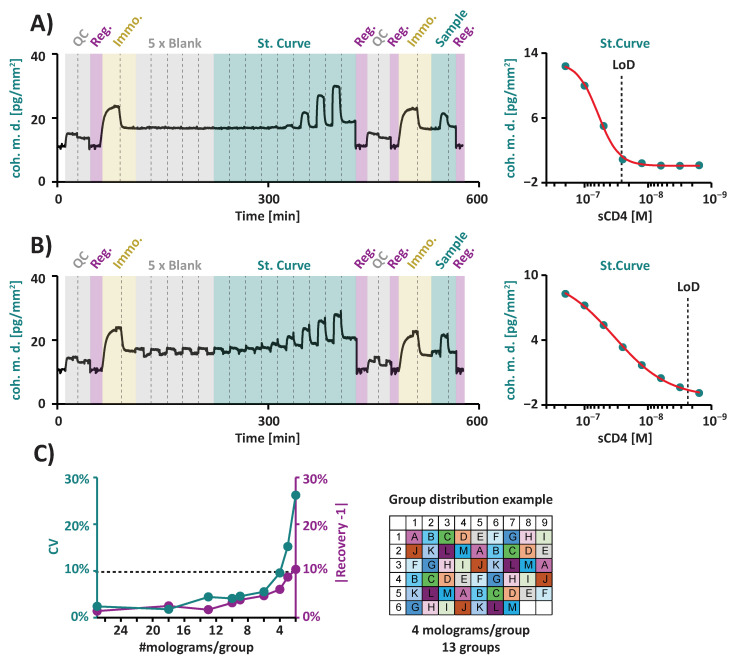
Using FM for the quantification of sCD4. Representative sensorgrams and fitted standard curves (red) with corresponding LoDs for (**A**) sCD4 samples in T.MACS^TM^ cell medium vs. (**B**) sCD4 samples in 50% FBS. (**A**,**B**) Quantification assays were performed on 54 molograms in parallel in PBST running buffer, following this sequence: First quality control (QC): Serial injections (e.g., cs01 and cs02) of ssDNA complementary to the ssDNA functionalized on ridges (s01) and grooves (s02) were conducted to verify the correct mologram architecture and ssDNA surface density, followed by regeneration. First immobilization of V_H_H conjugates: Target-specific conjugates (here: αCD4-cs01) were immobilized on the ridges, and target unspecific conjugates (here: NC-V_H_H-cs02) were used for backfilling the grooves. Blank measurements and standard curve: After five blank injections, increasing concentrations of sCD4 were injected to generate a standard curve, followed by regeneration. The sCD4 dilution series ranged from 1.6 to 200 nM. Each concentration was preceded by a baseline measurement in PBST, which was used to calculate the Δ “coherent mass density” for each sCD4 concentration. Second QC: A second QC was conducted, analogous to the first QC. Second immobilization: A second immobilization of V_H_H conjugates was performed, analogous to the first immobilization. Injection of a spiked sCD4 sample: A 50 nM sCD4 sample was injected under conditions identical to those used during the blank measurements and standard curve generation, followed by regeneration. (**C**) Multiplex analysis using the sCD4 quantification data. Mologram positions were assigned to different groups, representing individual analytes. Group positions were distributed in checkerboard-like patterns across the array of 54 molograms (example pattern shown here: 13 groups, each with 4 molograms). Inter-assay analysis was carried out for each group, and plotted values correspond to groups with the lowest performance.

For this analysis, sCD4 quantification experiments were performed both in T.MACS^TM^ (Figure 8A) and 50% FBS (Figure 8B) to serve as examples of biologically relevant matrices. Standard curves were generated on an αCD4 functionalized FM chip with sCD4 concentrations ranging from 1.56 to 200 nM. Subsequently, the chip was regenerated, and spiked sCD4 at a concentration of 50 nM was measured on the same sensor using a fresh αCD4 functionalization. The concentration of the spiked sCD4 was calculated for each of the 54 molograms on the chip using its corresponding standard curve.

The evaluation of sCD4 quantification in T.MACS^TM^ cell culture medium (Figure 8A, Table 3, individual replicates Supplementary Figure S18) using two individual sensor chips resulted in excellent mean recovery rates of 100.3% and 97.8%. The coefficients of variation (CV) across the 54 molograms on each chip (intra-assay CV) were below 5% and below 14%, respectively. Preliminary LoD estimation in T.MACS^TM^ was 14.7 nM. We next assessed the quantification of sCD4 in 50% FBS (Figure 8B, Table 3). Remarkably, sCD4 quantification in 50% serum maintained excellent performance, with a recovery rate of 99.0%, an intra-assay CV of 13.0%, and an LoD estimate of 2.6 nM. Inter-assay analysis was performed by taking into consideration the mean concentrations from all molograms per sensor chip, across all chips and matrices (Table 3). This analysis demonstrated a CV below 1.3% between the different sensors and a recovery of 99.1%, indicating high inter-assay reproducibility.

To explore the potential of using the 54 molograms on a single FM chip for multiplexing multiple analytes, we conducted a simulation using the above data with a reduced number of molograms per analyte. Each mologram position was assigned to a specific group, representing an individual analyte. The positions of each group were distributed across the array in a checkerboard-like pattern to ensure that positions for different groups were spread broadly over the array. This approach was carried out for different group sizes, ranging from n = 2 (corresponding to 27 molograms per analyte) to n = 27 (corresponding to 2 molograms per analyte) (Supplementary Figures S19–S21). We found that 4 molograms per group (corresponding to 13 analytes per chip) still resulted in high precision, with inter-assay CVs below 10%, and high accuracy, with recoveries within ±10% of the spiked concentration (Figure 8C). In summary, these results demonstrate that FM can be used for accurate and precise quantification in complex media in a multiplexing setup, enabling simultaneous analysis of multiple analytes with high accuracy.

## 4. Conclusions

In this study, we performed the first verification of Focal Molography (FM) for the determination of *K*_D_, *k*_on_, and *k*_off_, as well as for protein quantification.

Firstly, our data show that FM can reliably assess *K*_D_ and kinetic constants with excellent agreement with gold-standard interaction analysis methods, such as Bio-Layer Interferometry (BLI) and Surface Plasmon Resonance (SPR). This was validated across a wide range of matrices, including both *in vitro* systems (buffer including surfactants and blocking proteins) and complex cell culture media. These results introduce FM as an innovative and reliable method for measuring interaction parameters. Our results in buffers with and without blocking proteins underscore the critical role of blocking agents in minimizing non-specific binding and rebinding effects in surface-based technologies [17], for which non-specific binding to the biosensors is a known problem [46]. Moreover, we demonstrated that the internal referencing mechanism of FM, leveraging the nanopattern of ridges and grooves, effectively mitigates these effects.

Secondly, binding parameters could be determined even in highly complex matrices (50% Bovine Serum), which are inherently challenging for surface-immobilization-based technologies like BLI and SPR due to significant non-specific binding that masks the specific signal [17,24,47]. Using elaborated referencing with serum matched and depleted of the analyte, we demonstrated that *K*_D_, *k*_on_, and *k*_off_could still be assessed using BLI and SPR. However, this approach was heavily reliant on the availability of matched serum [18]. In contrast, FM demonstrated a robust ability to assess *K*_D_, *k*_on_, and *k*_off_ in serum due to the internal referencing mechanism of the mologram. During serum-based measurements, FM maintained stable baseline signals, eliminating the need for external referencing. This was a significant advantage over SPR and BLI, both of which exhibited pronounced baseline drift, likely caused by the non-specific binding of serum proteins to the sensor surface. Furthermore, FM displayed consistent kinetic behavior across serum and buffer conditions, whereas SPR and BLI required extensive reference adjustments for data stability. Notably, SPR even showed signal inversions in raw data during soluble CD4 (sCD4) association, complicating interpretation. Unlike SPR and BLI, FM is less affected by the viscosity or refractive index of the running buffer, reducing the dependency on matching the sample and buffer conditions [4,18,19,23]. Those refractive index changes can easily be caused by small concentration changes in the buffer or temperature effects [4,18]. This flexibility allows for the use of standard serum preparations depleted of the analyte or buffer systems with added proteins (e.g., albumin) to mitigate non-specific binding effects if analyte-depleted matched serum is unavailable to assess *k*_off_.

Thirdly, the internal referencing mechanism of FM theoretically enables the assessment of *K*_D_, *k*_on_, and *k*_off_ for proteins with an intrinsic tendency for non-specific binding [20,24]. By backfilling with proteins that share similar biochemical properties to the specific binders, FM can effectively correct for non-specific binding. This was exemplified by Granzyme B (GrzB), a protein that exhibited non-specific binding in both SPR and BLI, even in the presence of high concentrations of blocking proteins [48]. This renders kinetic evaluation impossible, even with extensive biochemical reference matching. In contrast, FM demonstrated no non-specific binding artifacts under the same conditions, enabling the direct kinetic characterization of GrzB. Using FM, we successfully determined apparent *K*_D_, *k*_on_, and *k*_off_ for GrzB. However, it is important to note a caveat: the accuracy of these parameters may be affected by the loss of GrzB by non-specific binding during sample preparation or measurement (e.g., adsorption to the tubing of the flow system). Such losses effectively reduce the GrzB concentration, leading to measurements at lower concentrations than intended. This limitation can be mitigated by performing repeated measurements at the same concentration to identify potential saturation effects related to non-specific binding.

Fourth, our results demonstrate that FM enables the direct quantification of proteins from complex media, as shown in the sCD4 model. We achieved an excellent recovery of 99.1% and high precision with a CV below 1.3%. This makes FM highly relevant for applications such as rapid and reliable protein production assessment, process optimization, or biomarker research. The applied two-step approach—first generating a standard curve and subsequently measuring an unknown concentration after chip regeneration—suggests that standard curve generation can be decoupled from the sample measurements. This implies that it may be feasible to save a pre-determined standard curve on a sensor chip, eliminating the need to repeatedly perform standard curve measurements during routine experiments. Moreover, even when using a subset of the 54 sensors on the chip (Figure 8), reliable results for multiplexing up to 13 analytes were obtained. This configuration, using four molograms per analyte, resulted in CVs below 10% and recovery rates within ±10% of the spiked concentration. It is noteworthy that these experiments were conducted using sensor chips fabricated with the first-generation lithography process. This suggests that further improvements in recovery, CVs, and multiplexing capabilities may be achieved with commercially manufactured sensor chips based on the advanced image reversal reactive immersion lithography (RIL) process for fabrication [49].

A limitation of our study is that we were only able to investigate a limited number of interactions (three model systems) across various sample matrices. Additional data will be necessary to comprehensively validate FM for both kinetic interaction analysis and concentration measurements. However, the dataset presented here already highlights the significant potential of FM for addressing highly challenging questions in determining biophysical interaction parameters. These include applications in complex biological environments such as body fluids (e.g., serum), cell culture media, and supernatants, as well as for molecules that intrinsically exhibit a high degree of non-specific binding.

## Figures and Tables

**Table 1:** *K*_D_ and kinetic constants of the αCD4/sCD4 interaction. Comparison across the different technologies FM, BLI, and SPR in a buffer without blocking proteins (PBST), in a buffer with a blocking protein (PBST+C), in a cell culture medium (T.MACS^TM^), and in 50% Bovine Serum (50% BS). *K*_D_ and kinetic constants correspond to the means of individual replicates (n) ± one standard deviation (SD).

		FM			BLI			SPR		
**Matrix**	**Parameter**	**Mean**	**± SD**	**n**	**Mean**	**± SD**	**n**	**Mean**	**± SD**	**n**
	*K*_D_ [M]	2.69 × 10^−8^	1.51 × 10^−8^		1.47 × 10^−8^	4.46 × 10^−9^		2.15 × 10^−9^	6.96 × 10^−11^	
PBST	*k*_on_ [1/Ms]	2.18 × 10^5^	9.48 × 10^4^	2	1.71 × 10^5^	7.37 × 10^3^	2	2.73 × 10^5^	2.36 × 10^4^	2
	*k*_off_ [1/s]	6.47 × 10^−3^	5.70 × 10^−3^		2.38 × 10^−3^	7.50 × 10^−4^		5.87 × 10^−4^	3.18 × 10^−5^	
	*K*_D_ [M]	2.73 × 10^−8^	3.75 × 10^−9^		1.91 × 10^−8^	1.71 × 10^−9^		3.46 × 10^−8^	n = 1	
PBST+C	*k*_on_ [1/Ms]	4.66 × 10^5^	1.41 × 10^3^	2	5.81 × 10^5^	2.08 × 10^5^	2	8.13 × 10^5^	n = 1	1
	*k*_off_ [1/s]	1.26 × 10^−2^	1.77 × 10^−3^		6.80 × 10^−3^	2.63 × 10^−4^		2.82 × 10^−2^	n = 1	
	*K*_D_ [M]	3.02 × 10^−8^	5.37 × 10^−9^		1.28 × 10^−8^	4.52 × 10^−9^		1.79 × 10^−8^	2.37 × 10^−9^	
T.MACS^TM^	*k*_on_ [1/Ms]	3.11 × 10^5^	1.03 × 10^5^	2	4.81 × 10^5^	2.02 × 10^5^	2	9.81 × 10^5^	2.75 × 10^5^	2
	*k*_off_ [1/s]	9.20 × 10^−3^	1.42 × 10^−3^		4.71 × 10^−3^	6.93 × 10^−4^		1.73 × 10^−2^	2.60 × 10^−3^	
	*K*_D_ [M]	4.77 × 10^−8^	1.44 × 10^−8^		9.66 × 10^−8^	7.91 × 10^−8^		1.33 × 10^−8^	n = 1	
50% BS	*k*_on_ [1/Ms]	4.92 × 10^5^	3.39 × 10^4^	2	2.94 × 10^5^	1.58 × 10^5^	2	7.55 × 10^5^	n = 1	1
	*k*_off_ [1/s]	2.27 × 10^−2^	5.16 × 10^−3^		1.93 × 10^−2^	4.23 × 10^−3^		1.01 × 10^−2^	n = 1	

**Table 2:** *K*_D_ and kinetic constants of the αGFP/GFP interaction. Comparison across the different technologies FM, BLI, and SPR in a buffer without blocking proteins (PBST), in a buffer with a blocking protein (PBST+C), in a cell culture medium (T.MACS^TM^), and in 50% Bovine Serum (50% BS). *K*_D_ and kinetic constants correspond to the means of individual replicates (n) ± one standard deviation (SD).

		FM			BLI			SPR		
**Matrix**	**Parameter**	**Mean**	**± SD**	**n**	**Mean**	**± SD**	**n**	**Mean**	**± SD**	**n**
	*K*_D_ [M]	1.01 × 10^−9^	4.94 × 10^−10^		5.17 × 10^−10^	1.13 × 10^−10^		4.30 × 10^−9^	2.59 × 10^−9^	
PBST	*k*_on_ [1/Ms]	6.14 × 10^5^	3.14 × 10^5^	2	3.08 × 10^5^	1.22 × 10^5^	3	1.65 × 10^6^	1.33 × 10^6^	2
	*k*_off_ [1/s]	6.80 × 10^−4^	6.02 × 10^−4^		1.52 × 10^−4^	3.17 × 10^−5^		8.82 × 10^−3^	9.99 × 10^−3^	
	*K*_D_ [M]	9.44 × 10^−10^	1.36 × 10^−10^		1.90 × 10^−9^	2.66 × 10^−10^		1.29 × 10^−8^	n = 1	
PBST+C	*k*_on_ [1/Ms]	1.08 × 10^6^	2.43 × 10^5^	2	3.46 × 10^5^	2.86 × 10^4^	3	2.83 × 10^6^	n = 1	1
	*k*_off_ [1/s]	1.03 × 10^−3^	3.92 × 10^−4^		6.57 × 10^−4^	9.08 × 10^−5^		3.64 × 10^−2^	n = 1	
	*K*_D_ [M]	6.83 × 10^−10^	1.21 × 10^−10^		2.05 × 10^−9^	7.14 × 10^−11^		1.21 × 10^−8^	3.17 × 10^−9^	
T.MACS^TM^	*k*_on_ [1/Ms]	7.63 × 10^5^	3.63 × 10^5^	2	3.06 × 10^5^	3.25 × 10^4^	2	1.71 × 10^6^	1.90 × 10^5^	3
	*k*_off_ [1/s]	5.34 × 10^−4^	3.27 × 10^−4^		6.29 × 10^−4^	8.85 × 10^−5^		2.11 × 10^−2^	7.82 × 10^−3^	
	*K*_D_ [M]	2.08 × 10^−9^	6.06 × 10^−12^		1.41 × 10^−8^	8.47 × 10^−9^		N.A.	N.A.	
50% BS	*k*_on_ [1/Ms]	4.77 × 10^5^	1.05 × 10^5^	2	2.78 × 10^5^	4.90 × 10^4^	2	N.A.	N.A.	0
	*k*_off_ [1/s]	9.91 × 10^−4^	2.11 × 10^−4^		3.21 × 10^−3^	1.75 × 10^−3^		N.A.	N.A.	

**Table 3:** Summary of sCD4 quantification. Intra-assay: The mean and standard deviation of each concentration obtained from the 54 molograms per assay were calculated to obtain intra-assay recovery rates and CVs. Inter-assay: The mean sCD4 concentration of every individual assay was defined as output. The mean and standard deviation of all three assay outputs were calculated to obtain inter-assay recovery rates and CVs.

Chip ID	Matrix	Analysis	Spiked [sCD4]	Mean [sCD4]	CV	Recovery
Chip #1	T.MACS^TM^	Intra-Assay #1	50 nM	50.15 nM	4.8%	100.3%
Chip #2	T.MACS^TM^	Intra-Assay #2	50 nM	48.91 nM	13.9%	97.8%
Chip #1	50% FBS	Intra-Assay #3	50 nM	49.52 nM	13.0%	99.0%
Inter-Chip (n = 2)	Inter-Matrix (n = 2)	Inter-Assay (n = 3)	50 nM	49.53 nM	1.2%	99.1%

## Data Availability

Raw data in addition to the data in the main manuscript and Supplementary Data are available upon request.

## Supplementary Materials

The following supporting information can be downloaded at https://www.mdpi.com/article/10.3390/bios15020066/s1. Method S1: Bio-Layer Interferometry (BLI); Method S2: Surface Plasmon Resonance (SPR); Method S3: Conjugate preparation for Molography; Method S4: SDS-PAGE; Figure S1: Representative sensorgram showing full BLI kinetic workflow; Figure S2: Representative sensorgram showing full SPR kinetic workflow; Figure S3: Representative sensorgram showing full Focal Molography (FM) kinetic workflow; Figure S4: SDS-Page of V_H_H conjugation process; Figure S5: Comparison between BLI global full versus local partial fit of the αCD4/sCD4 interaction; Figure S6: BLI: αCD4/sCD4 interaction; Figure S7: SPR: αCD4/sCD4 interaction in A) PBST, B) PBST+C, C) T.MACS^TM^ and D) 50% BS; Figure S8: FM: αCD4/sCD4 interaction; Figure S9: BLI: αGFP/GFP interaction; Figure S10: SPR: αGFP/GFP interaction in A) PBST, B) PBST+C, C) T.MACS^TM^ and D) 50% BS; Figure S11: FM: αGFP/GFP interaction; Figure S12: Baseline drift of 50% Bovine Serum (BS) on BLI, SPR and FM (αCD4/sCD4 interaction); Figure S13: Baseline drift of 50% Bovine Serum (BS) on BLI, SPR and FM (αGFP/GFP interaction); Figure S14: Baseline drift of 50% Fetal Bovine Serum (FBS) on BLI, and FM; Figure S15: Affinity matching of reference and control surfaces on SPR, BLI and FM; Figure S16: Representative sensorgrams of analysed and raw SPR data of Granzyme B (GrzB) non-specific binding in T.MACS^TM^ using an affinity matched reference flow cell; Figure S17: Representative sensorgrams of analysed and raw BLI data of Granzyme B non-specific binding in T.MACS^TM^ using affinity matched control sensors; Figure S18: Representative sensorgrams (top) and standard curves (bottom) of sCD4 quantification assay replicate in T.MACS^TM^ cell media on FM; Figure S19: Multiplex exploration analysis using sCD4 quantification data; Figure S20: Mologram distribution and data for multiplex exploration analysis using sCD4 quantification data for n = 1 to n = 6 groups; Figure S21: Mologram distribution and data for multiplex exploration analysis using sCD4 quantification data for n = 9 to n = 27 groups; Figure S22: Instrument Setup of FM; Figure S23: Top lid of FM “Callisto” reader; Figure S24: Dimensions of flow-cell sealings of different flow cells. Ref. [50] is cited in the Supplementary Materials.
