# Supplementary material for: Focal Molography Allows for Affinity and Concentration Measurements of Proteins in Complex Matrices with High Accuracy
Source: Biosensors (Basel). 2025 Jan 22;15(2):66. doi: 10.3390/bios15020066 (PMC11853488; doi:10.3390/bios15020066)
Supplement: Supplementary file 1 [file biosensors-15-00066-s001.zip › biosensors-3408240-supplementary.pdf]

Supplementary Information

Focal molography allows for affinity and concentration measurements of proteins in complex matrices with high accuracy

Lorin Dirscherl[1,†], Laura S. Merz[1,†], Ronya Kobras[1], Peter Spies[1], Andreas Frutiger[2], Volker Gatterdam[2], Dominik M. Meinel[1,*,]

[1] Institute for Chemistry and Bioanalytics, School of Life Sciences, University of Applied Sciences and Arts Northwestern Switzerland, FHNW, Hofackerstrasse 30, 4132 Muttenz, Basel-Landschaft, Switzerland
[2] Lino Biotech AG, Soodstrasse 52, 8134 Adliswil, Zurich, Switzerland
[*] Correspondence: Dominik.Meinel@FHNW.ch
[†] These authors contributed equally to this work.

Supplementary Method S1: Bio-Layer Interferometry (BLI)

BLI experiments were carried out using AR2G sensors (Sartorius, #18-5093) in parallel at a rotation speed of 1000 rpm.
Prior to binder immobilization sensor surfaces were activated by incubation in EDC/NHS. Target-specific V_HHs were immobilized to sensors via amine coupling in sodium acetate buffer pH 6. To ensure low ligand density, shown by gradual signal increase without reaching saturation [1], a maximum immobilization level of Δλ = 0.35 nm was targeted using suitable V_HH concentrations. After immobilization, sensors were quenched in C_2H_7NO. Blank reference sensors were defined as sensors functionalized with the target-specific V_HH but a blank concentration of analyte during kinetic cycle. Except where otherwise stated no binders were immobilized on negative control sensors used for testing of unspecific analyte binding. For experiments involving Granzyme B (GrzB), additional negative control sensors were included, functionalized with αGFP-V_HH and αCD45-V_HH.
For generation of baseline prior to kinetic cycles, sensors were incubated in corresponding kinetic buffers for at least 5 minutes. For experiments involving 50% BS or 50% FBS baseline was increased to 1 h.
Except where otherwise stated kinetic cycles were carried out with a 1000 s association step followed by a 1200 s dissociation step. For experiments involving GFP dissociation was increased to 1800 s. For full workflow see Supplementary Figure S1
Except where otherwise stated, for kinetic analysis, data was single referenced to the blank reference sensor and a global full 1:1 fitting model was used. For αCD4/sCD4 interaction a local partial 1:1 fitting model was used, as biphasic dissociation was observed and dissociation signal did not decay to zero [1] (Compare Supplementary Figure S5). As the partial fit is only available as a local fitting model – "kinetic parameters are derived individually for each analyte concentration" [1] – kinetic parameters represent the mean of the kinetic parameters derived for each analyte concentration. For measurements of sCD4 in 50% BS only the 2 highest analyte concentrations were included due to signal dampening.
All measurements were carried out on an Octet Red (Sartorius) with Octet Data Acquisition Software (Version 8.2.0.9). Data analysis was carried out in Octet Data Analysis Software (Version 8.2.0.7).

Supplementary Method S2: Surface Plasmon Resonance (SPR)

SPR experiments were carried out on CM5 chips (Cytiva, #240826-0911) at a flow rate of 30 µL/min.
Prior to binder immobilization chip surfaces were activated with EDC/NHS. Target-specific V_HHs were immobilized to the test flow cell via amine coupling in sodium acetate buffer pH 5 or pH 5.5. To maintain low ligand density, necessary for reliable kinetic measurements [2], a target immobilization level of 100 RU was achieved using suitable V_HH concentrations. After immobilization, the flow cell was quenched with C_2H_7NO. Unless otherwise stated, no binder was immobilized in the reference flow cell. For the experiments involving the challenging target GrzB, reference flow cell was functionalized with αGFP-V_HH analogue to the process described above to closely match the test cell's biochemical properties.
Prior to kinetic cycles, chips were primed with the corresponding kinetic buffer for at least 10 minutes. Kinetic cycles were conducted in the following sequence. 1st Start up run, 2nd Single cycle kinetics run, 3rd Blank reference run. Single-cycle kinetic run was measured with two-minute association and dissociation phases, except for the final dissociation step, which was extended to 10 minutes. Each run finishes with the regeneration procedure. Regenerated chips were re-used for kinetic experiments in different kinetic buffers and stored in PBS at 4 °C. For the experiments involving GrzB, the blank reference run was carried out before the single cycle kinetics run, to avoid introduction of GrzB to the chip prior to the kinetic run. The chip was not re-used.
Regeneration procedures were 30 s of 20 mM Sodium hydroxide for the GFP and GrzB analyte, and 30 s of 1 mM hydrochloric acid solution followed by 30 s of 20 mM Sodium hydroxide for the sCD4 analyte. Each regeneration procedure was followed by a stabilization phase of 600 s in running buffer. Except where otherwise stated for experiments involving 50% BS, stabilization phase was increased to 1 h to ensure baseline stability. For full workflow see Supplementary Figure S2.
For kinetic analysis, data was double referenced to the reference cell and the blank reference run. A 1:1 interaction model was used for fitting.
All measurements were conducted on a Biacore X100 using Biacore X100 Control Software (Version 2.0.1). Data analysis was performed Biacore X100 Evaluation Software (Version 2.0.1).

Supplementary Method S3: Conjugate preparation for Molography

The following protocol describes the conjugation process of maleimide-functionalized ssDNA strands towards V_HHs with C-terminal cystein and His-tag. Except were otherwise stated all steps were carried out at room temperature in PBS, 500 mM NaCl pH 7,4.
To remove potential aggregates V_HH solutions were initially centrifuged at 18'000 x g for 5 minutes using an Eppendorf centrifuge 5425R. Supernatant was retrieved, and protein concentration was determined using a DeNovix DS-11 FX+ Spectrophotometer/Fluorometer.
V_HH cysteine residues were reduced by incubation with TCEP at a V_HH:TCEP molar ratio of 1:1.5 for 1 hour at 800 rpm on an Eppendorf ThermoMixer C. Subsequently, reduced V_HHs were conjugated to ssDNA at a V_HH:ssDNA molar ratio of 1:5, for 90 minutes at 800 rpm. Any remaining free cysteines were quenched by adding ethylmaleimide at a V_HH:Ethylmaleimid molar ratio of 1:10 for 15 minutes at 800 rpm.
Following conjugation, the V_HH-ssDNA conjugates were purified using the HisPur™ Ni-NTA Purification Kit (Thermo Fisher Scientific, #88224), following the suppliers protocol. As Equilibration and Wash buffer 20 mM NaH_2PO_4, 500 mM NaCl pH 7.4 was used. As Elution Buffer 20 mM NaH_2PO_4, 500 mM NaCl, 350 mM imidazole, pH 7.4. Elution was carried out twice and fractions were pooled.
Final buffer exchange of elution in PBST was performed using 7 K MWCO Zeba Spin Desalting Columns (Thermo Fisher Scientific, #89882), following the suppliers protocol.
Successful conjugation was checked with SDS PAGE (Compare Supplementary Method S4 and Figure S4)

Supplementary Method S4: SDS-PAGE

V_HH-conjugates were analyzed via SDS-PAGE. SDS-PAGE was carried out using precast SurePAGE™ 4 - 12% Bis-Tris Gels (GenScript, #M00654) according to manufacturer instructions with MES running Buffer (GenScript, #M00677) and Precision plus Protein Dual Color Standard (Biorad, #1610374EDU). As 4 x non reducing SDS sample dye we used 250 mM Tris-HCL, 8% SDS, 40% Glycerol, 0.04% Bromophenol blue, pH 6.8. For 4x reducing SDS sample dye we added 20% 2-Mercaptoethanol to the recipe above. Gels were stained for at least 30 minutes using QuickBlue Protein Stain (LubioScience, #LU001000) prior to image acquisition using a (Canon ImageRUNNER Advance DX, C5740i).

Supplementary Figures

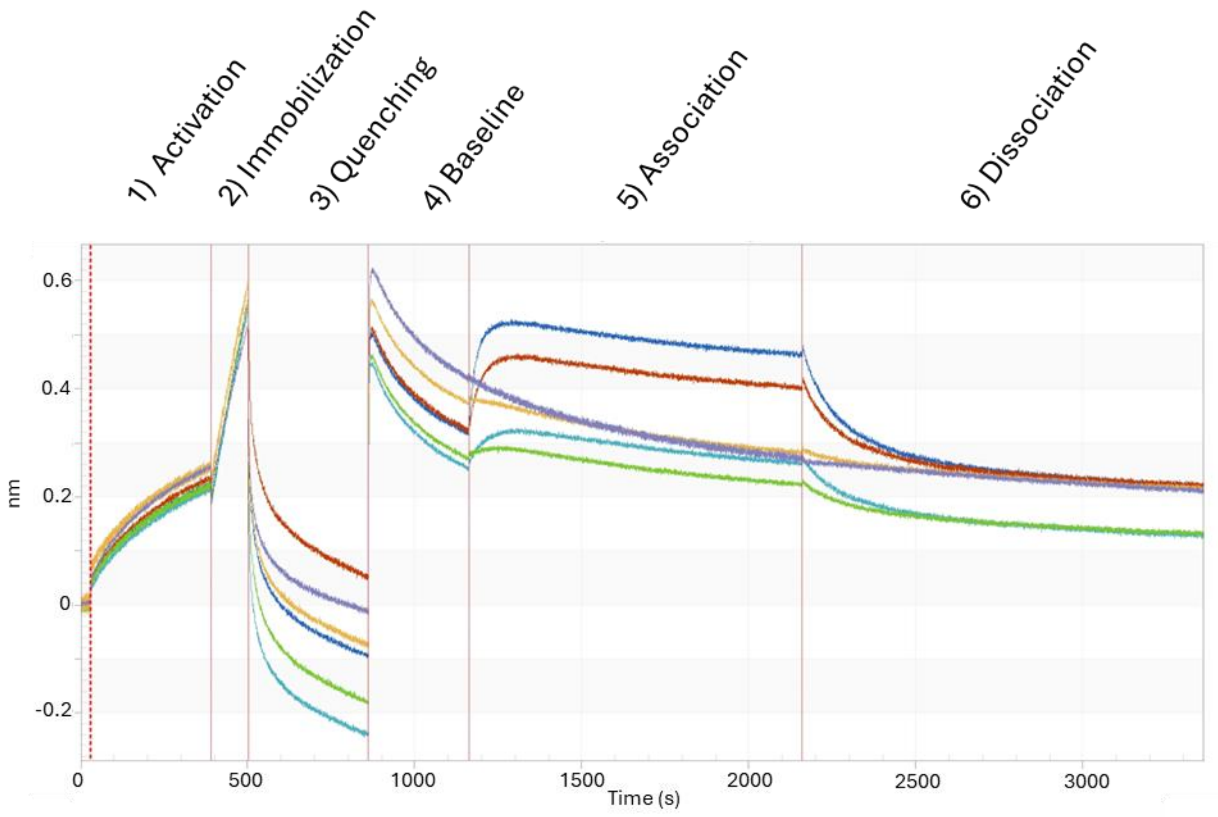

Supplementary Figure S1: *Representative sensorgram showing full BLI kinetic workflow.*

This graph shows raw BLI data of the αCD4/sCD4 interaction in PBST+C. 1) Activation of sensors. Carboxyl (Amine reactive) sensors are activated using EDC/NHS solution. 2) Immobilization of binder. 50 nM αCD4-V$_H$H is immobilized onto sensors. 3) Quenching. Sensors are quenched using 1 M Ethanolamine. 4) Baseline. Baseline in kinetic buffer (here: PBST+C) is generated. 5) Association. Binding of sCD4 (dark blue = 100 nM, red = 40 nM, light blue = 16 nM, green = 6.4 nM, yellow = 2.56 nM, purple = 0 nM) to the functionalized sensors. 6) Dissociation of the αCD4/sCD4 complex.

Sensors are discarded after each measurement.
Prior to kinetic evaluation data is single reference to the blank run (purple)

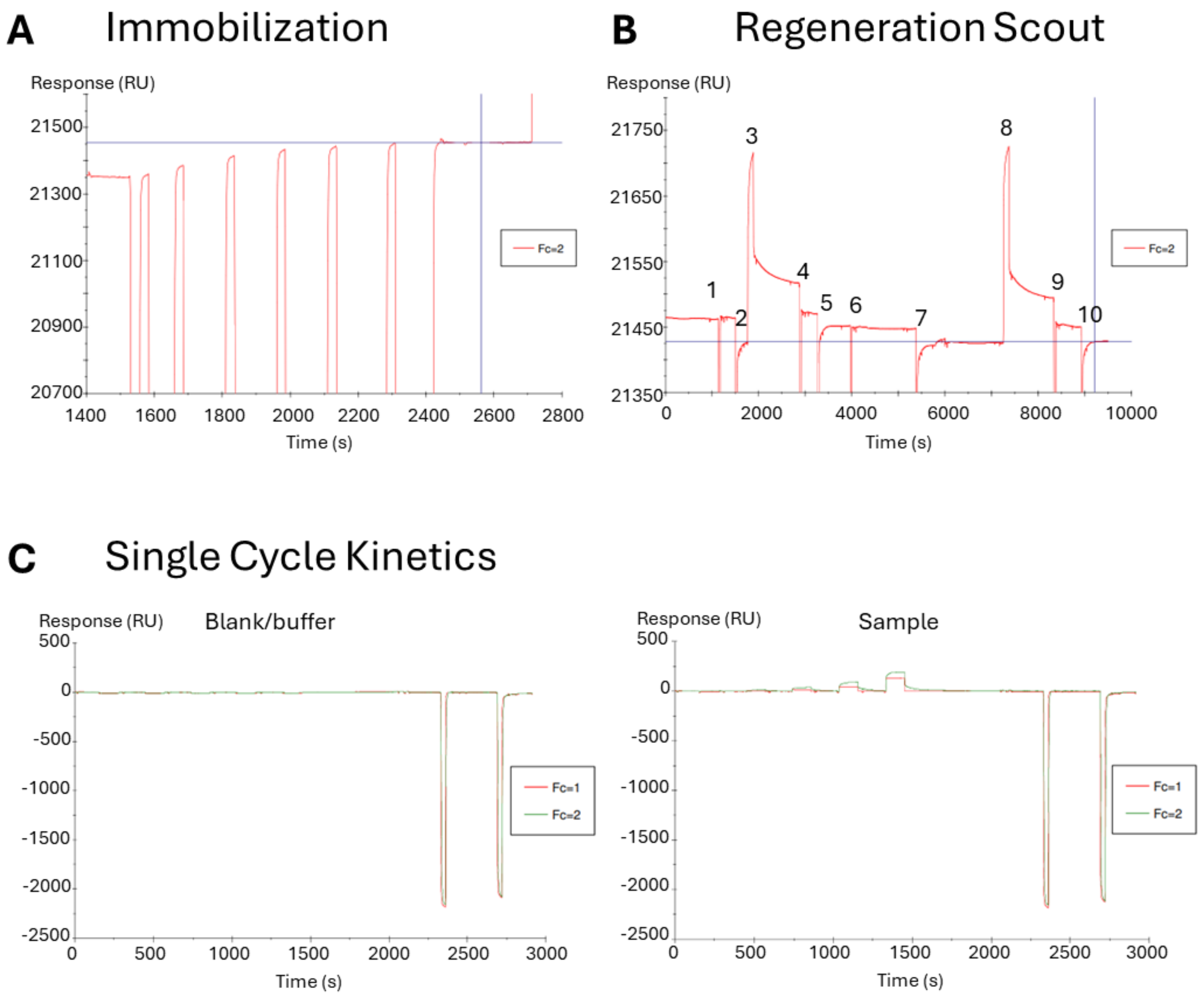

Supplementary Figure S2: *Representative sensorgram showing full SPR kinetic workflow.*

This graph shows raw SPR data of the αCD4/sCD4 interaction.

A) Immobilization of binder. αCD4-V$_H$H (5 ug/ml) is immobilized to test flow cell (FC2) up to 100 RU in PBST. No binder is immobilized to reference flow cell (FC1, data not shown).

B) Regeneration Scout. Manual run in PBS-T to investigate binding and regeneration effectiveness. 1, 4, 6 and 9 show regeneration with 1 mM HCl (pH 3), 2, 5, 7 and 10 show regeneration with 20 mM NaOH, 3 and 8 show sample sCD4 (100 nM) binding to the functionalized test flow cell (FC2). Data shows that sequential injections of 1 mM HCL (pH 3) and 20 mM NaOH result in successful regeneration of sCD4 from αCD4-V$_H$H.

C) Blank reference and kinetic run.
Left: Blank reference run. In this run a blank sample (here: PBST+C buffer) was injected 5 times followed by the two-step regeneration procedure: 1 mM HCL and 20 mM NaOH. Signals are shown for both test flow cell (FC2, green) and reference flow cell (FC1, red)
Right: Kinetic run. In this run the sCD4 samples (2.6 nM, 6.4 nM, 16 nM, 40 nM and 100 nM here: in PBST+C buffer) were injected serially followed by the two-step regeneration procedure. Signals are shown for both test flow cell (FC2, green) and reference flow cell (FC1, red)

Regenerated chips were re-used for characterization of the same interaction in different kinetic buffers.
Prior to kinetic evaluation data is double referenced to the reference cell and blank reference run.

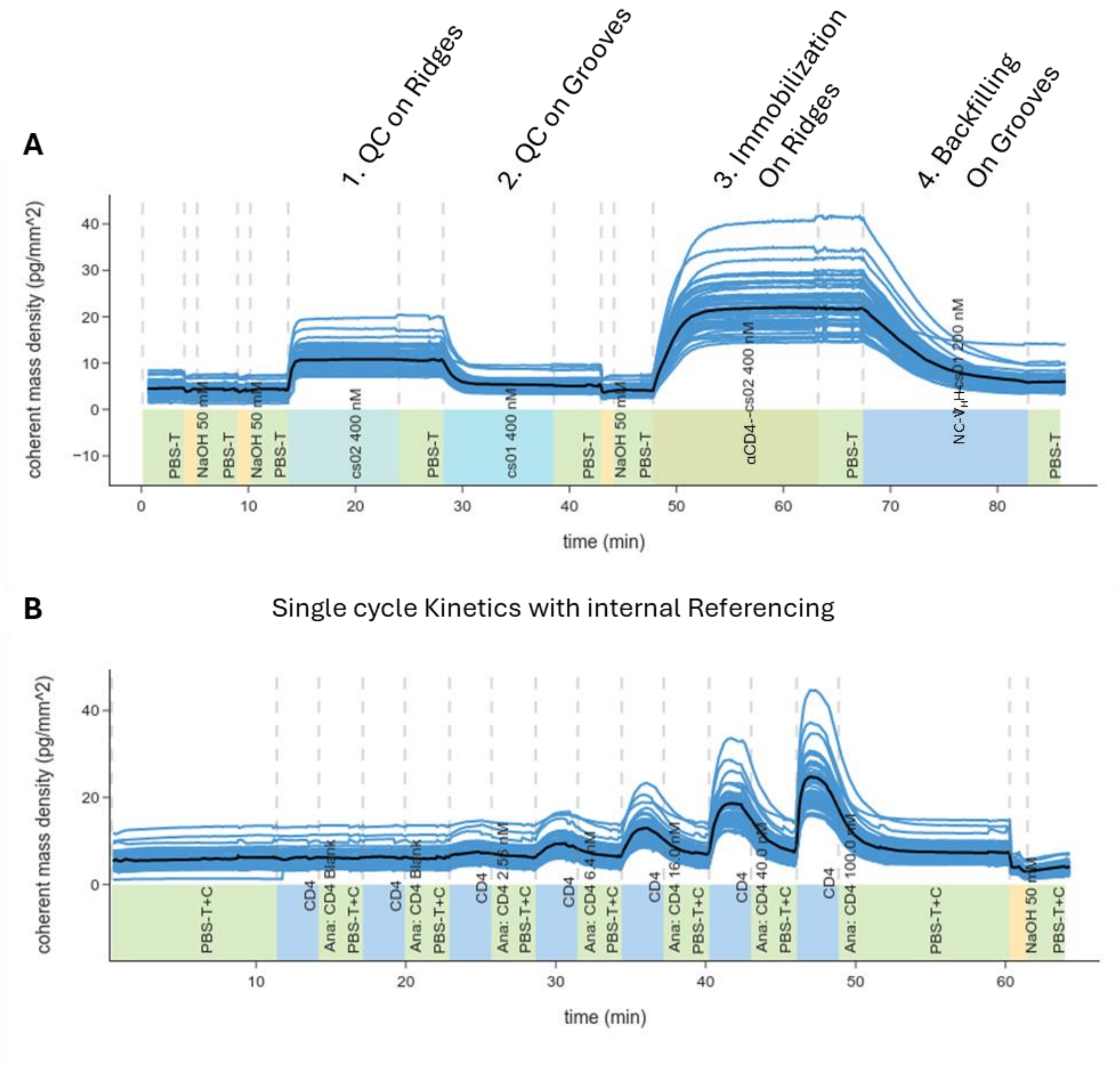

Supplementary Figure S3: *Representative sensorgram showing full Focal Molography (FM) kinetic workflow.*

This graphs show raw FM data of the αCD4/sCD4 interaction on a chip with s02|s01 architecture (ridges|grooves).

A: Quality control (QC) of the chip and immobilization of the V_HH. 1. cs02 ssDNA was injected to assess binding capacity of the ridges 2. cs01 ssDNA was injected to assess binding capacity of the grooves followed by regeneration (50 mM NaOH) to remove the ssDNA used during QC. 3. αCD4-V_HH-cs02 conjugates were injected to functionalize ridges. 4. NC-V_HH-cs01 conjugates (control) were backfilled into grooves.
All QCs and immobilization were carried out using PBST as running buffer. Prior to kinetic measurement, the buffer was exchanged with corresponding kinetic buffer.

B: Kinetic measurement of αCD4/sCD4 in PBST+C. First, two blank PBST+C injections, then the five concentrations of sCD4 (2.6 nM, 6.4 nM, 16 nM, 40 nM and 100 nM) were injected. Finally, the Chip was regenerated (50 mM NaOH) to remove V_HH-conjugates.

Regenerated chips were re-used for characterization of interactions in different kinetic buffers using fresh V_HH-conjugates.
No referencing was necessary prior to kinetic evaluation.

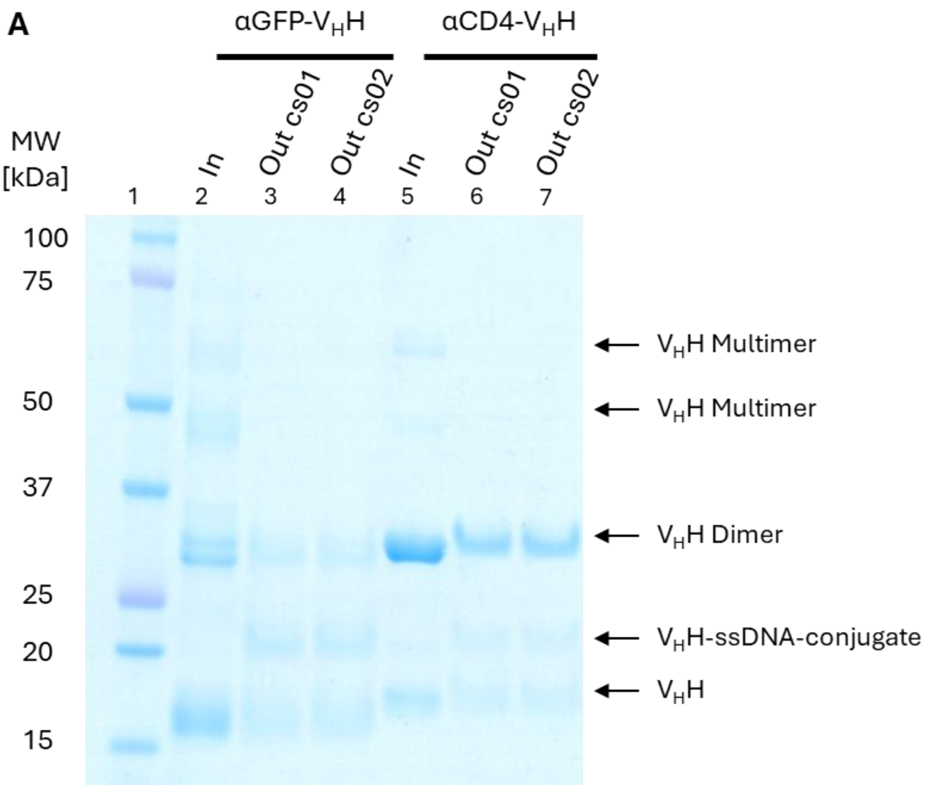

Supplementary Figure S4: *SDS-Page of V_HH conjugation process.*

ssDNA (cs01 and cs02) was conjugated to the V_HH binders. 1) Ladder (Precision Plus Protein Dual Color Standards, Biorad, #1610374); 2) Input αGFP-V_HH prior to the conjugation; 3 and 4) Output αGFP-V_HH-conjugates. Outputs after conjugation and purification procedure; 5) Input αCD4-V_HH; 6 and 7) Output αCD4-V_HH-conjugates.

For all conjugation procedures V_HH-conjugates were observed at ~20 kDa. Unconjugated V_HH run at ~16 kDa and V_HH Dimers at ~ 32 kDa.

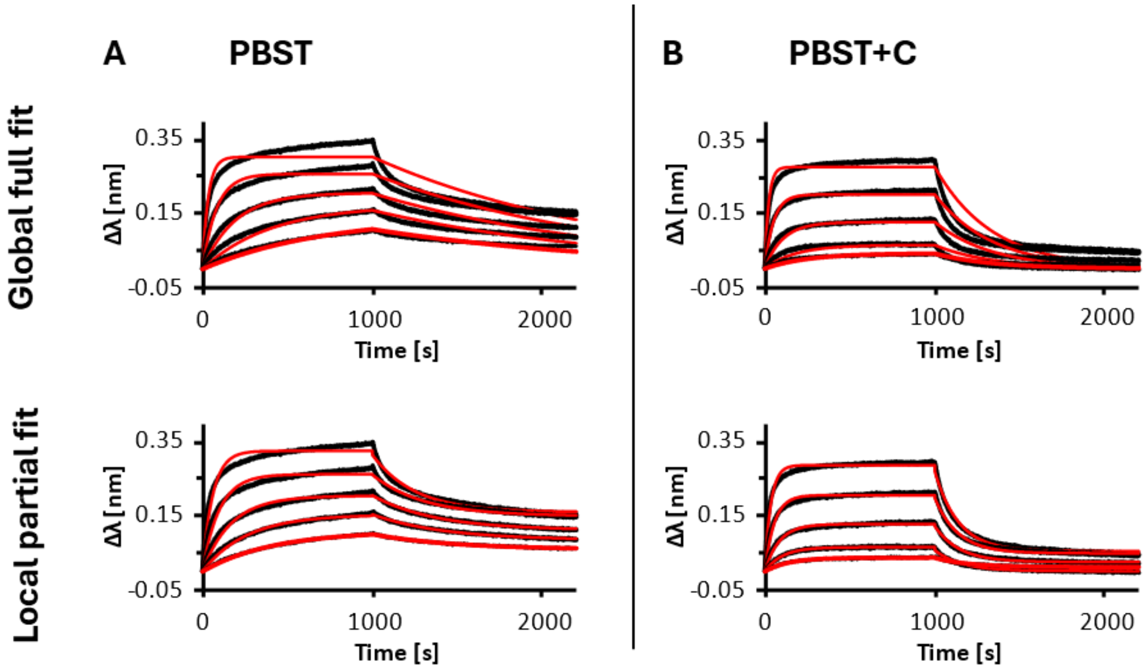

Supplementary Figure S5: *Comparison between BLI global full versus local partial fit of the αCD4/sCD4 interaction.*

A) Measured in PBST, analysed with global full fit (top) and local partial fit (bottom) and B) measured in PBST+C, analysed with global full fit (top) and local partial fit (bottom)

Dissociation shows a clear biphasic behavior, especially in PBST, and the pre-association baseline is not reached. The additional blocking agent Casein (PBST+C) helps reduce this effect. Therefore, it can be assumed, that this biphasic behavior either comes from unspecific binding or rebinding effects. In accordance with Sartorius guidelines [1]*, we applied a local partial fit for kinetic analysis of sCD4 interaction due to biphasic behavior. Partial fitting may tend to give higher* k_{off} *values* [1]*. Kinetic parameters obtained by local partial fitting represent the mean of the kinetic parameters obtained by the individual concentration curves.*

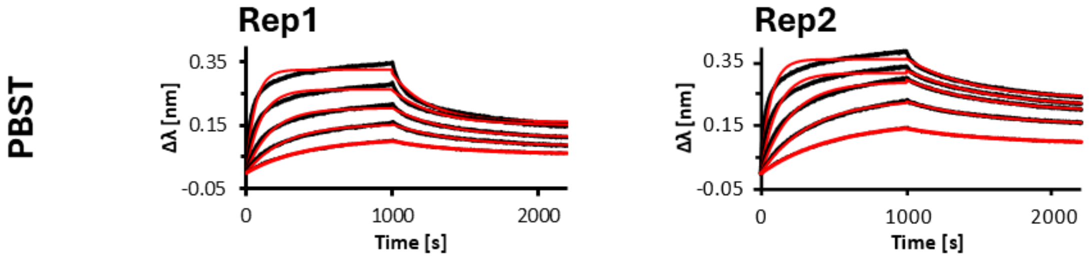

	Rep 1		Rep 2	
	value	SD	value	SD
KD (M)	1.79E-08	8.69E-09	1.16E-08	3.51E-09
kon(1/Ms)	1.76E+05	4.30E+04	1.66E+05	2.75E+04
kdis(1/s)	2.91E-03	7.79E-04	1.85E-03	2.37E-04

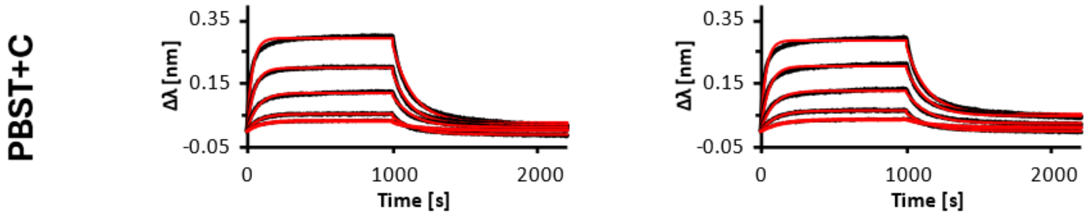

	Rep 1		Rep 2	
	value	SD	value	SD
KD (M)	1.79E-08	1.36E-08	2.03E-08	1.13E-08
kon(1/Ms)	7.28E+05	6.38E+05	4.33E+05	2.56E+05
kdis(1/s)	6.99E-03	7.22E-04	6.62E-03	8.77E-04

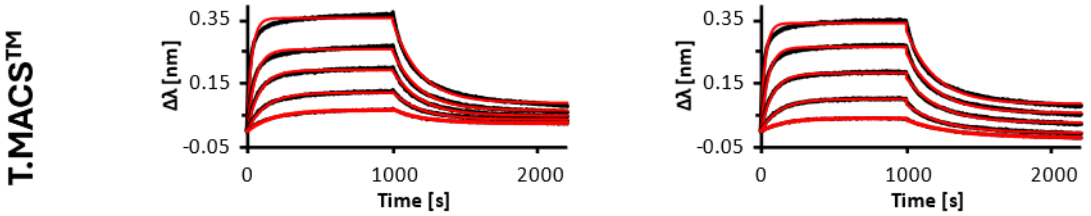

	Rep 1		Rep 2	
	value	SD	value	SD
KD (M)	1.60E-08	3.64E-09	9.58E-09	5.98E-09
kon(1/Ms)	3.38E+05	7.34E+04	6.24E+05	4.17E+05
kdis(1/s)	5.20E-03	2.57E-04	4.22E-03	7.79E-04

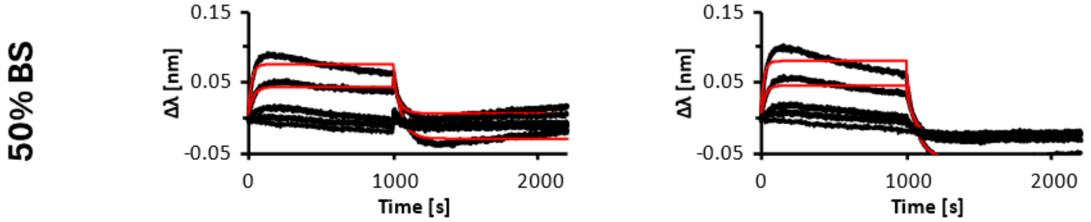

	Rep 1		Rep 2	
	value	SD	value	SD
KD (M)	1.53E-07	1.10E-07	4.06E-08	3.80E-09
kon(1/Ms)	1.82E+05	9.93E+04	4.06E+05	8.03E+04
kdis(1/s)	2.23E-02	4.88E-03	1.63E-02	1.73E-03

Supplementary Figure S6: *BLI: αCD4/sCD4 interaction. Individual replicates, corresponding fits and kinetic parameters.*

Individual sensors were functionalized with αCD4-V$_H$H (representative workflow shown in Supplementary Figure S1). The rows from top to bottom show PBST, PBST+C, T.MACS™ and 50% Bovine Serum (BS) as matrix. Following sCD4 concentrations were measured 2.6 nM, 6.4 nM, 16 nM, 40 nM and 100 nM. We used a local partial fit for the analysis of the data. Kinetic parameters obtained by local partial fitting represent the mean and standard deviation (SD) of the kinetic parameters obtained by the individual concentration curves. Due to signal dampening in 50% BS only the two highest concentrations (40 nM and 100 nM) were included for calculation of the mean.

Chip 1

Quality Control

- ✓ Reported kinetic constants are within instrument specifications.
- ✓ Kinetic constants appear to be uniquely determined.
- ✓ No significant bulk contributions (RI) found.
- ➡ Check that sensorgrams have sufficient curvature.
- ➡ Examine the residual plot. Pay attention to systematic and non-random deviations.

Report table

Curve	ka (1/Ms)	kd (1/s)	KD (M)	Rmax (RU)	Conc (M)	tc	Flow (ul/min)	kt (RU/Ms)	RI (RU)	Chi² (RU²)	U-value
	2.563E+5	5.643E-4	2.202E-9	108.4		2.887E+21				9.77	2
Cycle: 2					2.560E-9		30.00	8.972E+21	2.136		
					6.400E-9				4.687		
					1.600E-8				7.742		
					4.000E-8				7.026		
					1.000E-7				7.340		

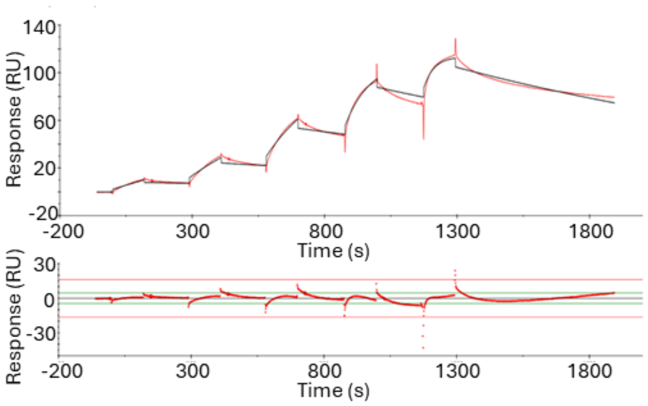

Chip 2

Quality Control

- ✓ Reported kinetic constants are within instrument specifications.
- ✓ Kinetic constants appear to be uniquely determined.
- ✓ No significant bulk contributions (RI) found.
- ➡ Check that sensorgrams have sufficient curvature.
- ➡ Examine the residual plot. Pay attention to systematic and non-random deviations.

Report table

Curve	ka (1/Ms)	kd (1/s)	KD (M)	Rmax (RU)	Conc (M)	tc	Flow (ul/min)	kt (RU/Ms)	RI (RU)	Chi² (RU²)	U-value
	2.897E+5	6.093E-4	2.104E-9	122.9		4.783E+21				18.7	2
Cycle: 2					2.560E-9		30.00	1.486E+22	4.343		
					6.400E-9				8.343		
					1.600E-8				12.40		
					4.000E-8				12.05		
					1.000E-7				14.11		

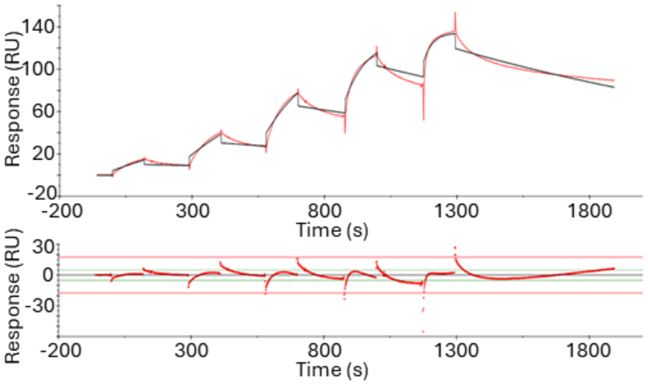

Supplementary Figure S7A: *SPR: αCD4/sCD4 interaction in PBST. Individual replicates, corresponding fits, SPR quality control (QC) information and kinetic parameters.*

Individual chips were functionalized with αCD4-V$_H$H (representative workflow shown in Supplementary Figure S2). Following sCD4 concentrations were measured 2.6 nM, 6.4 nM, 16 nM, 40 nM and 100 nM. From each chip the fit of the measurement, the kinetic parameters and the QC output of the instrument are shown.

All evaluations passed SPR QC.

For SPR αCD4 interaction analyses, regenerated chips were re-used for kinetic characterization in the different kinetic buffers (PBST, PBST+C, T.MACS™ and 50% Bovine Serum). Replicates indicated by the same chip number were carried out on the same chip (same immobilization).

Chip 1

Quality Control

- Reported kinetic constants are within instrument specifications.
- Kinetic constants appear to be uniquely determined.
- High bulk contributions (RI) found.
- Check that sensorgrams have sufficient curvature.
- Examine the residual plot. Pay attention to systematic and non-random deviations.

Report table

Curve	ka (1/Ms)	kd (1/s)	KD (M)	Rmax (RU)	Conc (M)	tc	Flow (ul/min)	kt (RU/Ms)	RI (RU)	Chi² (RU²)	U-value
	8.132E+5	0.02815	3.462E-8	75.55		1.034E+7				9.06	9
Cycle: 2					2.560E-9		30.00	3.214E+7	0.1399		
					6.400E-9				0.9306		
					1.600E-8				2.541		
					4.000E-8				3.628		
					1.000E-7				3.947		

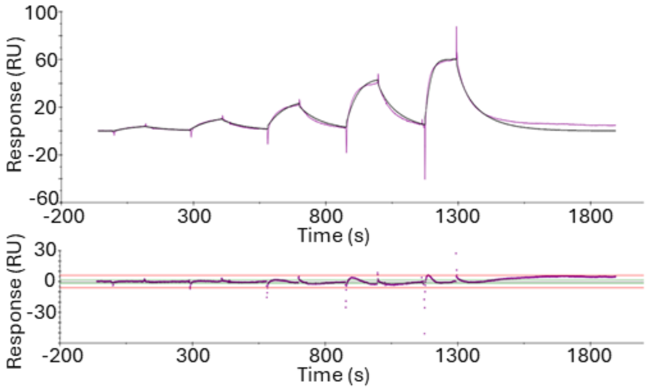

Chip 2

Quality Control

- Reported kinetic constant kd is outside and kinetic constant ka is approaching the limits that can be measured by the instrument.
- Kinetic constants cannot be uniquely determined.
- High bulk contributions (RI) found.
- Check that sensorgrams have sufficient curvature.
- Examine the residual plot. Pay attention to systematic and non-random deviations.

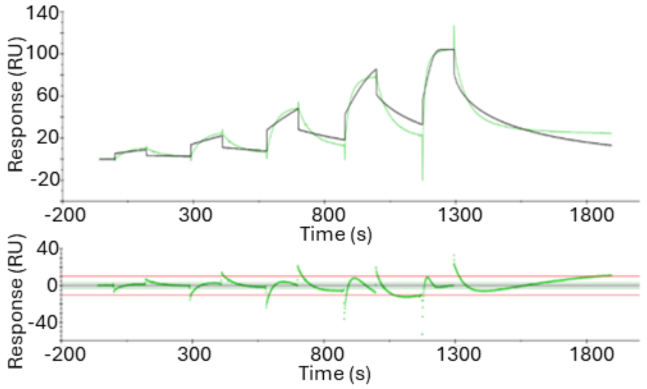

Supplementary Figure S7B: *SPR: αCD4/sCD4 interaction in PBST+C. Individual replicates, corresponding fits, SPR quality control (QC) information and kinetic parameters.*

Evaluation for chip 2 did not pass SPR QC. Therefore, kinetic parameters (αCD4 interaction, PBST+C, Chip 2) were not included for the kinetic comparison study.

Chip 1

Quality Control

✓	Reported kinetic constants are within instrument specifications.
✓	Kinetic constants appear to be uniquely determined.
!	High bulk contributions (RI) found.
→	Check that sensorgrams have sufficient curvature.
→	Examine the residual plot. Pay attention to systematic and non-random deviations.

Report table

Curve	ka (1/Ms)	kd (1/s)	KD (M)	Rmax (RU)	Conc (M)	tc	Flow (ul/min)	kt (RU/Ms)	RI (RU)	Chi² (RU²)	U-value
	7.866E+5	0.01544	1.962E-8	92.68		3.373E+7				9.79	4
Cycle: 2					2.560E-9		30.00	1.048E+8	0.7292		
					6.400E-9				2.788		
					1.600E-8				6.058		
					4.000E-8				7.710		
					1.000E-7				10.12		

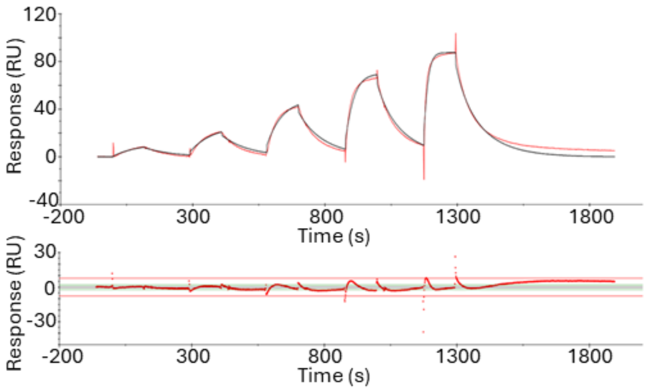

Chip 2

Quality Control

✓	Reported kinetic constants are within instrument specifications.
✓	Kinetic constants appear to be uniquely determined.
!	High bulk contributions (RI) found.
→	Check that sensorgrams have sufficient curvature.
→	Examine the residual plot. Pay attention to systematic and non-random deviations.

Report table

Curve	ka (1/Ms)	kd (1/s)	KD (M)	Rmax (RU)	Conc (M)	tc	Flow (ul/min)	kt (RU/Ms)	RI (RU)	Chi² (RU²)	U-value
	1.175E+6	0.01912	1.627E-8	110.0		2.987E+7				18.9	5
Cycle: 2					2.560E-9		30.00	9.283E+7	2.900		
					6.400E-9				5.357		
					1.600E-8				8.542		
					4.000E-8				7.681		
					1.000E-7				10.17		

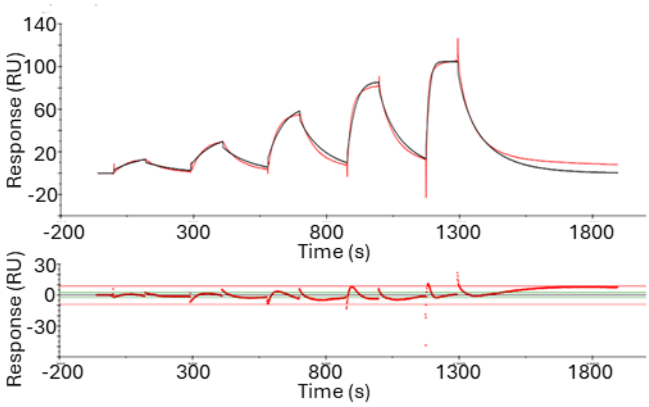

Supplementary Figure S7C: *SPR: αCD4/sCD4 interaction in T.MACS™. Individual replicates, corresponding fits, SPR quality control (QC) information and kinetic parameters.*

All evaluations passed SPR QC.

Chip 2

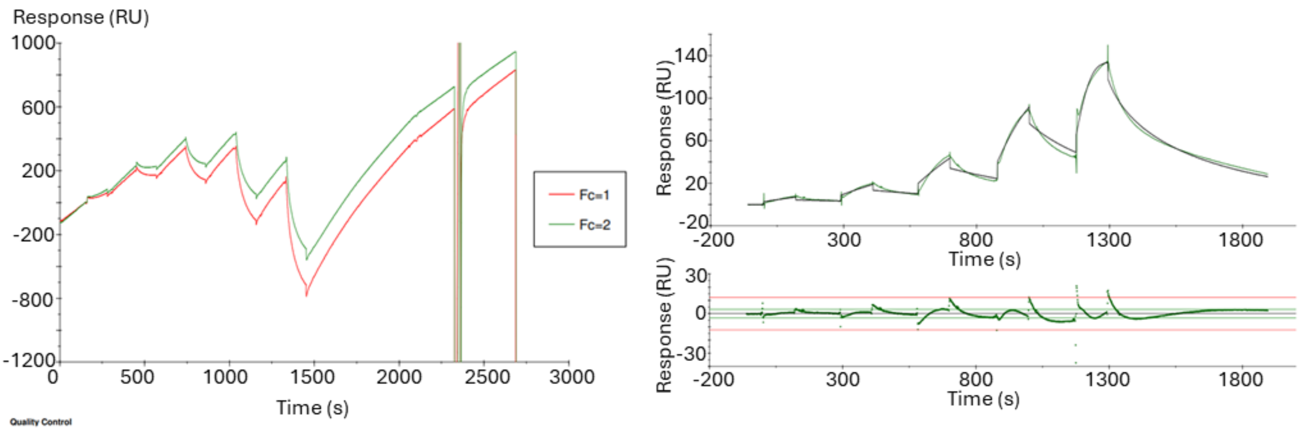

Quality Control

✓	Reported kinetic constants are within instrument specifications.
✓	Kinetic constants appear to be uniquely determined.
!	High bulk contributions (RI) found.
→	Check that sensorgrams have sufficient curvature.
→	Examine the residual plot. Pay attention to systematic and non-random deviations.

Report table

Curve	ka (1/Ms)	kd (1/s)	KD (M)	Rmax (RU)	Conc (M)	tc	Flow (ul/min)	kt (RU/Ms)	RI (RU)	Chi² (RU²)	U-value
	7.553E+5	0.01005	1.331E-8	134.5		5.920E+6				11.5	5
Cycle: 2					2.560E-9		30.00	1.839E+7	2.654		
					6.400E-9				5.456		
					1.600E-8				9.604		
					4.000E-8				15.53		
					1.000E-7				15.62		

Supplementary Figure S7D: *SPR: αCD4/sCD4 interaction in 50% Bovine Serum (BS). Individual replicates, corresponding fits, SPR quality control (QC) information and kinetic parameters.*

Left sensorgram: Unreferenced raw data of kinetic run of test flow cell (FC2, green) and control flow cell (FC1, red). Right sensorgram: Double referenced data and fit. αCD4/sCD4 interaction in 50% BS was carried out as last measurement on the chip. Injection of sCD4 during kinetic run resulted in negative signals on SPR raw data (left sensorgram). Double referenced signals were again positive (right sensorgram). Evaluation passed SPR QC. However, as the negative signal upon sCD4 injection is not fully understood, the SPR results in 50% BS should be read with caution.

	Chip1 (s02|s01)	Chip2 (s01|s02)

PBST

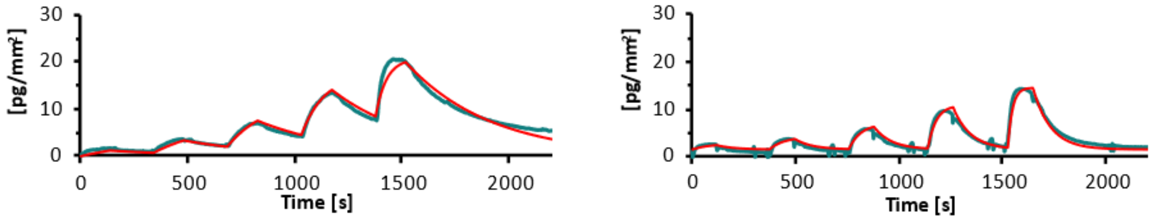

	Rep 1 (E13)	Rep 2 (G15)
KD (M)	1.62E-08	3.75E-08
kon(1/Ms)	1.51E+05	2.85E+05
kdis(1/s)	2.44E-03	1.05E-02

PBST+C

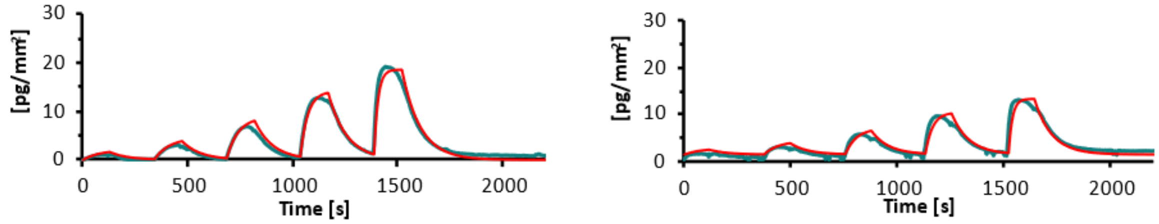

	Rep 1 (E13)	Rep 2 (G15)
KD (M)	2.46E-08	2.99E-08
kon(1/Ms)	4.65E+05	4.67E+05
kdis(1/s)	1.13E-02	1.38E-02

T.MACS^{TM}

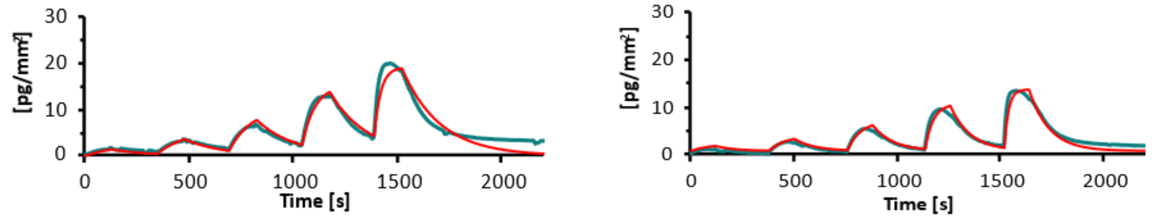

	Rep 1 (E13)	Rep 2 (G15)
KD (M)	3.40E-08	2.64E-08
kon(1/Ms)	2.38E+05	3.83E+05
kdis(1/s)	8.19E-03	1.02E-02

50% BS

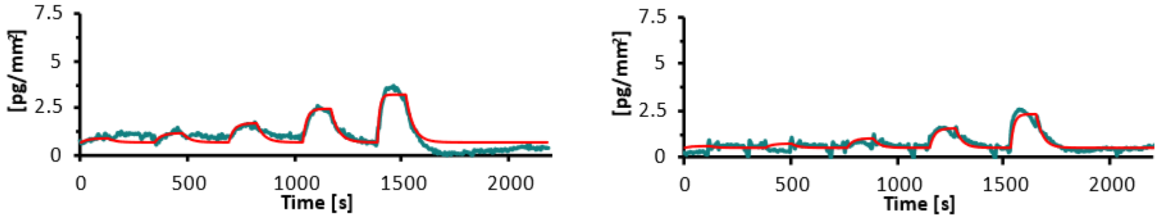

	Rep 1 (E13)	Rep 2 (G15)
KD (M)	3.75E-08	5.78E-08
kon(1/Ms)	5.16E+05	4.68E+05
kdis(1/s)	1.90E-02	2.63E-02

Supplementary Figure S8: *FM: αCD4/sCD4 interaction. Representative sensorgrams of individual replicates corresponding fits and kinetic parameters.*

Two individual chips, with inversed mologram architecture (s02|s01 vs s01|s02) were functionalized with αCD4-V_HH-conjugates and backfilled with NC-V_HH-conjugates. For FM αCD4 interaction analyses, regenerated chips were re-used for kinetic characterization in the different kinetic buffers (PBST, PBST+C, T.MACS^{TM} and 50% Bovine Serum) using fresh V_HH-conjugates. (representative work-flow shown in Supplementary Figure S3). Kinetic parameters correspond to the median kinetic parameters obtained by analysis of all 54 molograms on a single chip. Note that they do not necessarily correspond to the values of the median mologram seen in the graphs as each kinetic parameter has its own median and the Figure shows the mologram with median signal intensity.

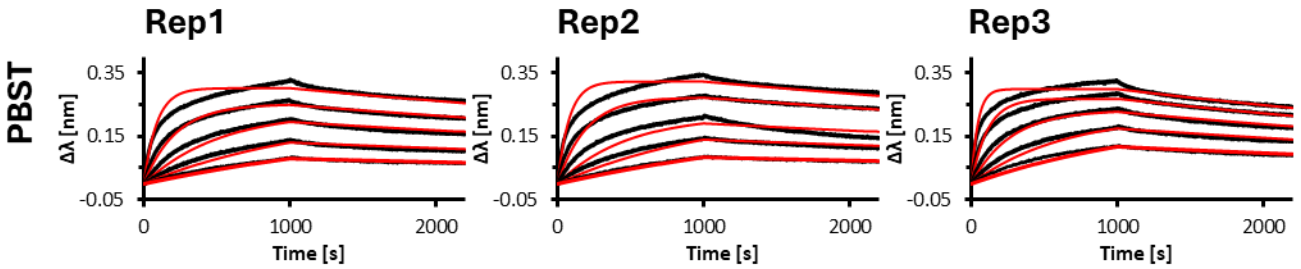

	Rep 1		Rep 2		Rep 3	
	value	Error	value	Error	value	Error
KD (M)	6.40E-10	5.38E-12	4.17E-10	2.97E-12	4.94E-10	5.62E-12
kon(1/Ms)	2.25E+05	1.21E+03	4.48E+05	2.40E+03	2.52E+05	1.71E+03
kdis(1/s)	1.44E-04	9.26E-07	1.87E-04	8.72E-07	1.25E-04	1.14E-06

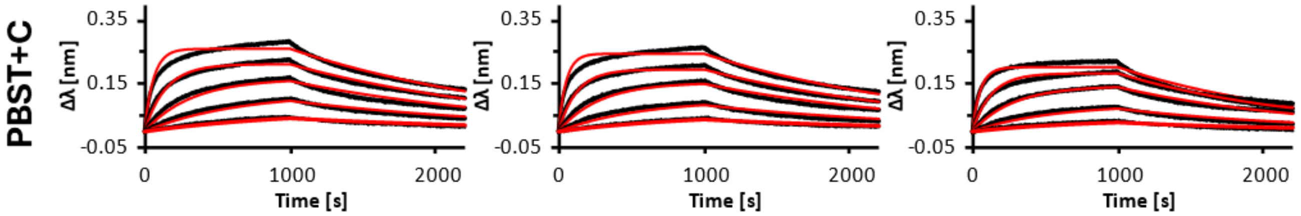

	Rep 1		Rep 2		Rep 3	
	value	Error	value	Error	value	Error
KD (M)	1.90E-09	1.23E-11	1.64E-09	1.11E-11	2.17E-09	1.74E-11
kon(1/Ms)	3.16E+05	1.91E+03	3.73E+05	2.37E+03	3.51E+05	2.65E+03
kdis(1/s)	5.99E-04	1.40E-06	6.12E-04	1.45E-06	7.62E-04	1.99E-06

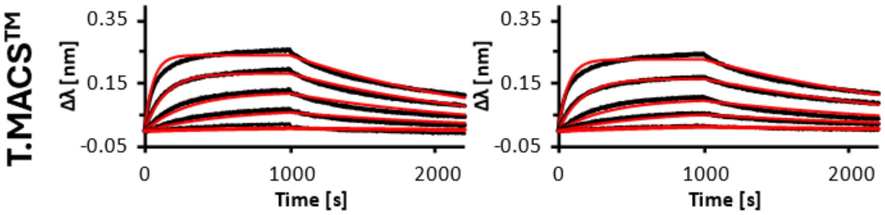

	Rep 1		Rep 2	
	value	Error	value	Error
KD (M)	2.10E-09	1.74E-11	2.00E-09	1.43E-11
kon(1/Ms)	3.29E+05	2.57E+03	2.83E+05	1.88E+03
kdis(1/s)	6.91E-04	1.94E-06	5.66E-04	1.52E-06

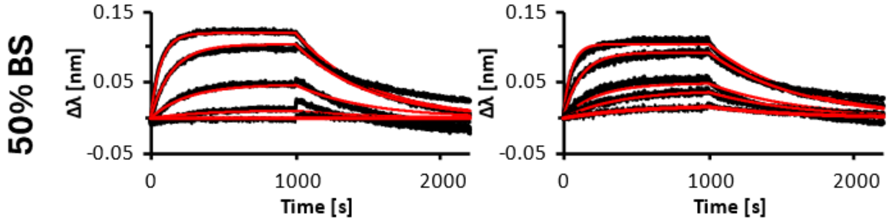

	Rep 1		Rep 2	
	value	Error	value	Error
KD (M)	8.13E-09	1.54E-10	4.78E-09	6.63E-11
kon(1/Ms)	2.43E+05	4.40E+03	3.37E+05	4.47E+03
kdis(1/s)	1.98E-03	1.12E-05	1.61E-03	6.34E-06

Supplementary Figure S9: *BLI: αGFP/GFP interaction. Individual replicates, corresponding fits and kinetic parameters.*

Individual sensors were functionalized with αGFP-$V_{H}H$ (representative workflow shown in Supplementary Figure S1). The rows from top to bottom show PBST, PBST+C, T.MACS^{TM} and 50% Bovine Serum (BS) as matrix. Following GFP concentrations were measured 1.3 nM, 3.2 nM, 8 nM, 20 nM and 50 nM. We used a global full fit for the analysis of the data.

Chip 1

Quality Control

- Reported kinetic constants are within instrument specifications.
- Kinetic constants appear to be uniquely determined.
- High bulk contributions (RI) found.
- Check that sensorgrams have sufficient curvature.
- Examine the residual plot. Pay attention to systematic and non-random deviations.

Report table

Curve	ka (1/Ms)	kd (1/s)	KD (M)	Rmax (RU)	Conc (M)	tc	Flow (ul/min)	kt (RU/Ms)	RI (RU)	Chi² (RU²)	U-value
	2.591E+6	0.01589	6.131E-9	52.54		3.607E+6				1.72	9
Cycle: 2					1.280E-9		30.00	1.121E+7	0.7367		
					3.200E-9				1.589		
					8.000E-9				3.161		
					2.000E-8				4.209		
					5.000E-8				1.999		

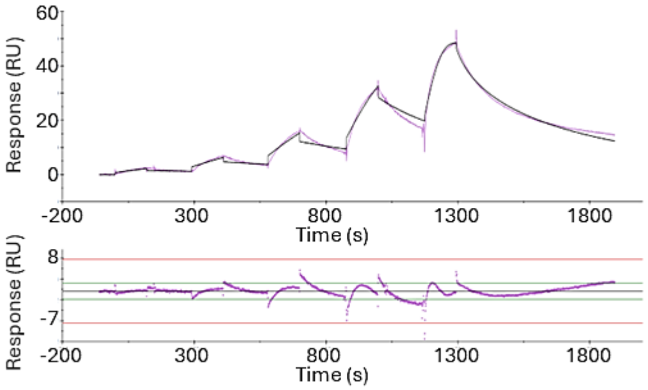

Chip 2

Quality Control

- Reported kinetic constants are within instrument specifications.
- Kinetic constants appear to be uniquely determined.
- No significant bulk contributions (RI) found.
- Check that sensorgrams have sufficient curvature.
- Examine the residual plot. Pay attention to systematic and non-random deviations.

Report table

Curve	ka (1/Ms)	kd (1/s)	KD (M)	Rmax (RU)	Conc (M)	tc	Flow (ul/min)	kt (RU/Ms)	RI (RU)	Chi² (RU²)	U-value
	7.154E+5	0.001764	2.466E-9	139.9		1.331E+7				9.77	2
Cycle: 2					1.280E-9		30.00	4.135E+7	1.484		
					3.200E-9				3.727		
					8.000E-9				7.712		
					2.000E-8				9.740		
					5.000E-8				6.457		

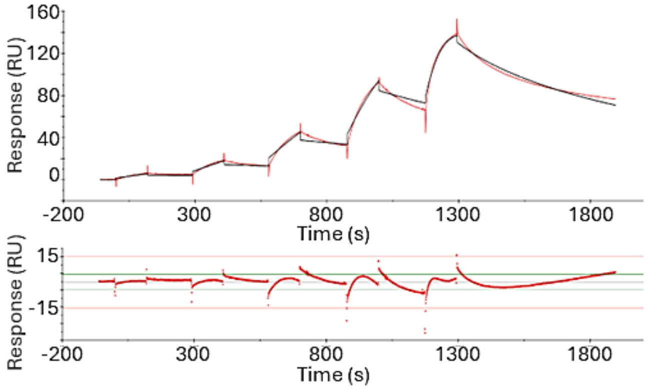

Supplementary Figure S10A: *SPR: αGFP/GFP interaction in PBST. Individual replicates, corresponding fits, SPR quality control (QC) information and kinetic parameters.*

Individual chips were functionalized with αGFP-V$_H$H (representative workflow shown in Supplementary Figure S2). Following GFP concentrations were 1.3 nM, 3.2 nM, 8 nM, 20 nM and 50 nM. From each chip the fit of the measurement, the kinetic parameters and the QC output of the instrument are shown.

All evaluations passed SPR QC.

For SPR αGFP/GFP interaction analyses, regenerated chips were re-used for kinetic characterization in the different kinetic buffers (PBST, PBST+C, T.MACS^{TM} and 50% Bovine Serum). Replicates indicated by the same chip number were carried out on the same chip (same immobilization).

Chip 1

Quality Control

!	Reported kinetic constant kd is approaching the limits that can be measured by the instrument.
✓	Kinetic constants appear to be uniquely determined.
✓	No significant bulk contributions (RI) found.
→	Check that sensorgrams have sufficient curvature.
→	Examine the residual plot. Pay attention to systematic and non-random deviations.

Report table

Curve	ka (1/Ms)	kd (1/s)	KD (M)	Rmax (RU)	Conc (M)	tc	Flow (ul/min)	kt (RU/Ms)	RI (RU)	Chi² (RU²)	U-value
	2.828E+6	0.03638	1.286E-8	51.17		3.986E+6				1.30	12
Cycle: 2					1.280E-9		30.00	1.239E+7	0.4315		
					3.200E-9				0.7659		
					8.000E-9				1.955		
					2.000E-8				2.749		
					5.000E-8				1.725		

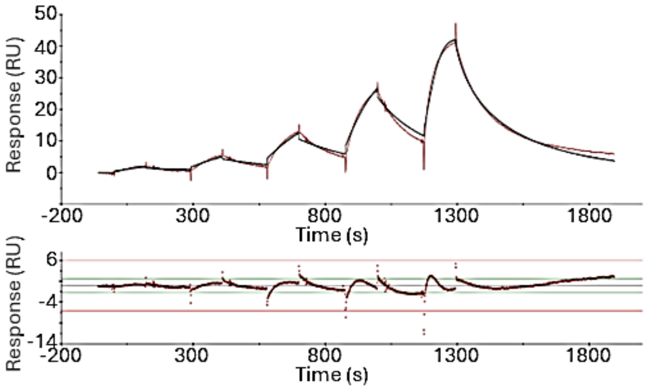

Chip 2

Quality Control

✗	Reported kinetic constant kd is outside and kinetic constant ka is approaching the limits that can be measured by the instrument.
✗	Kinetic constants cannot be uniquely determined.
!	High bulk contributions (RI) found.
→	Check that sensorgrams have sufficient curvature.
→	Examine the residual plot. Pay attention to systematic and non-random deviations.

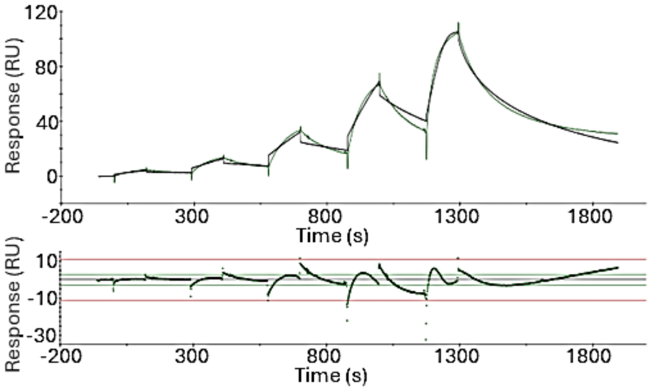

Supplementary Figure S10B: *SPR: αGFP/GFP interaction in PBST+C. Individual replicates, corresponding fits, SPR quality control (QC) information and kinetic parameters.*

Evaluation for chip 2 did not pass SPR QC. Therefore, kinetic parameters (αGFP/GFP interaction, PBST+C, Chip 2) were not included for the kinetic comparison study.

Chip 1

Quality Control

✓	Reported kinetic constants are within instrument specifications.
✓	Kinetic constants appear to be uniquely determined.
✓	No significant bulk contributions (RI) found.
➡	Check that sensorgrams have sufficient curvature.
➡	Examine the residual plot. Pay attention to systematic and non-random deviations.

Report table

Curve	ka (1/Ms)	kd (1/s)	KD (M)	Rmax (RU)	Conc (M)	tc	Flow (ul/min)	kt (RU/Ms)	RI (RU)	Chi² (RU²)	U-value
	1.651E+6	0.01988	1.204E-8	56.86		4.977E+6				1.58	7
Cycle: 2					1.280E-9		30.00	1.546E+7	0.6079		
					3.200E-9				1.012		
					8.000E-9				2.000		
					2.000E-8				2.825		
					5.000E-8				2.815		

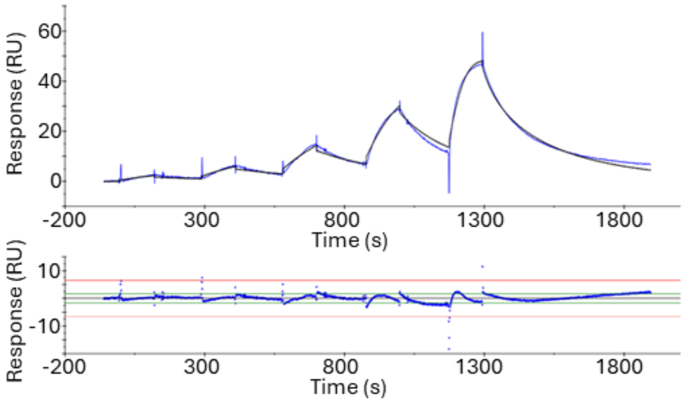

Chip 2

Quality Control

✓	Reported kinetic constants are within instrument specifications.
✓	Kinetic constants appear to be uniquely determined.
!	High bulk contributions (RI) found.
➡	Check that sensorgrams have sufficient curvature.
➡	Examine the residual plot. Pay attention to systematic and non-random deviations.

Report table

Curve	ka (1/Ms)	kd (1/s)	KD (M)	Rmax (RU)	Conc (M)	tc	Flow (ul/min)	kt (RU/Ms)	RI (RU)	Chi² (RU²)	U-value
	1.560E+6	0.01404	9.003E-9	128.8		1.191E+7				6.60	5
Cycle: 2					1.280E-9		30.00	3.701E+7	1.385		
					3.200E-9				3.414		
					8.000E-9				7.015		
					2.000E-8				8.647		
					5.000E-8				6.587		

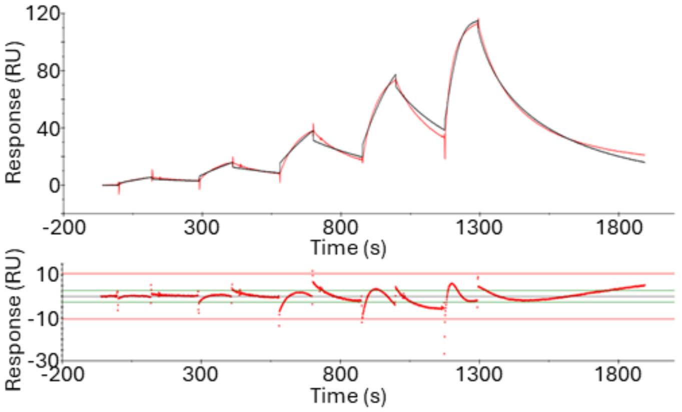

Supplementary Figure S10C: *SPR: αGFP/GFP interaction in T.MACS^{TM}. Individual replicates, corresponding fits, SPR quality control (QC) information and kinetic parameters.*

All evaluations passed SPR QC.

Chip 1

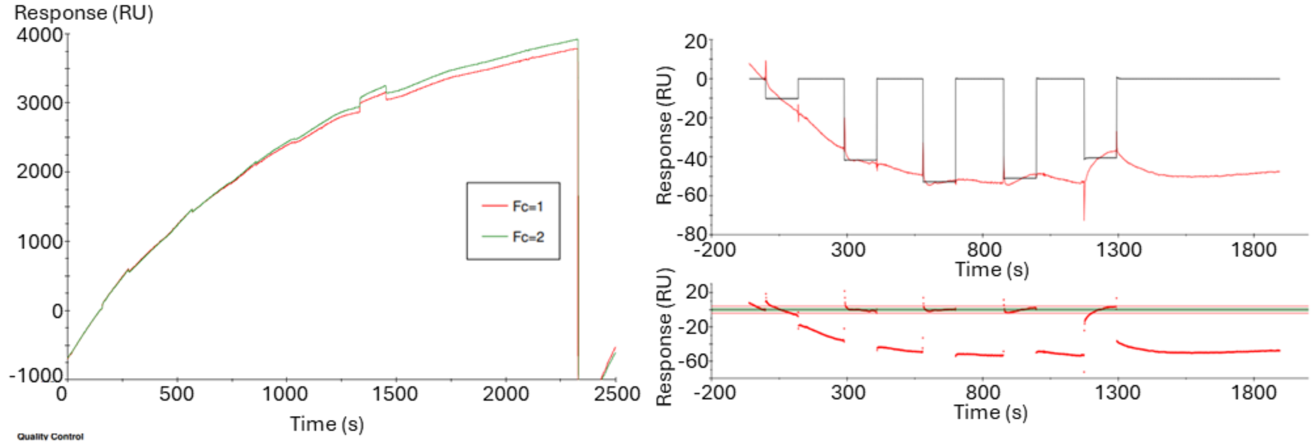

Quality Control

Reported kinetic constant kd is outside the limits that can be measured by the instrument.
Kinetic constants cannot be uniquely determined.
High bulk contributions (RI) found.
Check that sensorgrams have sufficient curvature.
Examine the residual plot. Pay attention to systematic and non-random deviations.

Chip 2

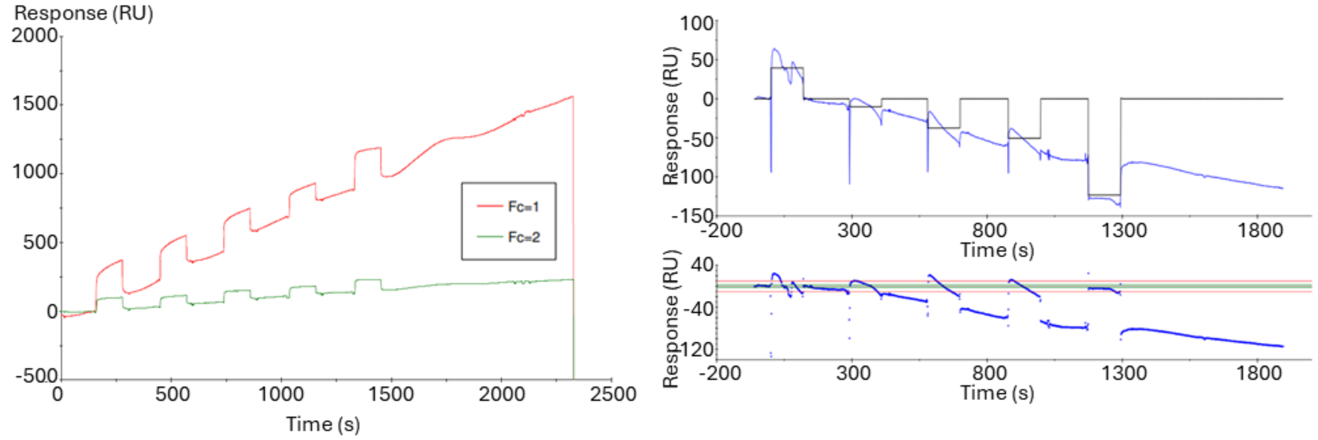

Quality Control

Reported kinetic constant kd is outside the limits that can be measured by the instrument.
Kinetic constants cannot be uniquely determined.
High bulk contributions (RI) found.
Check that sensorgrams have sufficient curvature.
Examine the residual plot. Pay attention to systematic and non-random deviations.

Supplementary Figure S10D: *SPR: αGFP/GFP interaction in 50% Bovine Serum (BS). Individual replicates, corresponding fits, SPR quality control (QC) information.*

Left sensorgrams: Unreferenced raw data of kinetic run of test flow cell (FC2, green) and control flow cell (FC1, red). Right sensorgrams: Double referenced data and fit. αGFP/GFP interaction in 50% BS was always carried out as last measurement on a chip. On Chip 1 a very strong drift during kinetic run is visible. On chip 1 baseline stabilization period was only 5 min. The repeat on Chip 2 with the 1 h baseline stabilization exhibited a comparably less pronounced, yet still considerable drift. Chip 1 was measured in 50% BS supplemented with 100 U/mL penicillin-streptomycin. Chip 2 was measured in 50% BS supplemented with 20 mM EDTA, 1x PI and 0.02% NaN_3. Due to the observed drift during kinetic measurement the evaluation of double referenced fits did not pass SPR QC for both chips. Therefore, kinetic parameters were not included for the kinetic comparison study.

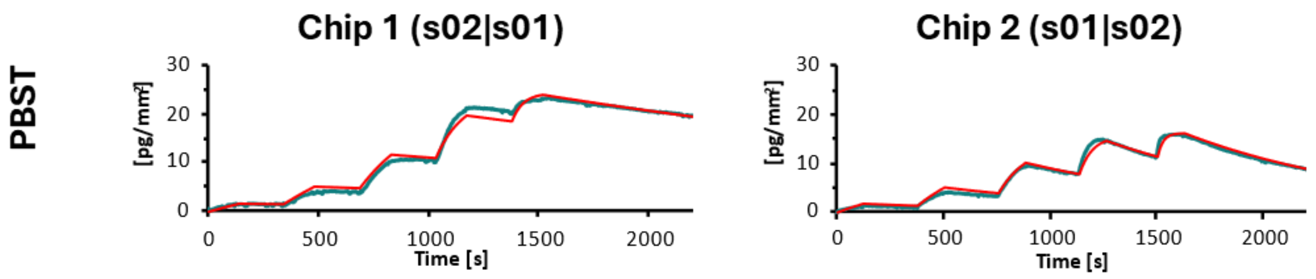

	Rep 1 (E13)	Rep 2 (G15)
KD (M)	6.62E-10	1.36E-09
kon(1/Ms)	3.92E+05	8.36E+05
kdis(1/s)	2.54E-04	1.11E-03

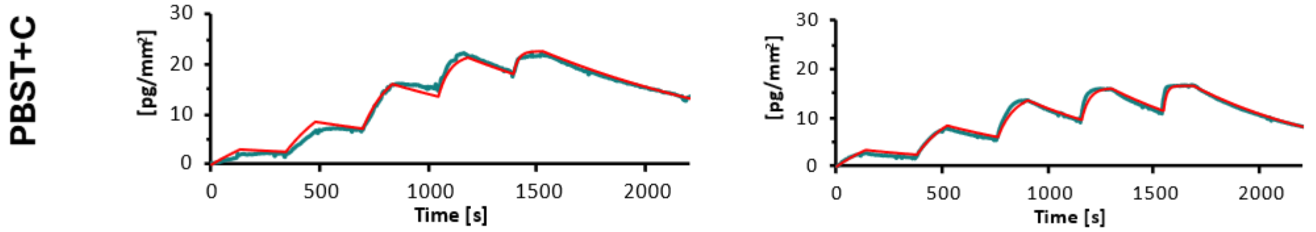

	Rep 1 (E13)	Rep 2 (G15)
KD (M)	8.47E-10	1.04E-09
kon(1/Ms)	9.06E+05	1.25E+06
kdis(1/s)	7.55E-04	1.31E-03

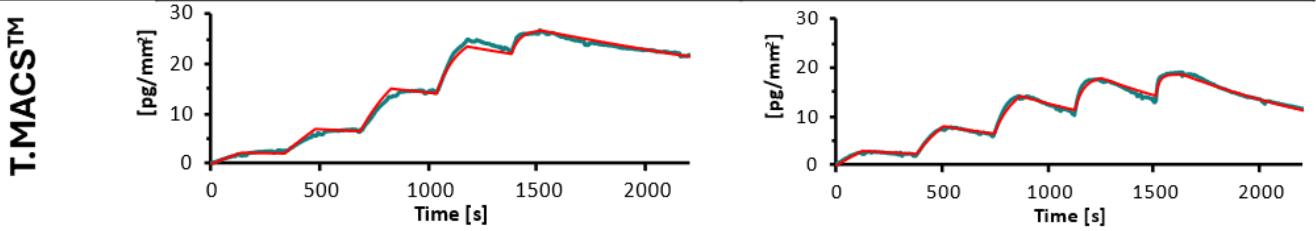

	Rep 1 (E13)	Rep 2 (G15)
KD (M)	5.97E-10	7.68E-10
kon(1/Ms)	5.06E+05	1.02E+06
kdis(1/s)	3.02E-04	7.65E-04

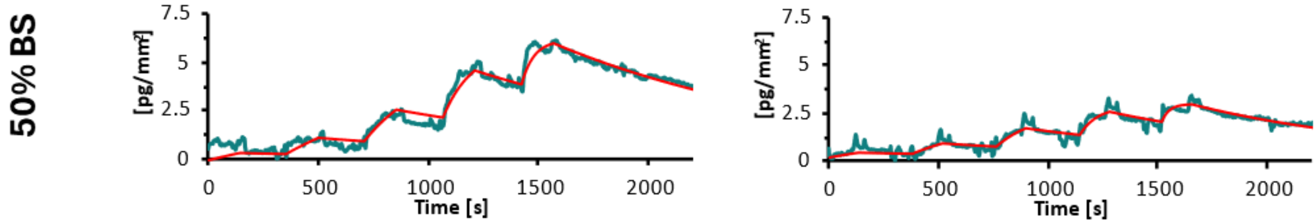

	Rep 1 (E13)	Rep 2 (G15)
KD (M)	2.08E-09	2.08E-09
kon(1/Ms)	4.02E+05	5.51E+05
kdis(1/s)	8.41E-04	1.14E-03

Supplementary Figure S11: *FM: αGFP/GFP interaction. Representative sensorgrams of individual replicates corresponding fits and kinetic parameters.*

Two individual chips, with inversed mologram architecture (s02|s01 vs s01|s02) were functionalized with αGFP-V_HH-conjugates and backfilled with NC-V_HH-conjugates. For FM αGFP/GFP interaction analyses, regenerated chips were re-used for kinetic characterization in the different kinetic buffers (PBST, PBST+C, T.MACS™ and 50% Bovine Serum) using fresh V_HH-conjugates (representative workflow shown in Supplementary Figure S3). Kinetic parameters correspond to the median kinetic parameters obtained by analysis of all 54 molograms on a single chip. Note that they do not necessarily correspond to the values of the median mologram seen in the graphs as each kinetic parameter has its own median and the Figure shows the mologram with median signal intensity.

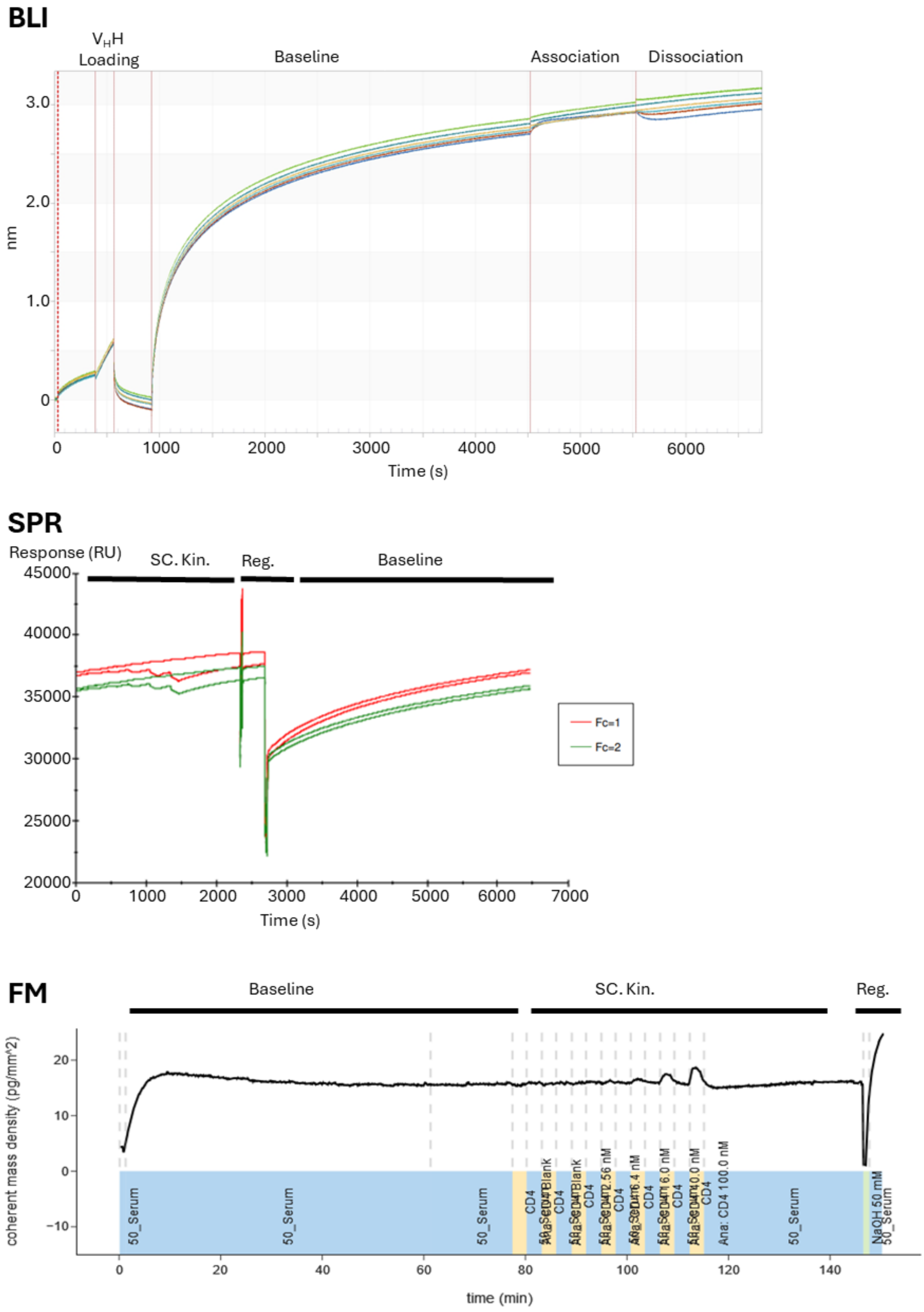

Supplementary Figure S12: *Baseline drift of 50% Bovine Serum (BS) on BLI, SPR and FM (αCD4/sCD4 interaction).*

Representative sensorgrams of raw data of the αCD4/sCD4 interaction in 50% Bovine Serum.

BLI shows a clear drift in the baseline. This drift is a lot higher than the signal of the binding, also the drift goes on even after 1 hour and there is a signal increase even in the dissociation part. Like BLI, strong baseline drift is observed for SPR and doesn't stabilize, even after 1 hour of stabilization phase. FM also shows an increase in signal at the beginning when the chip is being primed with serum but after 1 hour the signal is very stable.

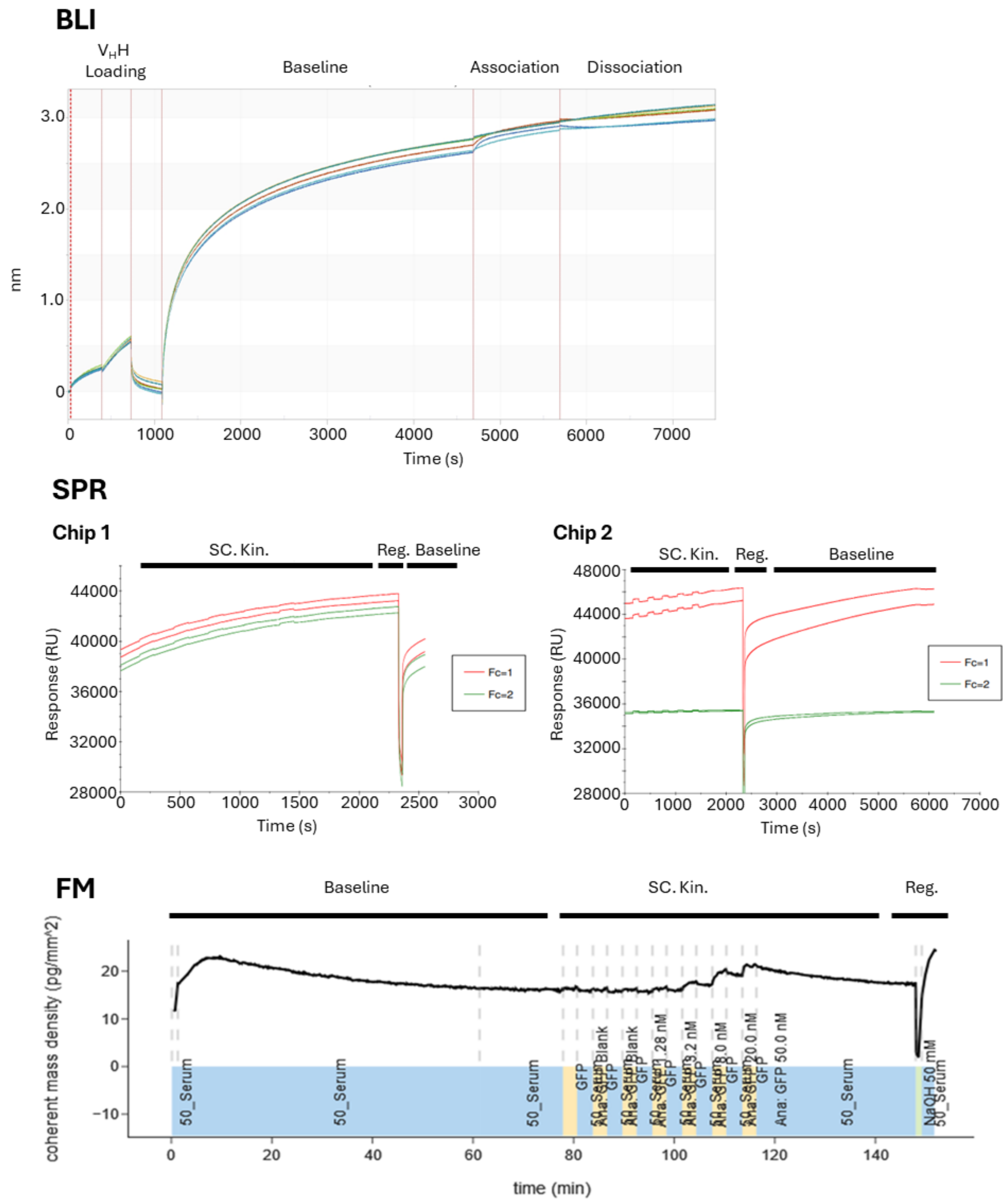

Supplementary Figure S13: *Baseline drift of 50% Bovine Serum (BS) on BLI, SPR and FM (αGFP/GFP interaction).*

Representative sensorgrams of raw data of the αGFP interaction in 50% Bovine Serum.

BLI shows a clear drift in the baseline. This drift is a lot higher than the signal of the binding, also the drift goes on even after 1 hour and there is a signal increase even in the dissociation part. Like BLI, strong baseline drift is observed for SPR and doesn't stabilize, even after 1 hour of stabilization phase (Chip 2). For SPR Chip 1 the baseline stabilization phase was shorter (5 min) but drift can still be observed during following kinetic phase. FM also shows an increase in signal at the beginning when the chip is being primed with serum but after 1 hour the signal is very stable.

BLI

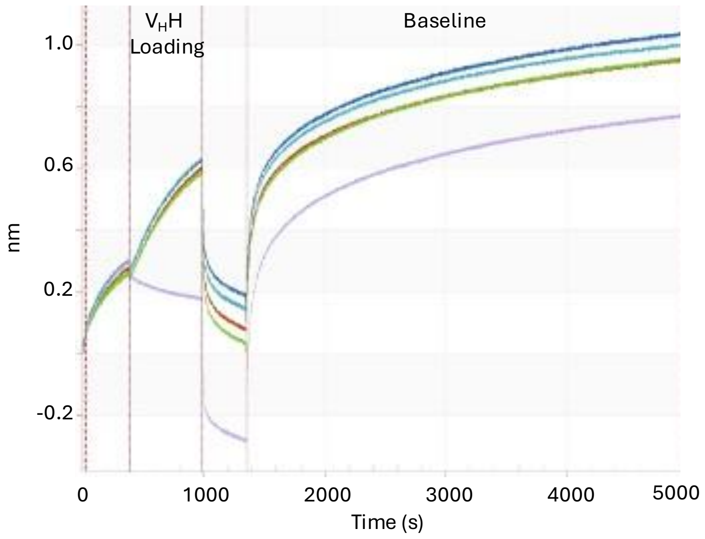

FM

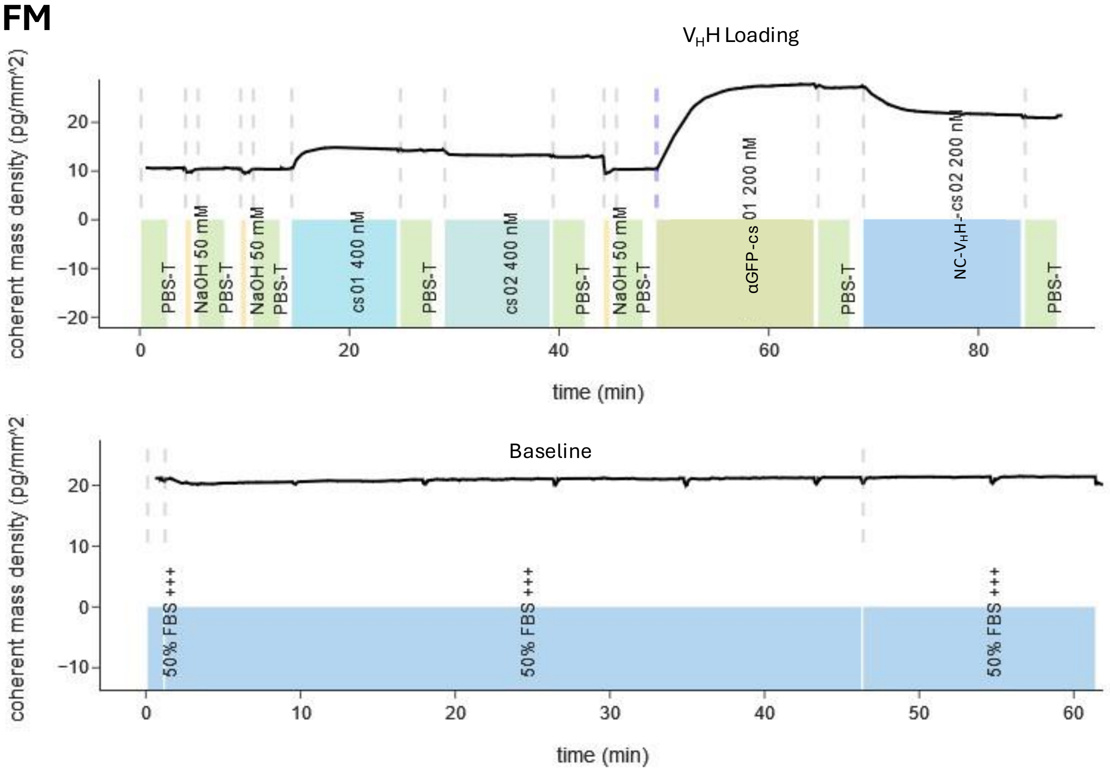

Supplementary Figure S14: *Baseline drift of 50% Fetal Bovine Serum (FBS) on BLI, and FM.*

Representative sensorgrams of raw data using αGFP-V$_H$H functionalized sensors (BLI) or αGFP-V$_H$H functionalized ridges backfilled with NC-V$_H$H (FM) with 50% FBS with additives (20 mM EDTA, 1x PI and 0.02% NaN3). BLI shows a clear drift in the baseline. FM shows no baseline drift.

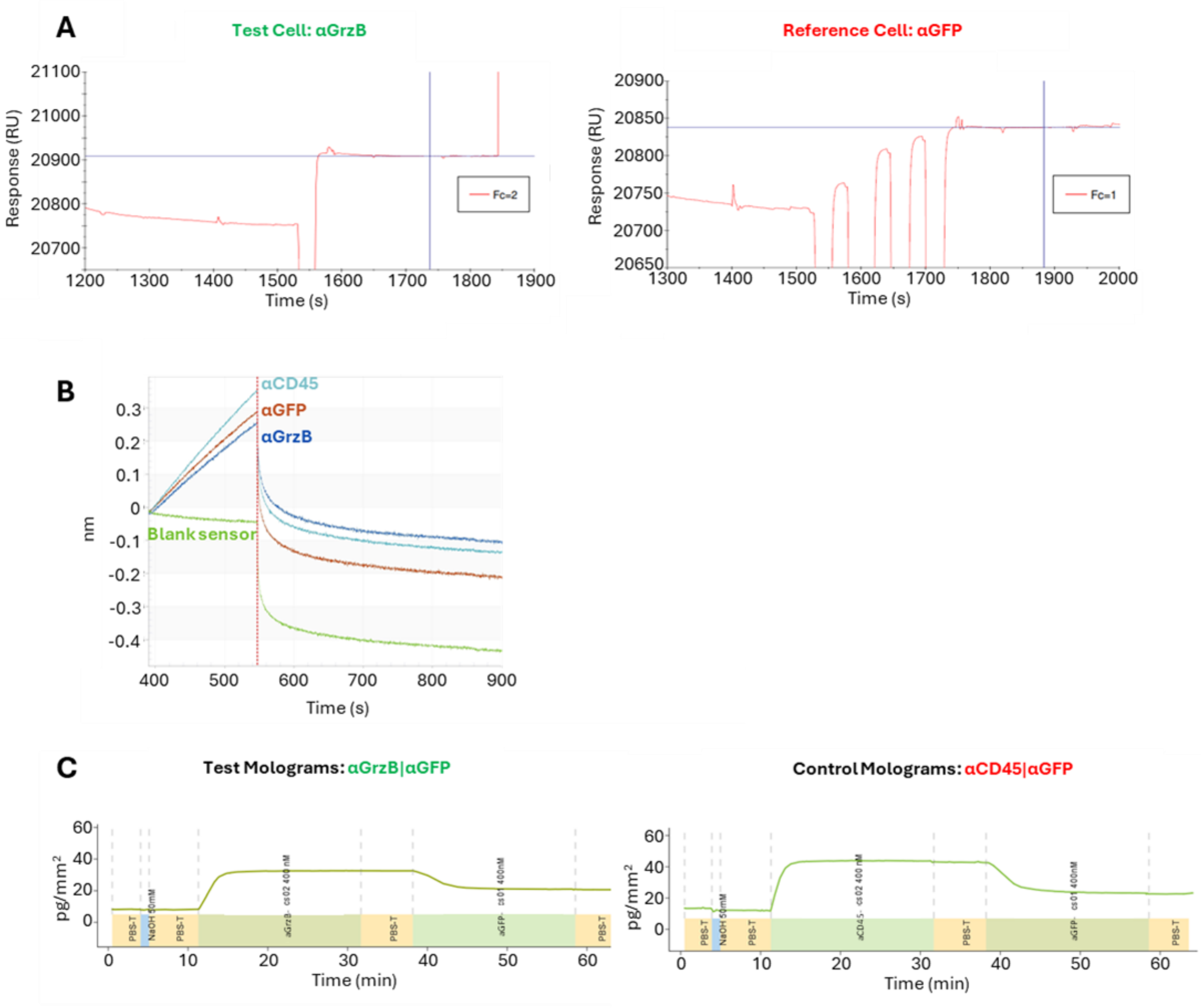

Supplementary Figure S15: *Affinity matching of reference and control surfaces on SPR, BLI and FM.*

A) SPR: Reference Flow cell (red) was biochemically matched to αGrzB test cell (green) by immobilization of a control V_HH (αGFP) to reach a similar surface density of 100 RU.
B) BLI: αGrzB-V_HH was immobilized on test sensor. Control sensors were biochemically matched by immobilization of control V_HH (αGFP, αCD45) to reach a similar surface density of $\Delta\lambda$ = 0.3 nm. Additionally, a non-matched blank sensor was included.
C) FM: Using a double flow cell test and control molograms were individually functionalized. Test molograms ridges were functionalized with αGrzB-V_HH and grooves were affinity matched by backfilling with αGFP-V_HH (αGrzB|αGFP). Negative control molograms were functionalized with αCD45-V_HH and backfilled with αGFP-V_HH (αCD45|αGFP).

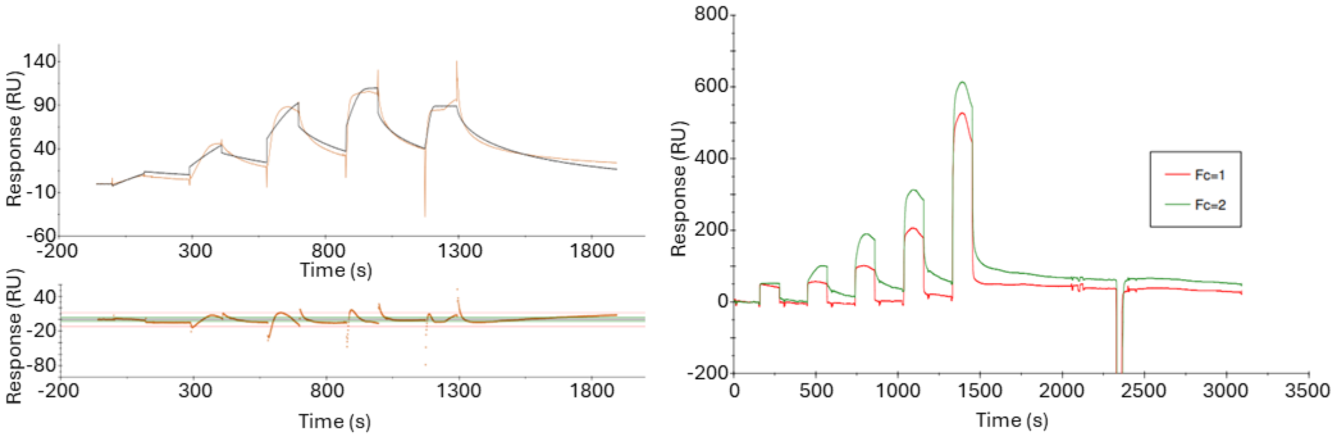

Quality Control

Reported kinetic constants ka and kd are outside the limits that can be measured by the instrument.
Kinetic constants cannot be uniquely determined.
High bulk contributions (RI) found.
Check that sensorgrams have sufficient curvature.
Examine the residual plot. Pay attention to systematic and non-random deviations.

Supplementary Figure S16: *Representative sensorgrams of analysed and raw SPR data of Granzyme B (GrzB) non-specific binding in T.MACS^{TM} using an affinity matched reference flow cell.*

Left: Double referenced data and fit. Right: Raw data of the GrzB kinetic run. Bottom: Corresponding SPR quality control (QC). GrzB concentrations that were injected were 12.5 nM, 25 nM, 50 nM, 100 nM and 200 nM. Test flow cell (FC2, green) was immobilized with αGrzB–V$_H$H. Reference flow cell (FC1, red) was immobilized with αGFP-V$_H$H (Compare Supplementary Figure S15). The raw data from kinetic runs demonstrated strong non-specific binding to the reference cell (red, FC1, αGFP). Upon processing the data by double referencing, a pattern resembling concentration dependent kinetics is observed. However, evaluation of kinetic constants failed SPR QC («Kinetic constants cannot be uniquely determined, Reported kinetic constants k_{on} and k_{off} are outside the limits that can be measured by the instrument») and therefore analysis did not result in reliable kinetic constants.

A) Raw data

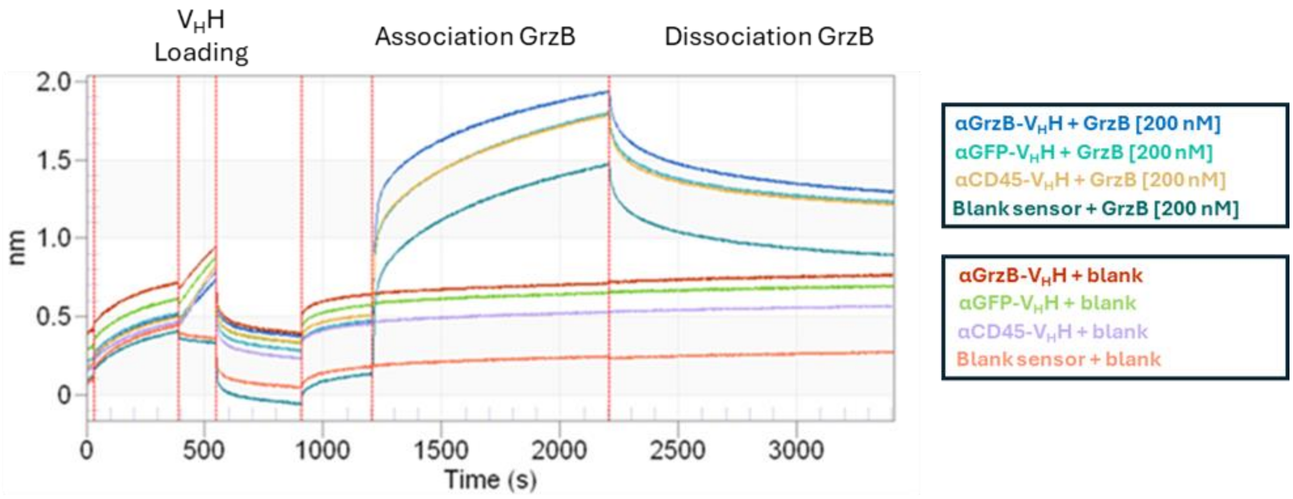

B) 1:1 Local full fit

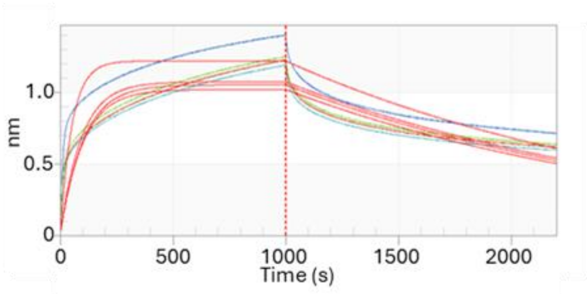

Loading Sample ID	K_D (M)	k_{on}(1/Ms)	**k_{off}(1/s)**
αGrzB-V_HH	6.33E-09	9.20E+04	**5.82E-04**
αGFP-V_HH	1.27E-08	4.60E+04	**5.82E-04**
αCD45-V_HH	1.26E-08	4.69E+04	**5.89E-04**
Blank sensor	1.13E-08	5.06E+04	**5.71E-04**

C) 1:1 Local partial fit

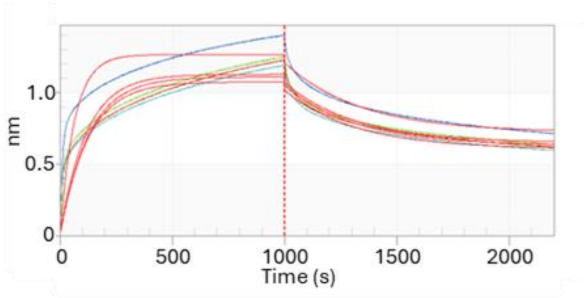

Loading Sample ID	K_D (M)	k_{on}(1/Ms)	**k_{off}(1/s)**
αGrzB-V_HH	4.81E-08	6.23E+04	**2.99E-03**
αGFP-V_HH	1.35E-07	2.36E+04	**3.17E-03**
αCD45-V_HH	1.31E-07	2.42E+04	**3.17E-03**
Blank sensor	1.19E-07	2.70E+04	**3.21E-03**

Supplementary Figure S17: *Representative sensorgrams of analysed and raw BLI data of GrzB non-specific binding in T.MACS™ using affinity matched control sensors.*

A) Raw data. B) 1:1 local full fit after single referencing. C) 1:1 local partial fit after single referencing.

The GrzB concentrations that was used, was 200 nM. Test sensors were immobilized with αGrzB–V_HH. Control sensors were immobilized with αGFP-V_HH and αCD45-V_HH, additionally a blank sensor was used as control (Compare Supplementary Figure S15). For single referencing a blank sample was used for each test or control sensor. Non-specific binding of GrzB was observed for all controls.
Independent of fitting model, obtained K_D and kinetic parameters from control sensors were almost identical to the ones obtained from test sensors. This was especially pronounced for k_{off}. Therefore, we conclude that the obtained values for the αGrz/GrzB interaction on BLI are not reliable due to masking of the signal due to non-specific interaction of GrzB with BLI sensors.

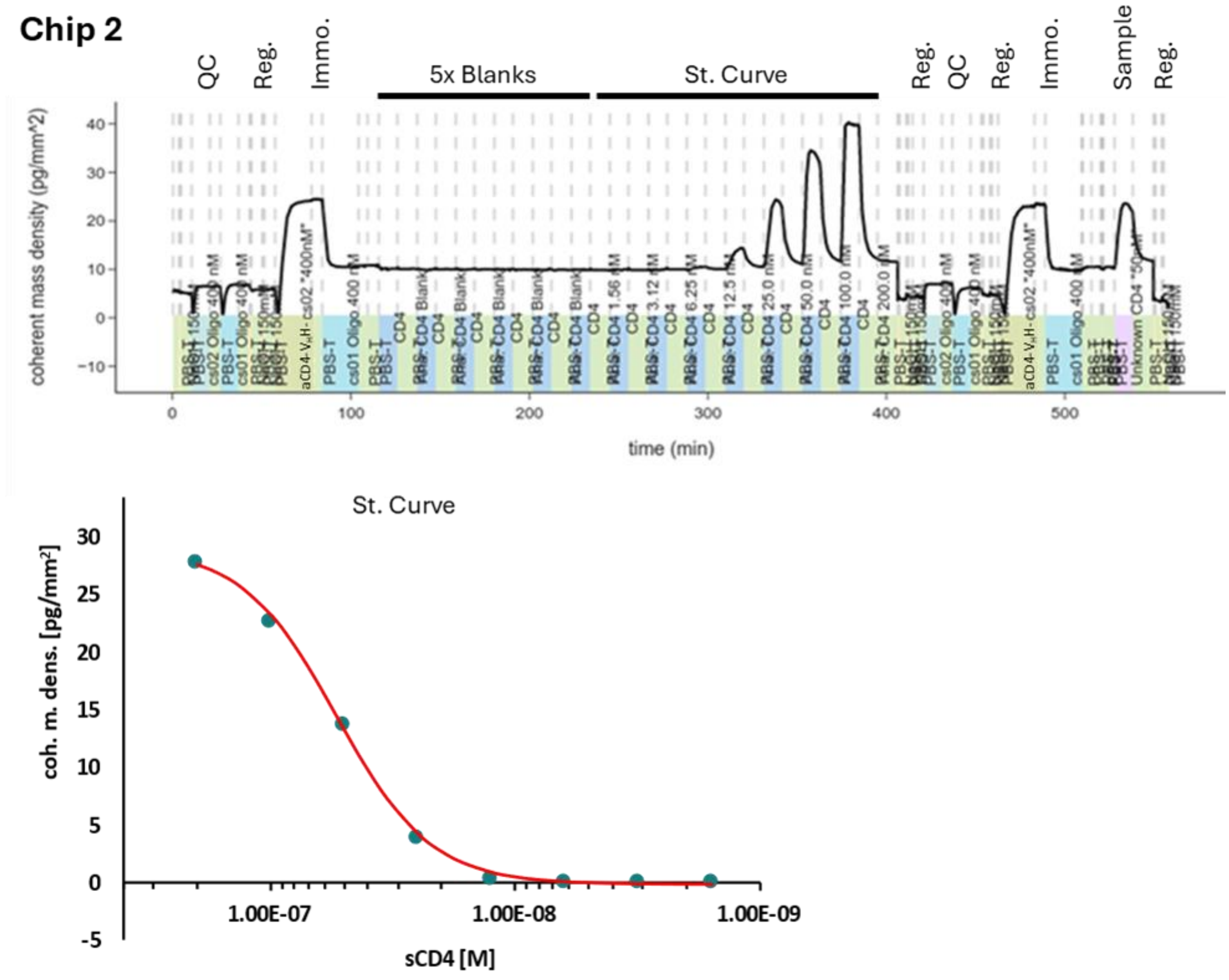

Supplementary Figure S18: *Representative sensorgram (top) and standard curve (bottom) of sCD4 quantification assay replicate in T.MACS^{TM} cell media on FM.*

Measurement was carried out in grooves on s01|s02 mologram architecture on chip 2

Quantification assay was conducted on 54 molograms in parallel in PBST running buffer in the following sequence: 1st Quality control (QC): To check for correct mologram architecture and ssDNA surface density, serial injections (here: cs02 and cs01) of ssDNA complementary to ssDNA functionalized in grooves (here: s02) and ridges (here: s01) were conducted followed by regeneration. 1st Immobilization of V_HH-conjugates: Target specific conjugates (here: αCD4-cs02) were immobilized in the ridges and ssDNA cs01 was used for "backfilling" the ridges. Blank measurements and standard curve: After 5 blank injections, increasing concentration of sCD4 were injected for generation of a standard curve followed by regeneration. sCD4 dilution series ranged from 1.6 nM – 200 nM. Each concentration was preceded by a baseline in PBST which was used to calculate the Δ "coherent mass density" for each sCD4 concentration. 2nd QC: Analogue to 1st QC. 2nd Immobilization: Analogue to 1st Immobilization. Injection of a spiked sCD4 sample: A spiked 50 nM sCD4 sample was injected at conditions analogue to the sCD4 samples during blank measurements and standard curve followed by regeneration.

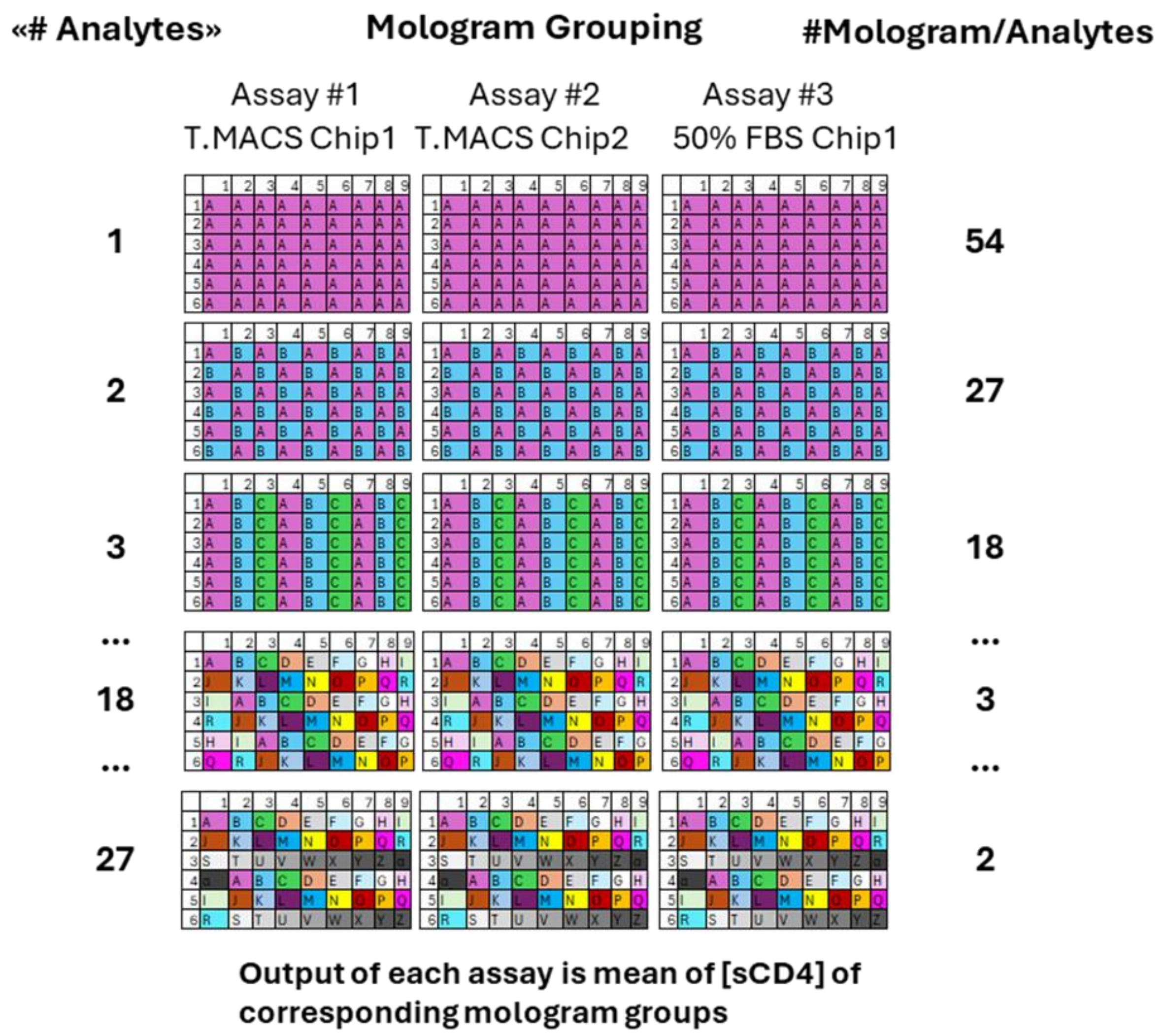

Inter Assay Analysis (n=3)

# Analytes	# Molograms /Analyte	Spiked CD4 [nM]	mean CD4 (n=3) [nM]	Recovery-1	CV (n=3)
1	54	50.0	49.5	- 0.9%	1.2%
2	27	50.0	49.3	- 1.4%	2.4%
3	18	50.0	48.8	- 2.5%	1.8%
4	13	50.0	49.2	- 1.7%	4.4%
5	10	50.0	48.4	- 3.2%	4.1%
6	9	50.0	48.1	- 3.8%	4.6%
9	6	50.0	47.7	- 4.7%	5.5%
13	4	50.0	52.4	+ 6.0%	9.6%
18	3	50.0	45.7	- 8.7%	15.2%
27	2	50.0	44.8	- 10.3%	26.2%

Supplementary Figure S19: *Multiplex exploration analysis using sCD4 quantification data.*

To investigate how many analytes could potentially be measured in parallel using FM we conducted the following procedure. Mologram positions were divided into different subgroups. Output of each individual assay was defined as the mean sCD4 concentration obtained by molograms in the corresponding groups. Inter assay analysis for recovery and CV was carried out by calculating the mean output concentration and corresponding standard deviation for each subgroup across all assays (n = 3). Recovery rates and CVs correspond to groups that exhibited the lowest performance (Compare Supplementary Figures S20 and S21 for full data). The same position grouping was carried out for each individual sCD4 assay.

	1	2	3	4	5	6	7	8	9
1	A	A	A	A	A	A	A	A	A
2	A	A	A	A	A	A	A	A	A
3	A	A	A	A	A	A	A	A	A
4	A	A	A	A	A	A	A	A	A
5	A	A	A	A	A	A	A	A	A
6	A	A	A	A	A	A	A	A	A

n = 1	Intra Exp [CD4]			Mean [CD4]	Recovery	CV
Group	*#1*	*#2*	*#3*	*Inter Exp (n=3)*		
A	50.2	48.9	49.5	49.5	99.1%	1.2%

	1	2	3	4	5	6	7	8	9
1	A	B	A	B	A	B	A	B	A
2	B	A	B	A	B	A	B	A	B
3	A	B	A	B	A	B	A	B	A
4	B	A	B	A	B	A	B	A	B
5	A	B	A	B	A	B	A	B	A
6	B	A	B	A	B	A	B	A	B

n = 2	Intra Exp [CD4]			Mean [CD4]	Recovery	CV
Group	*#1*	*#2*	*#3*	*Inter Exp (n=3)*		
A	50.5	48.1	49.3	49.3	98.6%	2.4%
B	49.8	49.7	49.7	49.7	99.5%	0.1%

	1	2	3	4	5	6	7	8	9
1	A	B	C	A	B	C	A	B	C
2	A	B	C	A	B	C	A	B	C
3	A	B	C	A	B	C	A	B	C
4	A	B	C	A	B	C	A	B	C
5	A	B	C	A	B	C	A	B	C
6	A	B	C	A	B	C	A	B	C

n = 3	Intra Exp [CD4]			Mean [CD4]	Recovery	CV
Group	*#1*	*#2*	*#3*	*Inter Exp (n=3)*		
A	51.2	49.6	49.7	50.2	100.3%	1.8%
B	49.7	48.9	50.4	49.7	99.3%	1.5%
C	49.6	48.3	48.4	48.8	97.5%	1.4%

	1	2	3	4	5	6	7	8	9
1	A	B	C	D	A	B	C	D	A
2	B	C	D	A	B	C	D	A	B
3	C	D	A	B	C	D	A	B	C
4	D	A	B	C	D	A	B	C	D
5	A	B	C	D	A	B	C	D	A
6	B	C	D	A	B	C	D		

n = 4	Intra Exp [CD4]			Mean [CD4]	Recovery	CV
Group	*#1*	*#2*	*#3*	*Inter Exp (n=3)*		
A	50.7	49.2	48.4	49.4	98.9%	2.4%
B	49.2	50.0	51.1	50.1	100.3%	1.9%
C	50.4	46.8	50.7	49.3	98.6%	4.4%
D	50.3	49.2	48.0	49.2	98.3%	2.3%

	1	2	3	4	5	6	7	8	9
1	A	B	C	D	E	A	B	C	D
2	E	A	B	C	D	E	A	B	C
3	D	E	A	B	C	D	E	A	B
4	C	D	E	A	B	C	D	E	A
5	B	C	D	E	A	B	C	D	E
6	A	B	C	D	E				

n = 5	Intra Exp [CD4]			Mean [CD4]	Recovery	CV
Group	*#1*	*#2*	*#3*	*Inter Exp (n=3)*		
A	50.5	49.5	52.9	51.0	101.9%	3.4%
B	50.7	49.7	48.1	49.5	99.0%	2.6%
C	49.7	46.4	50.0	48.7	97.4%	4.1%
D	50.3	48.0	47.0	48.4	96.8%	3.5%
E	49.8	49.8	50.2	49.9	99.9%	0.5%

	1	2	3	4	5	6	7	8	9
1	A	B	C	D	E	F	A	B	C
2	D	E	F	A	B	C	D	E	F
3	A	B	C	D	E	F	A	B	C
4	D	E	F	A	B	C	D	E	F
5	A	B	C	D	E	F	A	B	C
6	D	E	F	A	B	C	D	E	F

n = 6	Intra Exp [CD4]			Mean [CD4]	Recovery	CV
Group	*#1*	*#2*	*#3*	*Inter Exp (n=3)*		
A	51.7	49.6	47.2	49.5	99.0%	4.6%
B	49.2	49.2	48.7	49.0	98.0%	0.6%
C	49.6	46.3	48.5	48.1	96.2%	3.5%
D	50.6	49.6	52.3	50.8	101.6%	2.7%
E	49.7	48.8	52.6	50.3	100.7%	3.9%
F	49.6	50.3	48.3	49.4	98.8%	2.1%

Supplementary Figure S20: *Mologram distribution and data for multiplex exploration analysis using sCD4 quantification data for n = 1 to n = 6 groups.*

	1	2	3	4	5	6	7	8	9
1	A	B	C	D	E	F	G	H	I
2	I	A	B	C	D	E	F	G	H
3	H	I	A	B	C	D	E	F	G
4	G	H	I	A	B	C	D	E	F
5	F	G	H	I	A	B	C	D	E
6	E	F	G	H	I	A	B	C	D

n = 9	Intra Exp [CD4]			Mean [CD4]	Recovery	CV
Group	#1	#2	#3	Inter Exp (n=3)		
A	52.0	50.9	52.3	51.7	103.5%	1.4%
B	50.5	51.5	47.3	49.8	99.6%	4.4%
C	50.3	45.0	47.7	47.7	95.3%	5.5%
D	50.9	48.4	48.2	49.2	98.3%	3.1%
E	49.5	48.7	53.3	50.5	101.0%	4.9%
F	49.7	48.7	50.0	49.5	99.0%	1.4%
G	49.3	49.1	47.2	48.5	97.1%	2.4%
H	50.0	48.6	49.9	49.5	99.0%	1.6%
I	49.2	49.2	49.6	49.3	98.7%	0.5%

	1	2	3	4	5	6	7	8	9
1	A	B	C	D	E	F	G	H	I
2	J	K	L	M	A	B	C	D	E
3	F	G	H	I	J	K	L	M	A
4	B	C	D	E	F	G	H	I	J
5	K	L	M	A	B	C	D	E	F
6	G	H	I	J	K	L	M		

n = 13	Intra Exp [CD4]			Mean [CD4]	Recovery	CV
Group	#1	#2	#3	Inter Exp (n=3)		
A	52.9	52.3	52.0	52.4	104.8%	0.9%
B	48.9	50.8	45.9	48.6	97.1%	5.1%
C	49.5	41.8	49.7	47.0	94.0%	9.6%
D	50.5	47.5	51.1	49.7	99.4%	3.9%
E	49.5	47.8	55.9	51.1	102.1%	8.3%
F	49.3	47.3	45.4	47.4	94.7%	4.1%
G	49.5	47.4	47.8	48.2	96.5%	2.4%
H	51.2	47.3	51.8	50.1	100.2%	4.9%
I	50.0	48.4	48.4	48.9	97.8%	1.8%
J	50.4	50.8	48.3	49.8	99.7%	2.7%
K	50.5	49.5	48.1	49.4	98.7%	2.4%
L	50.3	52.3	51.8	51.5	103.0%	2.1%
M	49.4	51.1	47.9	49.5	98.9%	3.2%

	1	2	3	4	5	6	7	8	9
1	A	B	C	D	E	F	G	H	I
2	J	K	L	M	N	O	P	Q	R
3	I	A	B	C	D	E	F	G	H
4	R	J	K	L	M	N	O	P	Q
5	H	I	A	B	C	D	E	F	G
6	Q	R	J	K	L	M	N	O	P

n = 18	Intra Exp [CD4]			Mean [CD4]	Recovery	CV
Group	#1	#2	#3	Inter Exp (n=3)		
A	54.4	51.8	51.0	52.4	104.8%	3.4%
B	49.8	51.1	50.0	50.3	100.6%	1.3%
C	50.8	37.8	48.4	45.7	91.3%	15.2%
D	49.1	46.0	48.0	47.7	95.4%	3.2%
E	51.4	45.8	49.8	49.0	98.1%	5.9%
F	49.2	46.4	46.7	47.5	94.9%	3.3%
G	50.0	46.5	44.4	47.0	93.9%	6.1%
H	49.7	46.4	47.7	48.0	95.9%	3.5%
I	47.6	47.1	50.4	48.4	96.7%	3.6%
J	49.7	51.3	49.3	50.1	100.2%	2.2%
K	51.4	49.9	48.6	50.0	99.9%	2.7%
L	49.8	51.6	52.3	51.2	102.5%	2.5%
M	50.4	51.4	48.2	50.0	100.0%	3.3%
N	49.1	51.5	45.3	48.6	97.2%	6.5%
O	50.7	51.8	48.8	50.4	100.8%	3.0%
P	50.2	51.3	53.8	51.8	103.6%	3.6%
Q	48.9	50.5	56.4	51.9	103.9%	7.5%
R	50.4	52.0	52.2	51.5	103.0%	1.9%

	1	2	3	4	5	6	7	8	9
1	A	B	C	D	E	F	G	H	I
2	J	K	L	M	N	O	P	Q	R
3	S	T	U	V	W	X	Y	Z	α
4	α	A	B	C	D	E	F	G	H
5	I	J	K	L	M	N	O	P	Q
6	R	S	T	U	V	W	X	Y	Z

n = 27	Intra Exp [CD4]			Mean [CD4]	Recovery	CV
Group	#1	#2	#3	Inter Exp (n=3)		
A	56.3	52.6	56.3	55.1	110.1%	3.9%
B	49.0	50.6	48.6	49.4	98.8%	2.2%
C	49.6	31.5	53.5	44.8	89.7%	26.2%
D	50.4	43.9	50.2	48.2	96.3%	7.7%
E	49.9	44.4	52.1	48.8	97.6%	8.2%
F	50.4	44.2	47.3	47.3	94.6%	6.6%
G	50.2	43.8	46.0	46.6	93.3%	6.9%
H	49.7	44.2	51.5	48.5	96.9%	7.9%
I	48.6	45.2	47.2	47.0	94.0%	3.6%
J	48.6	51.3	57.0	52.3	104.6%	8.2%
K	51.7	49.3	56.0	52.4	104.7%	6.5%
L	52.1	52.9	54.5	53.2	106.3%	2.4%
M	48.6	50.5	44.4	47.8	95.7%	6.4%
N	48.3	51.1	43.0	47.5	95.0%	8.7%
O	50.9	52.1	51.5	51.5	103.0%	1.2%
P	50.0	51.3	52.6	51.3	102.6%	2.5%
Q	50.1	50.7	49.2	50.0	100.0%	1.5%
R	48.0	51.8	62.8	54.2	108.4%	14.1%
S	50.4	49.9	49.3	49.8	99.7%	1.1%
T	49.6	51.4	39.9	47.0	94.0%	13.1%
U	51.0	51.0	41.9	47.9	95.9%	11.0%
V	51.1	50.7	50.2	50.7	101.3%	0.9%
W	50.3	50.9	51.4	50.9	101.7%	1.1%
X	49.8	50.2	43.2	47.8	95.5%	8.3%
Y	49.9	51.5	44.9	48.8	97.5%	7.1%
Z	50.0	51.2	50.6	50.6	101.2%	1.1%
α	49.7	52.3	41.8	47.9	95.9%	11.4%

Supplementary Figure S21: *Mologram distribution and data for multiplex exploration analysis using sCD4 quantification data for n = 9 to n = 27 groups*

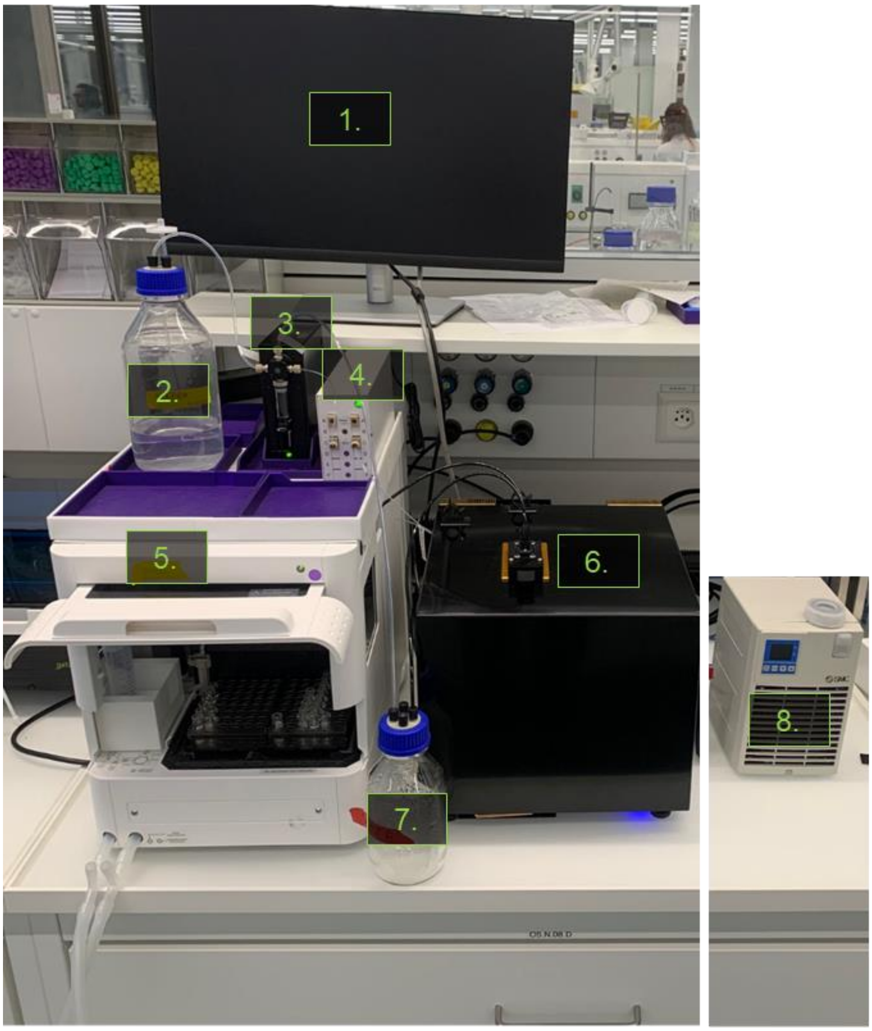

Supplementary Figure S22: *Instrument Setup of FM.*

This figure illustrates the complete setup of instruments used for FM measurements. Setup consist of following components: 1. Screen for the FM experiment manager, 2. Bottle containing running buffer, 3. Pump, 4. Degasser, 5. Autosampler, 6. FM "Callisto" reader, 7. Waste bottle, 8. SMC Thermo-con/Compact Type connected to the FM "Callisto" reader to regulate the temperature during measurements.

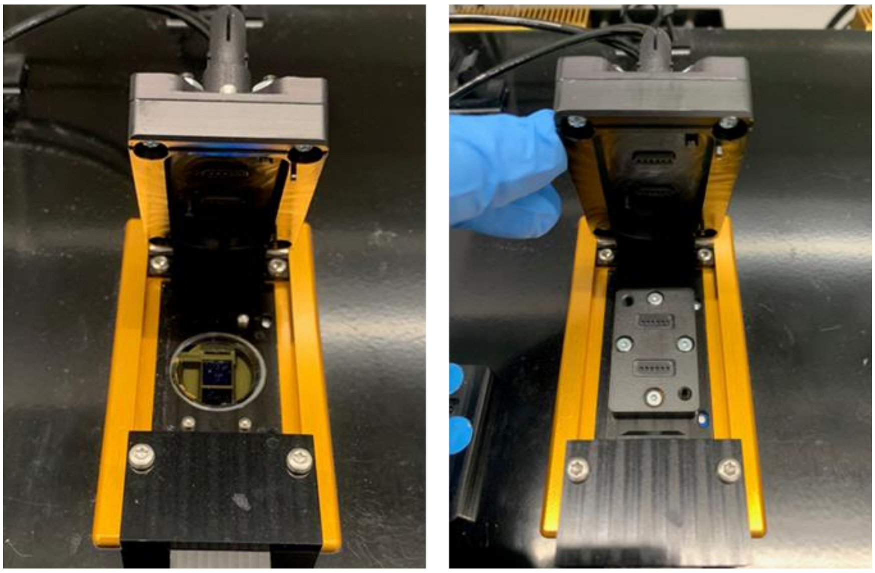

Supplementary Figure S23: *Top lid of FM "Callisto" reader.*

The top lid of the FM "Callisto" reader is designed to accommodate the flow-cell chamber. Left: without the flow-cell chamber. Right: With the flow-cell chamber inserted. The flow-cell chamber consists of two parts: an upper part that includes the flow cell and its corresponding seal, and a lower part for inserting FM chips functionalized with an array of 54 molograms. The flow-cell chamber is held together by 4 screws

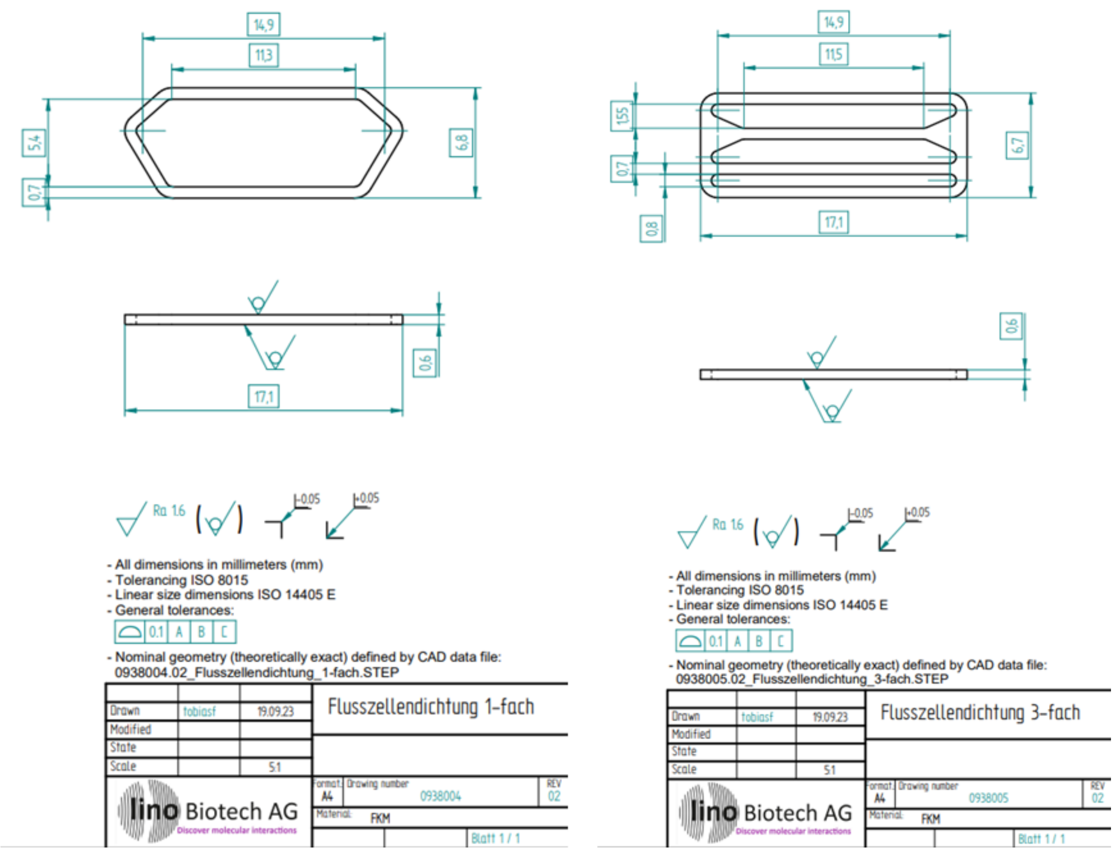

Supplementary Figure S24: *Dimensions of flow-cell sealings of different flow cells.*

Sealing of a single flow chamber (left) and double flow chamber (right). «Multi-flow» chambers allow different immobilization on the same chip.

References:

1. Apiyo, D. *Application Note: Biomolecular Binding Kinetics Assays on the Octet® BLI Platform*; Sartorius, 2022. AN-4014 Rev D.
2. Pattnaik, P. Surface Plasmon Resonance. *Appl. Biochem. Biotechnol.* **2005**, *126*, 79–92, doi:10.1385/ABAB:126:2:079.
